# VacSol: a high throughput *in silico* pipeline to predict potential therapeutic targets in prokaryotic pathogens using subtractive reverse vaccinology

**DOI:** 10.1186/s12859-017-1540-0

**Published:** 2017-02-13

**Authors:** Muhammad Rizwan, Anam Naz, Jamil Ahmad, Kanwal Naz, Ayesha Obaid, Tamsila Parveen, Muhammad Ahsan, Amjad Ali

**Affiliations:** 1 Research Center for Modelling and Simulation (RCMS), National University of Sciences and Technology (NUST), H-12, Islamabad, Pakistan; 2 Atta-ur-Rahman School of Applied Biosciences (ASAB), National University of Sciences and Technology (NUST), H-12, Islamabad, Pakistan; 3 Biosciences Department, COMSATS Institute of Information Technology, Islamabad, Pakistan

**Keywords:** Reverse vaccinology, Computational pipeline, Vaccine candidates, Subtractive proteomics, PVCs, VacSol

## Abstract

**Background:**

With advances in reverse vaccinology approaches, a progressive improvement has been observed in the prediction of putative vaccine candidates. Reverse vaccinology has changed the way of discovery and provides a mean to propose target identification in reduced time and labour. In this regard, high throughput genomic sequencing technologies and supporting bioinformatics tools have greatly facilitated the prompt analysis of pathogens, where various predicted candidates have been found effective against certain infections and diseases. A pipeline, VacSol, is designed here based on a similar approach to predict putative vaccine candidates both rapidly and efficiently.

**Results:**

VacSol, a new pipeline introduced here, is a highly scalable, multi-mode, and configurable software designed to automate the high throughput *in silico* vaccine candidate prediction process for the identification of putative vaccine candidates against the proteome of bacterial pathogens. Vaccine candidates are screened using integrated, well-known and robust algorithms/tools for proteome analysis, and the results from the VacSol software are presented in five different formats by taking proteome sequence as input in FASTA file format. The utility of VacSol is tested and compared with published data and using the *Helicobacter pylori* 26695 reference strain as a benchmark.

**Conclusion:**

VacSol rapidly and efficiently screens the whole bacterial pathogen proteome to identify a few predicted putative vaccine candidate proteins. This pipeline has the potential to save computational costs and time by efficiently reducing false positive candidate hits. VacSol results do not depend on any universal set of rules and may vary based on the provided input. It is freely available to download from: https://sourceforge.net/projects/vacsol/.

**Electronic supplementary material:**

The online version of this article (doi:10.1186/s12859-017-1540-0) contains supplementary material, which is available to authorized users.

## Background

*In silico* prediction of vaccine candidates has great significance in various life science disciplines, including biomedical research [1]. The conventional approach of vaccine development requires pathogenic cultivation in vitro that is not always possible. Although this methodology has the potential to produce successful vaccines and has long been in practice, but now considered time-consuming and inadequate for most pathogens. This caveat is particularly evident when microbes are inactive, protective, or even in the case where antigen expression is decreased; rendering the conventional approach a significant challenge for putative vaccine candidate discovery [2, 3]. These basic problems have led scientists to develop new vaccinology approaches based on advanced computational tools. In particular, with the introduction of high-throughput sequencing techniques over the last decade and the advent of bioinformatics approaches, Rino Rappouli revolutionized Pasteur’s vaccinology procedure by introducing a novel “reverse vaccinology” method [4–6]. This advanced *in-silico* technique for vaccine prediction couples genomic information and analysis with bioinformatics tools. Using this approach, several vaccines have been successfully developed against microbial pathogens [7–9]. Reverse vaccinology is now recognized as safer and more reliable as compared to conventional vaccinology methods [10, 11].

Using the reverse vaccinology approach, various predictive and analytical tools (Vaxign, VaxiJen, JennerPredict) have been designed for the identification of putative vaccine candidates. These tools are widely available online [12–14], but only a handful of softwares and pipelines, like NERVE and Vacceed [15, 16], are accessible as full packages. Although web-based pipelines are efficient, their drawbacks include time delays and constraints for input file size.

NERVE (New Enhanced Reverse Vaccinology Environment), a Perl based modular pipeline for *in-silico* identification of potential vaccine candidates, generates results through text interface configuration and is an efficient, modular-based standalone software for vaccine candidate identification [15]. But it only focuses on adhesion proteins whereas several non-adhesion proteins can also participate in host-pathogen interactions (including porin, flagellin, invasin, etc.), and most of them are pathogenic as well as antigenic. Therefore, there exists a perilous need for an updated and advanced analysis tool that inclusively provides every putative candidate in its output.

Vacceed is another highly configurable architecture designed to perform high throughput *in silico* identification of eukaryotic vaccine candidates. Vacceed is, in fact, able to reduce false vaccine candidates that are selected for laboratory validation to save time and money [16], but this highly efficient, scalable, and configurable program provides limited information on pathogenicity and putative functional genes. These main parameters prove instrumental in the determination of potential vaccine candidates. Thus, given the current software limitations, we sought to utilize the reverse vaccinology approach to overcome limitations of currently available pipelines.

We therefore focused on *in silico* reverse vaccinology approach to address the issues that were present in previous pipelines, and to precisely screen out the putative vaccine candidates from whole bacterial genome *in silico*. We designed a new automated pipeline, termed VacSol, to efficiently screen for the therapeutic vaccine agents from the bacterial pathogen proteome to save both time and resources.

## Implementation

**Fig. 1:**
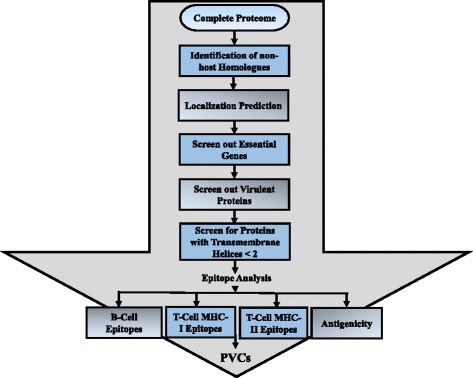
Schematic diagram of the protein prioritization process. Steps to prioritize proteins to identify PVCs include: (1) the complete bacterial proteome (sequences) subjected to the VacSol pipeline for identifying PVCs; (2) the complete proteome is searched for non-host homologous, essential, virulent proteins residing in the extracellular membrane with less than two transmembrane helices; (3) proteins that meet the selection criteria are considered to be PVC proteins; (4) prioritized proteins are further analyzed for antigenic B- and T-cell epitopes

VacSol was designed to screen and detect prioritized proteins as vaccine candidates, and its functionality is presented in Fig. 1. Notably, this software was developed on platform independent Java language, is highly flexible through one executable .jar file, and does not require any software installation. The VacSol functionality does depend on the installation of various tools that are used as pre-requisites for the pipeline execution (such as PSORTb for localization prediction), and we have integrated various freely available, well-performing and updated tools in the VacSol pipeline to achieve optimal performance. VacSol has been tested and analyzed to be fully functional on Ubuntu 12.04.5 (64 bit) version. It can also work on any operating system with already installed and functional prerequisite tools, given minor modifications. PSORTb [17] and OSDDlinux (http://osddlinux.osdd.net/) have also been pre-packaged for robust and user-friendly installation (See installation guide).

**Fig. 2:**
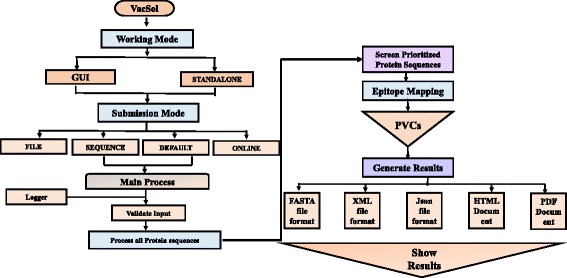
Schematic diagram of VacSol processing. VacSol is comprised of two working modes: (i) GUI, and (ii) Standalone. The software is highly flexible as it permits users to submit a FASTA proteome sequence in four different ways. Submitted input is validated through a FASTA validator, and then VacSol screens the whole proteome to prioritize proteins that have antigenic B- and T-cell epitopes. Individual tool results (Localizer, Blaster, Helicer, and Epitoper) and complete results are generated for prioritized proteins in five different formats

VacSol also offers the user to select either a single tool (selective) or complete pipeline to predict potential vaccine candidates (PVCs). Protein sequences are subjected to the main analytical process where the input format is validated through the FASTA Validator for vaccine target prediction. This main process is multi-threaded, as one can run as many threads as there are cores available in their system. Further, the pipeline is capable of processing multiple sequences in parallel. The process of sequence prioritization is performed in a number of steps to prioritize the input sequences, and is elaborated in Fig. 2. Each step is forwarded by a special script and protein sequences are screened at every step indistinctly with generated results displayed in various formats. After processing all the sequences of an input file, the prioritized sequences are then subjected for epitope mapping. Thereafter, all prioritized sequences are again directed to thread pool processing to generate final results. Final results are engendered in five different formats (FASTA, XML, JSon, HTML, and PDF format), ensuring the expandability and scalability of the designed pipeline for users. Step-wise information of VacSol is provided in a comprehensive user guide (Additional file 1).

### Distinct features

The VacSol interface is designed on four different modules: (i) Blaster, a module for predicting homology using BLASTp; (ii) Localization Predictor, predicting subcellular location; (iii) Helicer, predicting transmembrane helices; and, (iv) Epitoper, a module designed to predict B-cell and T-cell epitopes. These modules function on the basis of implemented tools (Table 1) required to screen prioritized proteins (targets). The VacSol pipeline is developed in Java, a platform independent language [18].

**Table 1:** Tools and databases integrated and implemented in VacSol

Name	Function	Source(S)
BLAST+2.2.25-7	New command line sequence alignment application developed using the NCBI C++ toolkit.	[38]
Pftools2.3	Package of programs that support the search method of generalized profile formatting.	[39]
PSORTb3.0	Protein subcellular localization prediction tool.	[17]
HMMTOP 2.0	Transmembrane topology prediction tool.	[32]
DEG 10.0	Database of essential genes.	[28]
VFDB	Virulence factors database.	[31]
ABCPred	B-Cell epitope prediction tool.	[40]
Propred-I	Prediction of promiscuous major histocompatibility complex (MHC) Class-I binding sites.	[41]
Propred	Prediction of MHC Class-II binding regions in an antigen sequence.	[42]
UniProt-SwissProt	Manually annotated protein sequences database with information extracted from literature.	[23, 33]

## Results

### Test data

VacSol performs various proteome-wide analyses and generates results in five different formats. This pipeline was validated using a sample data set of the *Helicobacter pylori* proteome. The selected strain of *H. pylori* 26695 (RefSeq NC_000915.1) is comprised of 1576 proteins or coding regions [19], and the whole proteome was scanned in each protein prioritizing step.

### Implementation of VacSol for test data

The first working step was performed by identifying the non-host homologs, required to elute host homologous proteins to restrict the chance of autoimmunity [20, 21]. Out of 1576 possible proteins, 1452 were screened as non-human homologous proteins by using BLASTp against RefSeq [22] and SwissProt [23] databases. For BLAST non-human homologs, criteria included a Bit Score >100, E-Value <1.0 e^(−5)^, and percentage identity >35% [24]. Next, these 1452 proteins were subjected for further protein prioritization processing by VacSol to predict subcellular localization. 65 proteins were found to be in the secretome and exoproteome, of which 23 proteins lie in the extracellular region, and 42 were screened as outer-membrane proteins. Prioritization of proteins according to localization substantially contributed to enhance the PVCs identification process [25]. Surface exposed proteins tend to be involved in pathogenesis, making them prime targets as vaccine candidates [26]. Similarly, both extracellular and secreted proteins are readily accessible to antibodies as compared to intracellular proteins, and therefore represent ideal vaccine candidates. Results obtained through PSORTb, and integrated in VacSol, were then cross-checked with CELLO2GO [27] to confirm the localization of putative candidate proteins. After localization validation, screened proteins were checked for their essentiality. 667 proteins were sorted as essential genes required for the survival of gastric pathogen *H. pylori*. Finally, 10 proteins have been prioritized following all the criteria. This analysis reduced the cost and time of PVCs identification by excluding proteins with no suitable features for further processing.

The Database of Essential Genes (DEG) [28] was then used to predict essential genes. Results demonstrated that all 10 of the prioritized proteins were essential proteins, thus making them putative vaccine candidates. In the next step, the proteome was screened for virulent proteins, as identification of virulent factors in essential proteins is a key step in the vaccine development process [29]. Essential genes of a pathogen tend to be virulent, substantiating these checks as key factors in the prediction of target proteins to prioritize vaccine candidates [21, 30]. In our case, 267 proteins were found to be virulent proteins among whole proteome of the pathogen.

VFDB [31] results, coinciding with our pipeline-generated results, demonstrated that all prioritized proteins contained virulence factors, concluding that these 10 proteins are potential vaccine targets. Next, proteins were checked for their transmembrane topology. VacSol explored 1254 proteins with less than 2 transmembrane helices, as these proteins are often deemed the best candidates. Having more than one transmembrane helix in a protein makes expression and colonization difficult, and multiple transmembrane helices fail to purify recombinant proteins for vaccine development [21]. HMMTOP version 2.0 [32] was applied to enumerate transmembrane helices with default parameter values. Subsequently, proteins were checked for their functional annotation from UniProt (Table 2) [33]. UniProt characterizes functionality of proteins based on sequence and/or similarity with functionally annotated proteins [23]. Insight into the role of targeted proteins in a system provides a detailed understanding as to how putative targets can be used to reduce pathogen burden and virulence. Prioritized proteins included 3 homologs of *FecA* (HP1400, HP0807, HP0686), *FlaA* (HP0601), *FlaB* (HP0115), *HcpA* (HP0211), *HcpC* (HP1098), and toxin-like outer membrane proteins (HP0289, HP0610, and HP0922). B-cell and T-cell epitopes screened for prioritized candidates along with their features (location, score, no. of MHC I & II binding alleles) have been shown in results file (Additional file 2).

**Table 2:** Functional annotation of prioritized proteins

Protein ID (VacSol)	Bacterial protein	Gene symbol (NCBI)	Molecular weight kDa (ExPASy)	Molecular function (UNIPROT)	Domains (Interpro Scan)	Trans-membrane Helices
3	Iron(III) dicitrate transport protein (FecA)	HP1400	94.827	Receptor activity	TonB-dependent receptor & plug domain	0
285	Flagellin A (FlaA)	HP0601	53.287	Cell motility, Signal transduction and structural molecule activity	Flagellin, Flagellin_D0/D1, Flagellin_hook_IN_motif	0
534	Putative beta-lactamase	HP1098	31.594	Beta-lactamase activity	Sel1-like, TPRlike_ helical_dom, TPR_2	0
825	Iron(III) dicitrate transport protein (FecA)	HP0807	88.946	Receptor activity	TonB-dependent receptor & plug domain	0
837	Flagellin B (FlaB)	HP0115	53.882	Structural molecule activity	Flagellin, Flagellin_D0/D1	0
907	Toxin-like outer membrane protein	HP0289	311.288	Not defined	Autotransport_beta& Vacuolating_cytot oxin_put	1
995	Toxin-like outer membrane protein	HP0922	274.563	Not defined	VacA2 (motif), Autotransporte_beta, PbH1	0
982	Beta-lactamase HcpA	HP0211	27.366	Peptidoglycan, cell wall synthesis	Sel1-like, TPRlike_helical_dom	0
1184	Toxin-like outer membrane protein	HP0610	212.964	Not defined	Vacuolating cytotoxin putative & Autotransporter beta domain	0
1359	Iron(III) dicitrate transport protein (FecA)	HP0686	87.698	Receptor activity	TonB-dependent receptor, betabarrel, plug domain	0

**Fig. 3:**
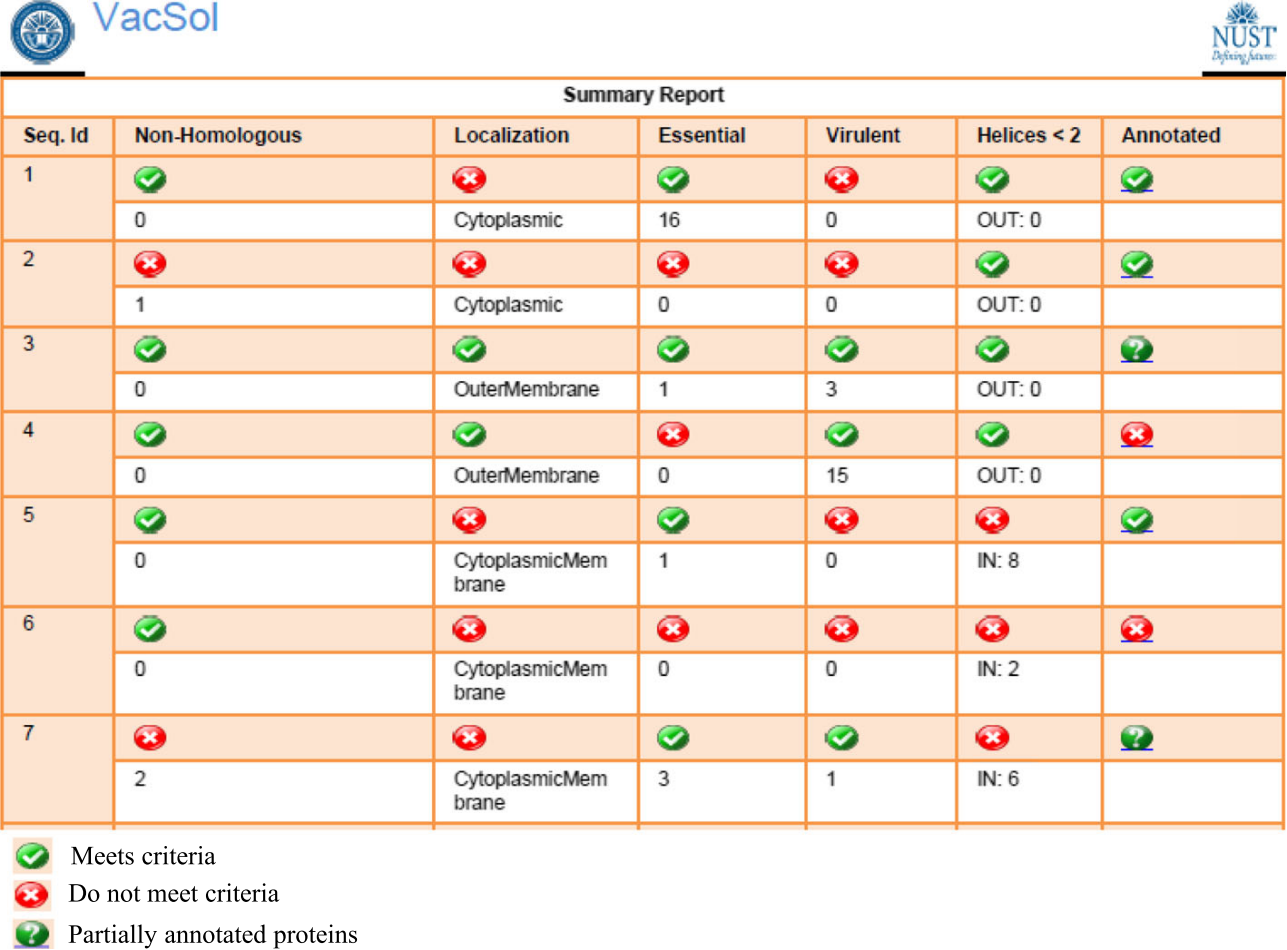
VacSol-generated results. VacSol generated a summary report for the complete *H. pylori* proteome with prioritized proteins. Each protein is assigned a unique VacSol ID

An overview of the results displayed by VacSol are shown in Fig. 3. Each protein sequence was assigned a unique VacSol ID for retrieval, and the overall results for *H. pylori* are provided as Additional file 2. The total duration of these analyses was 90 min, on a machine with 2GB RAM.

## Discussion

The prioritized putative vaccine targets against *H. pylori* 26695 included *FecA* (HP1400), *FecA* (HP0807), *FecA* (HP0686), *FlaA* (HP0601), *FlaB* (HP0115), *HcpA* (HP0211), *HcpC* (HP1098), and toxin-like outer membrane proteins (HP0289, HP0610, and HP0922). Among these target candidates, Iron (III) dicitrate transport protein, *FecA* (HP1400, HP0807, and HP0686), interacts with *TonB*, a protein involved in the virulence process. Previous studies have shown that controlled and mutated *TonB* leads to increased immunization [34]. Indeed, by targeting HP1400, HP0807, and HP0686, *TonB* can be controlled, making these three promising putative vaccine candidates.

Flagelline proteins (*flaA* and *flaB*) are responsible for the pro-inflammation of gastric mucosa that leads to the development of gastric/peptic ulcers, making *flaA* and *flaB* considerable candidates for novel vaccine development [35]. Likewise, Beta-lactamase *HcpA* and *HcpC* are highly pathogenic proteins that are directly involved in different infections caused by *H. pylori* [36]. The *HcpA* protein is also involved in bacterial and eukaryotic host interaction [37]. These protein annotations verify that VacSol limited its screening to the proteins that are biologically relevant putative and therapeutic vaccine candidates.

Previous studies have linked three toxin-like proteins with virulent proteins and vaccine candidates *BabA*, *CagS*, *Cag6, HpaA*, and *VacA* [21]. Indeed, *Cag* proteins are also well-known pathogenic proteins, involved in pathogenic pathways, while the *HcpA* protein has been shown to be involved in bacterial and eukaryotic host interactions [37]. Using our computational approach, we have designed the VacSol pipeline to further the field of vaccinology by reducing time, cost and trial burdens in novel putative vaccine candidate protein identification. Proteins predicted using this pipeline against *H. pylori* strain may serve as promising PVCs against gastric pathogens, as substantiated by previous findings in the literature. Further evaluation of these PVCs can lead to the development of novel and effective vaccines against *H. pylori*.

## Conclusion

VacSol is a new, highly efficient, and user-friendly pipeline established for biological scientists, including those with limited expertise in computational analyses. VacSol has restricted the pool of promising PVCs from the whole bacterial pathogen proteome by automatizing the *in silico* reverse vaccinology approach for the prediction of highly antigenic targeted proteins, via a user controlled step-wise process. This new pipeline is an efficient tool in the contexts of time and computational/experimental costs by eliminating false positive candidates from laboratory evaluation. The modular structure of VacSol improves the prediction process of vaccine candidates with additional potential for future development in this field.

## Availability and requirements

**Project name:** VacSol: An *in silico* pipeline to predict potential therapeutic targets

**Project home page:** https://sourceforge.net/projects/vacsol/files/

**Archived version:** Not available

**Operating system(s):** Linux

**Programming language:** Java

**Other requirements** (Pre Requisite Tools/Languages):

• PSORTb [17]

• NCBI BLAST+ [38]

• Pftools [39]

• Hmmtop [32]

• ABCPred [40]

• ProPred-I [41]

• ProPred [42]

• Java

• Perl

• Bioperl

## Additional files

Additional file 1: Installation Guide. Description: Detailed user guide for installation and usage of VacSol. (PDF 1178 kb)
Additional file 2: Test data results. Description: Detailed results of test data (*H. pylori*) generated by VacSol. (PDF 41330 kb)

## Abbreviations

DEG: Database of Essential Genes
FecA: Iron (III) dicitrate transport protein A
FlaA: Flagelline protein A
FlaB: Flagelline protein B
*H. pylori*: *Helicobacter pylori*
HcpA: Helicobacter cysteine-rich protein A
HcpC: Helicobacter cysteine-rich protein C
PVCs: Potential vaccine candidates
VFDB: Virulence factor database
