# Supplementary material for: VacSol: a high throughput in silico pipeline to predict potential therapeutic targets in prokaryotic pathogens using subtractive reverse vaccinology
Source: BMC Bioinformatics. 2017 Feb 13;18:106. doi: 10.1186/s12859-017-1540-0 (PMC5307925; doi:10.1186/s12859-017-1540-0)
Supplement: Additional file 1: — Installation Guide. Description: Detailed user guide for installation and usage of VacSol. (PDF 1178 kb) [file 12859_2017_1540_MOESM1_ESM.pdf]

Installation Guide

VacSol itself does not require any installation. It is just an executable jar file. But it depends upon other standalone tools. VacSol functionality depends on the installation of various tools that behave as pre-requisite for this pipeline execution. VacSol has tested and analyzed to be fully functional on Ubuntu 12.04.5 64 bit. It is developed in Java which is platform independent. VacSol will work on any operating system, if its pre-requisite tools perform their entire functionality on other operating systems. Current guide is intended for Ubuntu 64 bit desktop environment. User may test it on other environments but should take care of compatibility issues and configurations of all prerequisite tools and environment set.

1.1 Pre Requisite Tools/Languages

- PSORTb
- NCBI Blast+
- Perl
- Bioperl
- Pftools
- Hmmtop
- ABCPred
- ProPred-I
- ProPred
- Java

Above mentioned tools have their own installation requirements. It is mandatory to install these tools correctly and successfully.

Before installing any tool, please open terminal using ALT+CNTRL+T and run following commands.

```
sudo apt-get update
```

```
sudo apt-get install gcc build-essential
```

1.1.1 PSORTb Installation

PSortb Installation instructions are available at following mentioned url http://www.psort.org/downloads/INSTALL.html which is the primary source of PSORTb installation guide.

We have also mentioned below the installation steps for the ease of user, so that he/she can easily install PSORTb.

1.1.1.1 PSORTb Prerequisites

PSORTb need several prerequisites that must be installed for a fully functional version.

1.1.2 Perl (5.6.X or higher)

Install Perl using command or get latest version of Perl from http://www.cpan.org

```
sudo apt-get install perl
```

1.1.3 Bioperl (1.2.X or higher)

Install Bioperl using command or Bioperl can be obtained from http://www.bioperl.org

```
sudo apt-get install bioperl
```

1.1.4 Stand Alone NCBI Blast

Following commands are available for install NCBI Blast or it can be downloaded from NCBI FTP site at ftp://ftp.ncbi.nih.gov/blast/executables/

```
sudo apt-get install blast2
sudo apt-get install ncbi-blast+
```

1.1.5 PFTOOLS

Download pre compiled pftools executable from https://goo.gl/3hqQof.
User is suggested to kindly create a folder named **VacSol** and place/put pftools and othermaterial in that folder. This step is not mandatory but suggested for the organization of installation material.

1.1.5.1 Libpsortb

Download libpsrtb 1.0 from https://goo.gl/MGJbcb or from a primary source http://www.psort.org/download/libpsortb-1.0.tar.gz.

Please also extract and place the above.ar.gz in VacSol folder.

Open the folderVacSol/libpsortb-1.0 in terminal and run following commands in terminal one by one.

```
sudo ./configure
sudo make
sudo make install
sudo ldconfig
```

At any step, if system prompts for any input, please press Enter and use default options.

After executing above mentioned commands, user will see that a folder has created **/usr/local/lib64** with libraries libhmmer, libmodhmm, libsquid and libsvmloc etc

Note: Final libraries must be in a path that the dynamic linker from Perl can find.

Add following paths in .bashrc file

```
export $HOME:/usr/bin

export PATH=$PATH:$HOME:/usr/bin

export PSORT_PFTOOLS=<basePath>/VacSol/pftools

export BLASTDIR=/usr/bin

export LD_LIBRARY_PATH=/usr/local/lib64

export PSORT_ROOT=/usr/local/psort/bin

source ./bashrc
```

User should update the path, if he/she places pftool or blast at any other location. Setthe above environment variables at the end of .bashrc file.

1.1.5.2 bio-tools-psort-all

Download bio-tools-psort-all 3.0.3 from https://goo.gl/5iVtoB or from a primary source http://www.psort.org/download/bio-tools-psort-all.3.0.3.tar.gz

Please also extract and place this in folder VacSol.

Open file VacSol/bio-tools-psort-all/algorithm-hmm/*hmm-binding.cpp* in any text editor and add a line *#include <string.h>* after the line *#include "hmm.h"* at the top of the file (If this line is missed, user should add this line from avoiding to face the exception during installation).

Open the folder VacSol/bio-tools-psort-all in terminal and run following command in terminal

```
perl Makefile.PL
```

During installation, system will require path of blastall, pfscan, libhmmer, libmodhmm, libsquid and libsvmloc liberaries.
Please set the correct path of each library carefully.

Note: If user uses default installations as mentioned above, then blastall will be available at location /usr/bin/. Pfscan will be available in folder VacSol/pftools/ and other libraries will be available in folder /usr/local/lib64/

Download hmmtop 2.1 from https://goo.gl/VeJkdP or from a primary source http://www.enzim.hu/hmmtop/html/download.html

Please also extract and place this in folder VacSol**.**
Navigate to folder VacSol/hmmtop_2.1 in terminal.
Run following commands in terminal or open README file and follow the instructions.

cc hmmtop.c -lm -o hmmtop

After executing above mentioned steps, please add further environment variables in .bashrc file before source ./bashrc line.

```
export PSORT_ROOT=/usr/local/psort/bin

export PSORT_HMMTOP=<basePath>/VacSol/hmmtop_2.1

export HMMTOP_ARCH=<basePath>/VacSol/hmmtop_2.1/hmmtop.arch

export HMMTOP_PSV=<basePath>/VacSol/hmmtop_2.1/hmmtop.psv
```

Navigate back to folder VacSol/bio-tools-psort-all in terminal and execute following commands

sudo make

sudo make test (optional, but recommended)

sudo make install

Run command cp -r psort /usr/local/, this command will copy a psort folder from /home/<username>/Pipeline /bio-tools-psort-all/ to /usr/local/psort

In terminal open a directory using following command

cd /usr/local/psort/conf/analysis/sclblast

and then run a command

sudo ./makedb.sh

After installation please verify that in file /usr/local/psort/bin/psort, the mentioned path at line [my*$root = '/usr/local/psort'*] is correctly set. If you not correctly set then update this path.

Now a standalone PSORTb program is available in $PSORT_ROOT/bin and can be used to test.

1.1.6 OSDDlinux Installation

Install OSDlinux for the installation of ABCPred, Propred and Propred-I.

OSDlinux installation on an existing machine is available at
http://osddlinux.osdd.net/installe.php

There is no need to follow all steps mentioned in installation guide available at
http://osddlinux.osdd.net/installe.php

Please follow only following sections of that guide

- Pre installation setup for all type of machines
- Installation on any Unix based machine
- Post installation for all machines

Required steps are mentioned below for the ease of researcher, user can also easily install it after applying the following steps one by one.

1. Make a directory "/gpsr/" (directory for installing osddlinux, mkdir /gpsr)
2. Now you need to download only following files

    - base.tar.gz (basic or minimum infrastructure)
    - data.tar.gz (BLAST data for creating PSSM profile & similarity search)
    - models.tar.gz (SVM models used for prediction & classification)

3. Uncompressed and detar above files in directory "/gpsr" (e.g., tar -zxvf base.tar.gz)
4. Set the environment paths by using command

    cat /gpsr/gpsr_env.sh >> ~/.bashrc"

    This step will update environment variable HOME as mentioned below.

    $HOME:/gpsr

Review the environment variables in .bashrc file

5. Copy perl of system in /gpsr/local/bin/perl ;directory by using command

```
cp /usr/bin/perl /gpsr/local/bin/
```

6. Change ownership of files in /gpsr ("chown -R <username> /gpsr/*")
7. Change group of all files in /gpsr ("chgrp -R <username> /gpsr/*")
8. Start crontab jobs to excute background jobs ("crontab /gpsr/cronjobs")
9. Restart your computer

1.1.6.1 Modifications

Modify some code files so that VacSol require files in its required format.

Download modified files from https://goo.gl/TNcOUQ and replace existing files with these files.

Replace /gpsr/standalone/abcpred/abcpred.pl with downloaded abcpred.pl file

Similarly replace /gpsr/standalone/propred1/propred1.pl with downloaded propred1.pl file

and replace /gpsr/standalone/propred/propred.pl with downloaded propred.pl file

Now abcpred, propred and propred1 are installed.

Please test abcpred, propred and propred1 individaully to ensure that these tools are installed and configured correctly.

1.1.7 Databases

Download configured database files of DEG, SWISS-Prot, MVIRDB, VFDB etc from https://goo.gl/6aKKUb

1.1.8 VacSol

Download executable VacSol from https://goo.gl/zNYROM and install jre and jdk version 7 or higher.

If there is any jre or jdk version 6, please uninstall it and install version 7 or higher.

1.1.9 Configurations

Download Config files from https://goo.gl/grr11P and place folder Configs in same directory in which VacSol.jar is placed.

1.1.9.1 Log4j Configurations

Create a Temp directory and place its path in Configs /log4j.properties file as mentioned below

```
log = <temp directory path>/log
```

1.1.9.2 VacSol Configurations

Open config.properties and configure paths of DBs and tools in this configuration file as mentioned below.

```
# HSDB Database Path
HSDB=<baseURL>/VacSol/DBs/HSDB/HSDB
# PSortB Bin Directory Path
PSORTB=/usr/local/psort/bin
# DEG Database path
DEGDB=<baseURL>/VacSol/DBs/DEG
# Virulance Factors Database path
VIRFDB=<baseURL>/VacSol/DBs/VIRF
# HMMTOP directory path
HMMTOP=<baseURL>/VacSol/hmmtop_2.1
# Uniprot Database Path
```

```
ANNOT=<baseURL>/VacSol/DBs/ANNOT/uniprot_sprot
# ABCPred for BCEll Epitops Path
ABCPred=/gpsr/standalone/abcpred
# Propred1 for TCEll MHC1 Epitops Path
MHC1=/gpsr/standalone/propred1
# Propred1 for TCEll MHC2 Epitops Path
MHC2=/gpsr/standalone/propred
# XML Template path for using STANDALON mode
TEMPLATE=<baseURL>/VacSol/vacSol.xml
# DEFAULT Submission Method files directory
DEFAULT=<baseURL>/VacSol/Genomes
# Path of TEMP folder
TEMPFOLDER=<baseURL>/VacSol/Temp
# Path for gpsr internal tools TEMP folder
GPSRTEMP=/gpsr/temp
# Directory in which you want to store results
OUTPUT=<crate directory and give its path here>
# Tell system to clear temp directories at the end of processing
CLEAR=false
# Tell system that Internet is available to use, on false value system will not use internet consuming features
INTERNET=true
# Options applicable if internet feature is true
SocketTimeout=30000
# Socket communication time out
ConnectTimeout=30000
# Maximum proteins to process for epitope mapping in one go. Note: 1 Sequences will take around maximum 3 minutes if internet consuming feature is true, it's better to keep this value small not greater than 10 and use multiple passes to save time
```

```
MaxSeqEpitopProcessing = 10
```

Caution: Please do no change path variables, VacSol will recognize only the mentioned path variables.

Table 0.1: Description of Different Pathway Variables.

**PATH VARIABLE**	**PURPOSE**
HSDB	This variable will require path of homo sapiens proteins database which will used by Blaster for homology checking
DEGDB	This variable will require path o;f essential genes database which will used by Blaster for essentiality checking
VIRFDB	This variable will require path of virulent proteins database which will used by Blaster for virulence checking
PSORTB	This variable will require path of PSORTb which will used by Localizer to predict localization.
HMMTOP	This variable will require path of hmmtop which will used by Helicer to calculate transmembrane helices
ANNOT	This variable will require path of database which will used for annotation. Currently UniProt/SwissProt database is configured.
ABCPRED	This variable will require path of ABCPred tool which will be used by Epitoper to calculate B Cell epitopes
MHC1	This variable will require path of Propred1 tool which will be used by Epitoper to calculate MHC-I T Cell epitopes
MHC2	This variable will require path of Propred tool which will be used by Epitoper to calculate MHC-II T Cell epitopes
TEMPLATE	This path variable will require the path of XML Template that is used in case of STANDALONE mode. (For further detail of this XML Template please review Appendix B User Guide)
DEFAULT	This variable require the path of the folder where genome or proteome files are already placed which can be used in DEFAULT input mode.

	(For further detail please review Appendix B User Guide)
TEMPFOLDER	VacSol create some intermediate files during its processing. This variable require the path of such temporary folder. This folder must be created and its path should be configured at this path variable
GPSRTEMP	Tools used by Epitoper also require a temporary folder. Path of such temporary folder must be configured at this path variable. Such temporary folder will be with name temp under gpsr directory (/gpsr/temp).
OUTPUT	VacSol generate result in five different formats and placed in an output directory. This directory must be created and its path should be configured at this path variable
CLEAR	This variable is actually a flag which require true or false value. This indicate VacSol to clear or not temporary directories at the end of processing. Recommended value is true.
INTERNET	This variable is also a flag which require true or false value. This indicate VacSol to use internet (if available) for some internet dependent.
SOCKETTIMEOUT	This variable require socket time-out. Recommended value is 30000
CONNECTTIMEOUT	This variable require connection time-out. Recommended value is 30000
MAXSEQEPITOPPROCESSING	Prioritized proteins will be used for epitope mapping. This variable will limit the number of proteins processing for Epitoper.

1.1.9.3 Additional Configurations

These additional configurations are require to enable DEFAULT input mechanism of VacSol.

Place the default proteome or genome files in a folder configured under path variable DEFAULT.

For example a folder <basePath>/Genome contains files like

- Acinetobacter.fasta
- Campylobacter.fasta
- Bacillus.fasta

And this folder is configured under path variable

DEFAULT=<basePath>/Genome

Open a configuration file proteomes.config and configure the name of these files like

1=Acinetobacter

2=Campylobacter

3=Bacillus

These names will be displayed in dropdown of default input mode and VacSol will automatically accepts the path and name of the file. VacSol will pick that file and process it.

User Guide

Open Terminal and type command

java -jar VacSol.jar [Tool Type] [Debug Level<Optional>] [Number of Cores <Optional>]

Tool Type is first parameter that is composed of two modes (i) GUI and (ii) STANDALONE. GUI option refers to graphical user interface view, whereas STANDALONE option requires input values in XML template and xml file path should be mentioned in configuration file (config.properties) under TEMPLATE variable. Please consult Installation Guide for detail of these path variables.

Debug Level is second parameter and it exhibits values ALL, DEBUG, ERROR, FATAL, INFO, OFF, TRACE or WARN. It is an optional parameter. If user does not select this parameter, then VacSol will use a default value for debugging level as ALL. This parameter informs VacSol to set logging level. Details of logging levels are mentioned in the following table.

Table 1: Description of VacSol Logging Levels.

Level	Description
ALL	All levels including custom levels.
DEBUG	Designates fine-grained informational events that are most useful to debug an application.
ERROR	Designates error events that might still allow the application to continue running.
FATAL	Designates very severe error events that will presumably lead the application to abort.
INFO	Designates informational messages that highlight the progress of the application at coarse-grained level.
OFF	The highest possible rank and is intended to turn off logging.
TRACE	Designates fine-grained informational events than the DEBUG.
WARN	Designates potentially harmful situations.

Note: DEBUG and TRACE logging levels are not supported in current VacSol version.

Number of cores is third parameter that is required to inform how VacSol can utilize as many cores available in host machine. It is also an optional parameter, and by default VacSol will use all available cores of the host machine.

Example: java -jar VacSol.jar GUI ALL 3

1.2 GUI Mode

On opening VacSol in GUI mode it will show a following window.

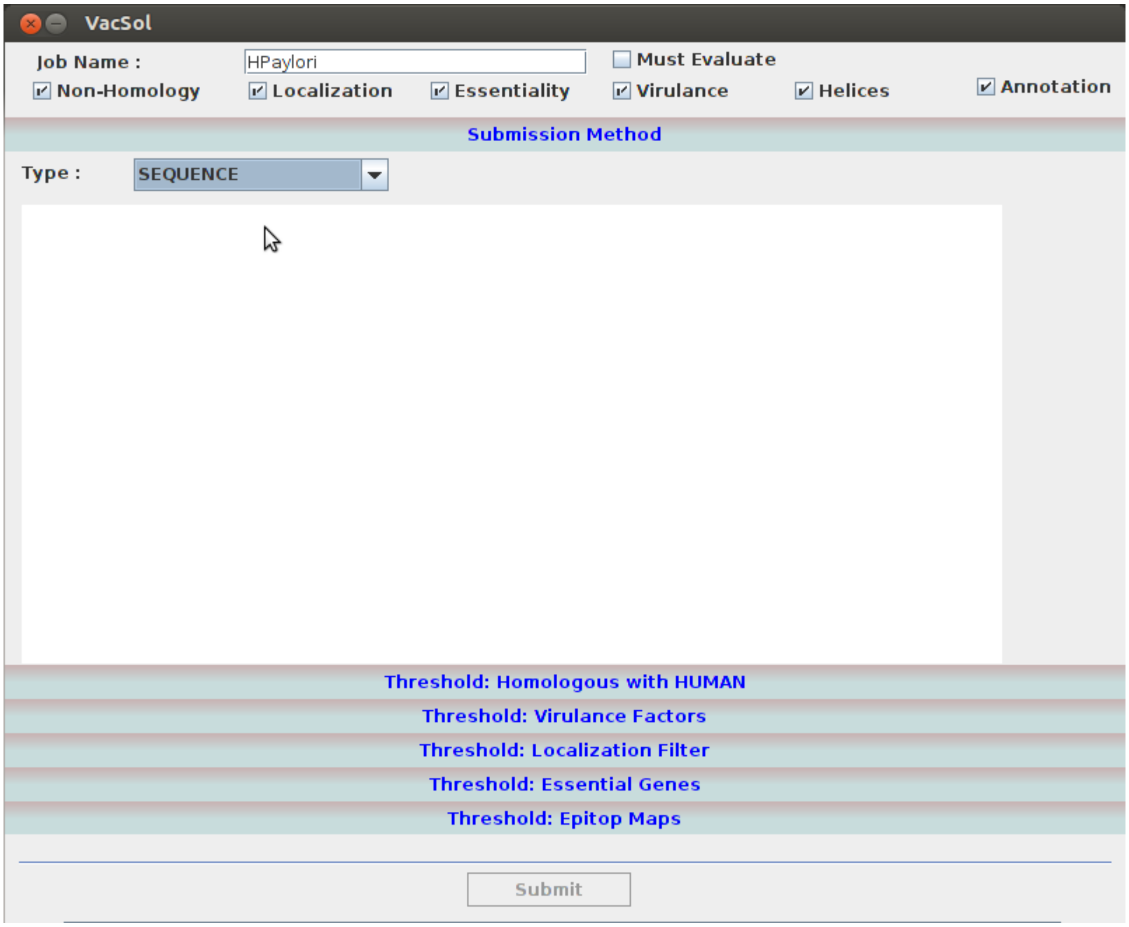

Figure 1: VacSol GUI Mode. It depicts the user friendly graphical user interface.

In top section of window VacSol requires some input in the form of checkboxes.

Non-Homology: This check box indicates that non-host homology checking is required or not.

Localization: This check box indicates that localization prediction is required or not.

Essentiality: This check box indicates that essentiality checking is required or not.

Virulance: This check box indicates that virulence protein identification is required or not.

Helices: This check box indicates transmembrane helices calculation is required or not.

Annotation: This check box indicates that the annotation of protein sequence with UniProt data base is required or not.

These check boxes control the behavior of VacSol, and make it feasible to work as a single tool or as a package of tools. To identify prioritize protein, all these checks must be checked.

Must Evaluate: This check is very important when user wants to use VacSol to identify prioritize protein(s). VacSol works in a flow as mentioned in Fig 3.1 and Fig. 3.5. If you see in Fig 3.5 there are some decision boxes and Must Evaluate check override these decision boxes. If Must Evaluate check is checked then VacSol will must evaluate all steps weather the previous step qualify the criteria or not.

Job Name: A text field which requires name of the job and it will save results in a directory with name provided as Job Name(s).

1.2.1 Submission Method

VacSol pipeline offers four different submission options. Accordion panel bar has title Submission method. This panel contains a drop down list of types through which user can select the required type.

1.2.1.1 SEQUENCE

In Sequence option, a small window will open in which user can paste the protein sequence in plain format and submit it.

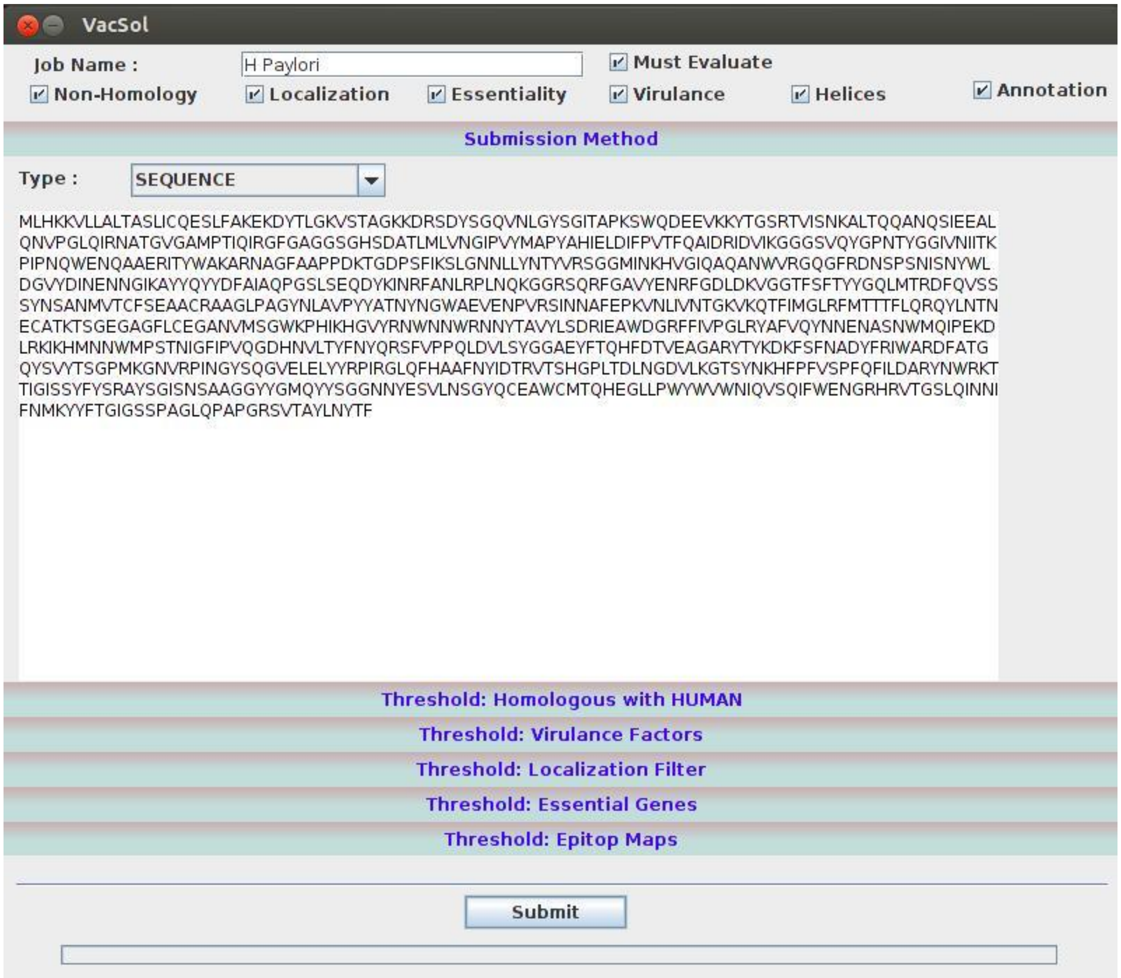

Figure 2: VacSol Input Acceptance Way in Sequence Form. If a user selects a sequence way, then a text box appears to paste the sequence in FASTA format.

1.2.1.2 FILE

This submission method provides option to user of selecting the desired file and submit it to VacSol pipeline. On selection of file, VacSol displays the count of protein sequences in front of Browse button.

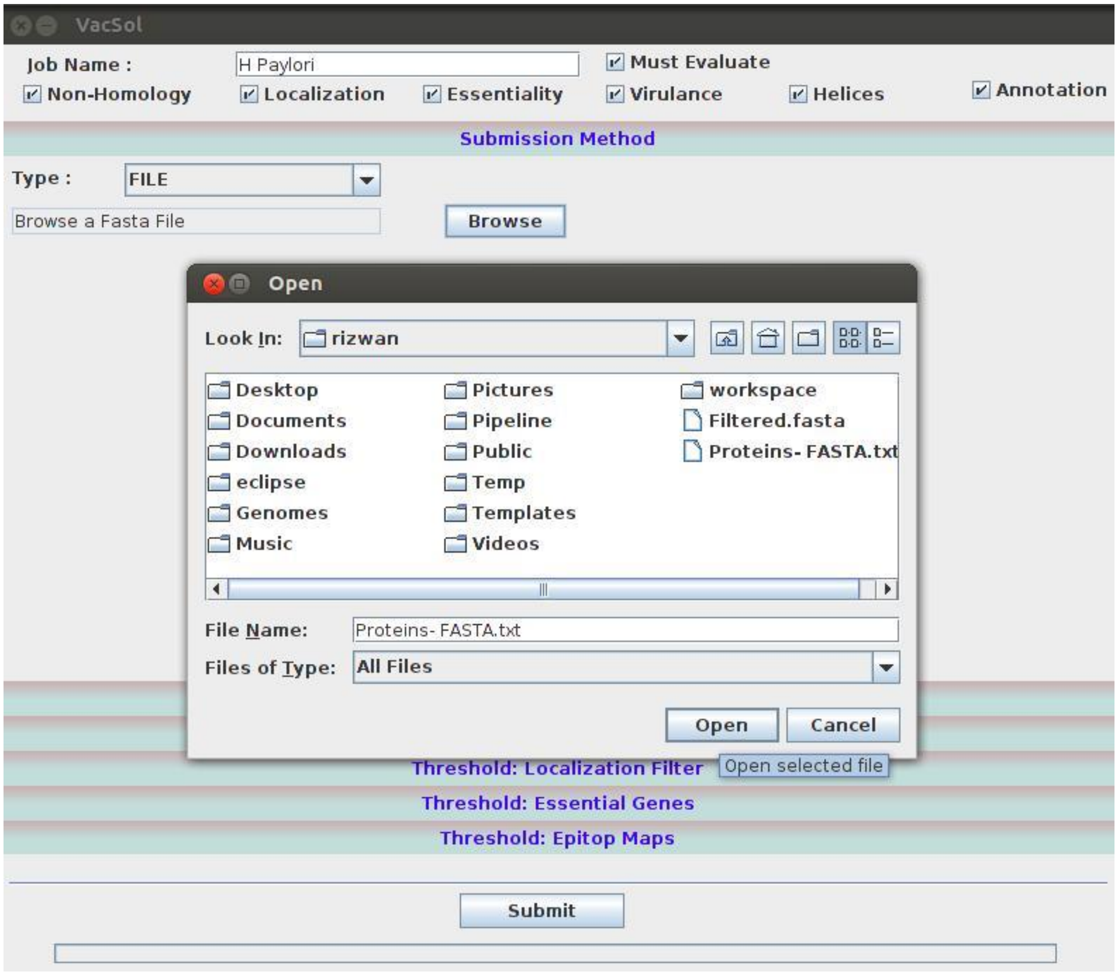

Figure 3: VacSol Input Acceptance Way in File Form. A pop up window will open o select proteome sequence file, if user selects a File form.

1.2.1.3 DEFAULT

This option display another dropdown to select one of the pre-configured default fasta file.

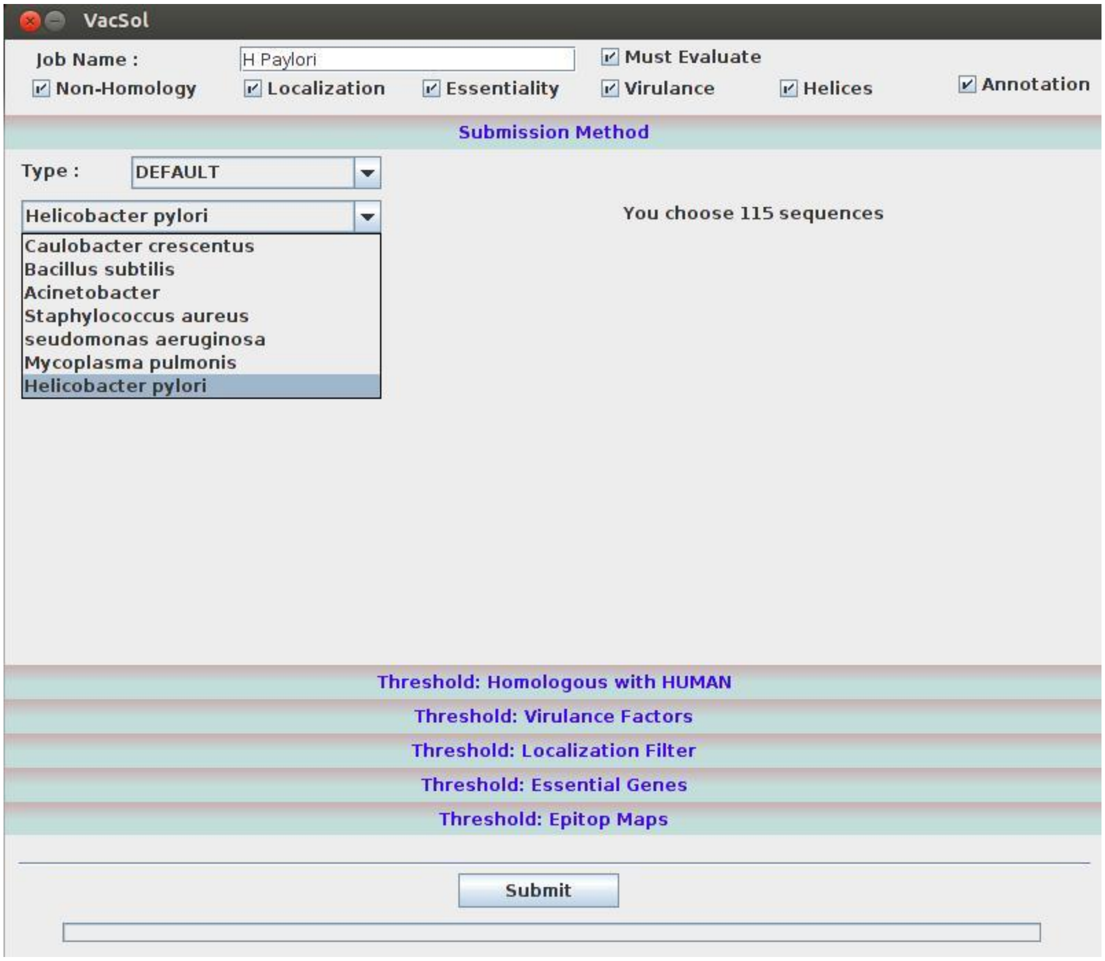

Figure 4: VacSol Input Acceptance Way from Already Available Sequences. On selecting Default option, a drop down will appear to show the bacterial proteomes that have already present in VacSol.

1.2.1.4 ONLINE

This option requires a UniProt protein ID. INTERNET variable must be configured as true and internet should available at host machine, for using this online option. VacSol will download the protein of provided id and execute it based on provided parameters.

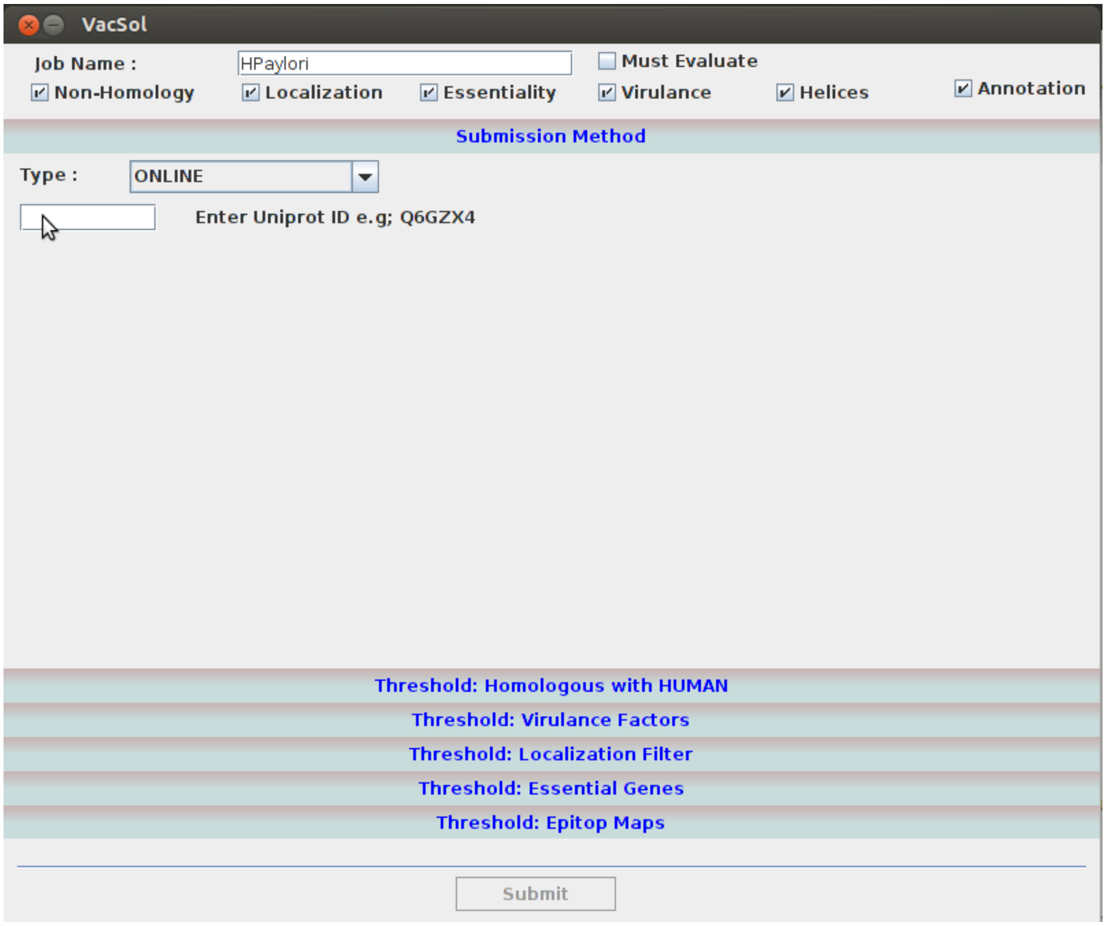

Figure 5: ***VacSol Input Acceptance through Online Retrieving Sequence.*** Online option requires a UniProt protein ID.

1.2.2 Threshold: Homologous with Human

This panel requires the threshold values of evaluation criteria. VacSol evaluates homologous proteins with human based on provided criteria and discards them from results and identify non-homologous proteins.

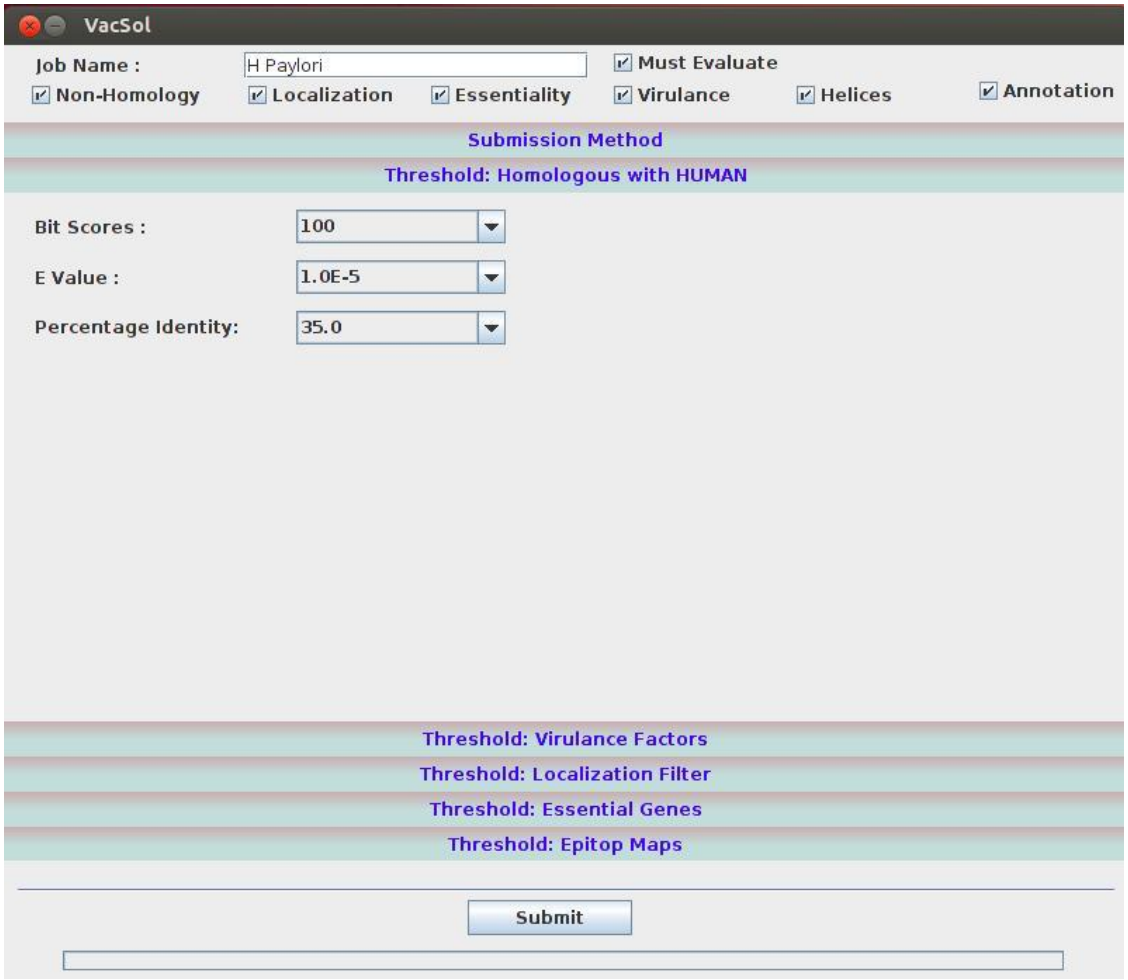

Figure 6: Threshold Representation for Homology Detection. This figure clearly displays the threshold values that have set for determining homology among given bacterial sequences and the human sequences.

1.2.3 Threshold: Virulance Factors

This panel requires the threshold values of virulence factor identification criteria. VacSol evaluates virulence proteins based on given threshold criteria.

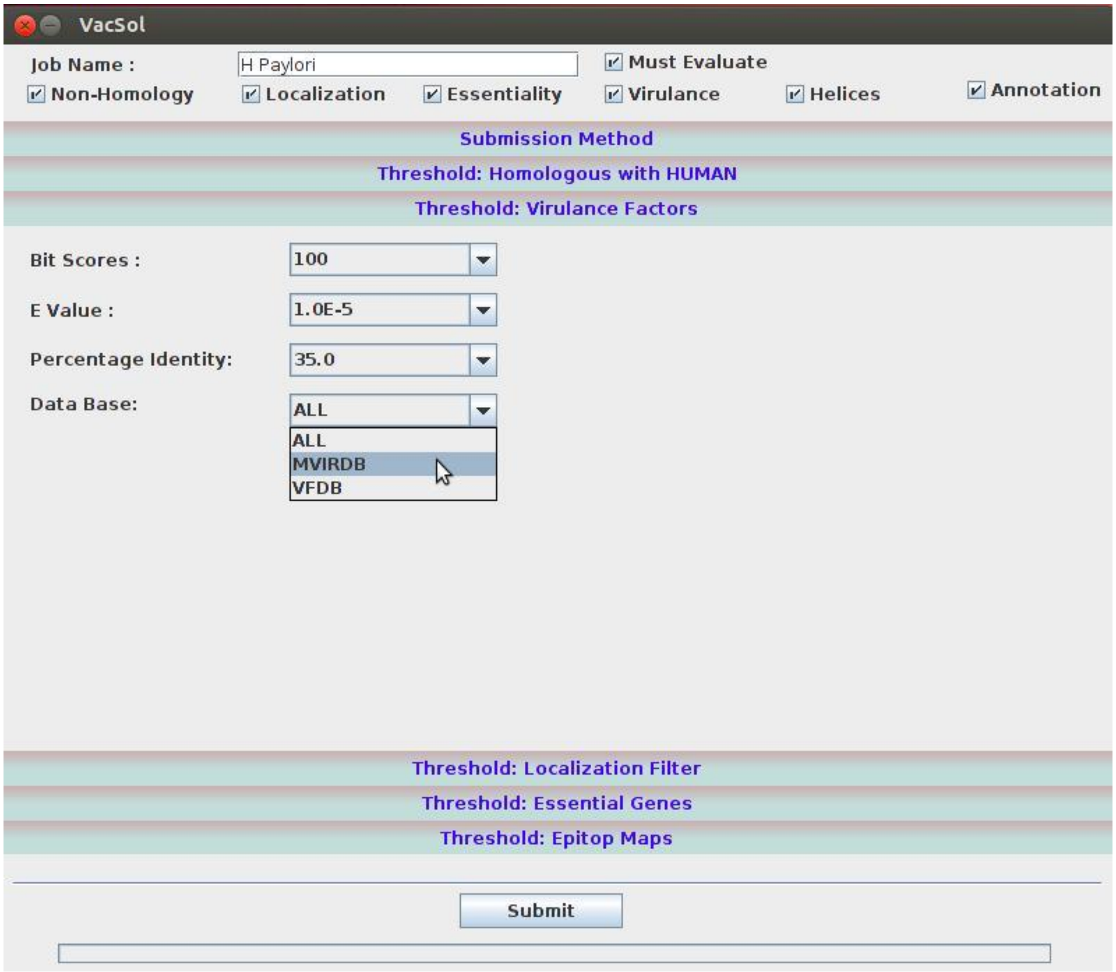

Figure 7: Threshold Representation for Determining Virulence Factors. It displays the threshold values for determining virulence factor by selecting MvirDB, VFDB, or both databases.

1.2.4 Threshold: Localization Filter

This panel requires threshold values for localization identification. VacSol evaluates and identifies proteins which have localizations mentioned in threshold criteria.

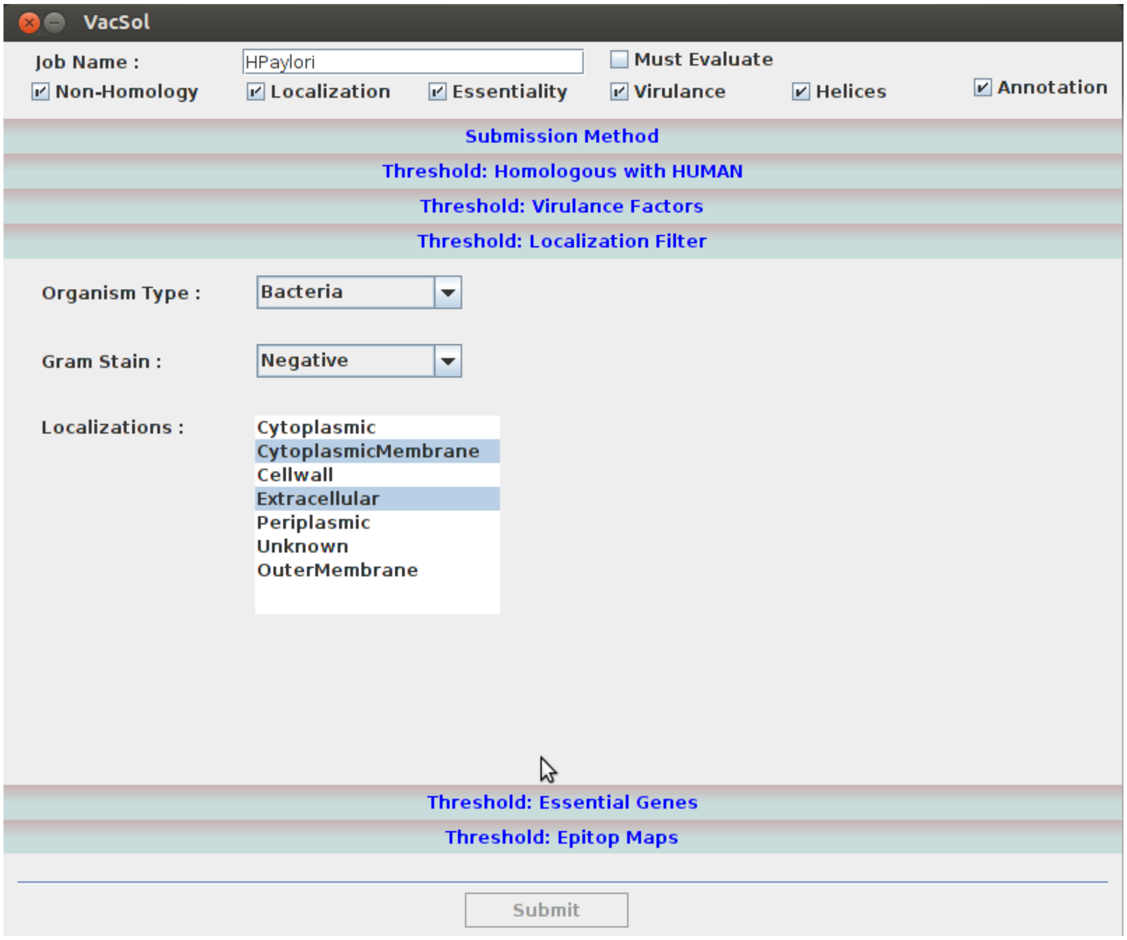

Figure 8: Representation of Threshold Set for Cytoplasmic & Extracellular Localization Filtering. This diagram describes that VacSol is very helpful in determining the protein subcellular locations. It also offers users to select any one of the mentioned locations to determine the protein residential position in cell that would be helpful in determining its function.

1.2.5 Threshold: Essential Genes

This panel requires the threshold values of essential proteins identification criteria. VacSol evaluates essential proteins based on provided threshold criteria.

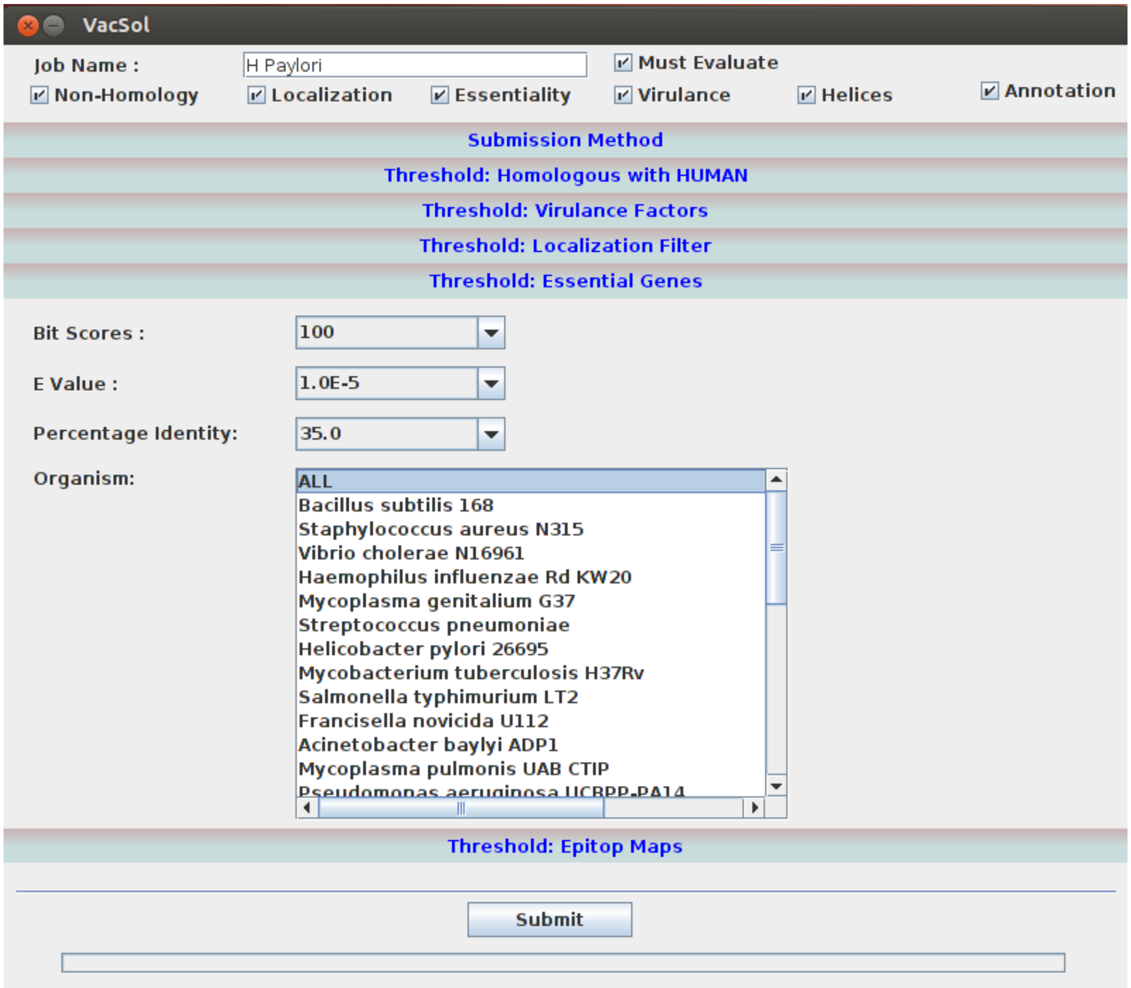

Figure 9: Representation of Threshold Set for Identifying Essential Genes. It also describes threshold values that have been set for essential genes determination. Users can select any bacterial proteome to determine the presence of essential genes in their provided proteome.

1.2.6 Threshold: Epitope Maps

This panel requires the threshold values for epitope maps.

B-Cell Thresold: BCell highest scoring peptides, threshold is a pre-defined numerical value used to make decision.

Windows Length: Number of peptide mers to select. For example 20 mers

T-Cell Threshold: %age of highest scoring peptides.

Top Peptides: Number of top peptides to select.

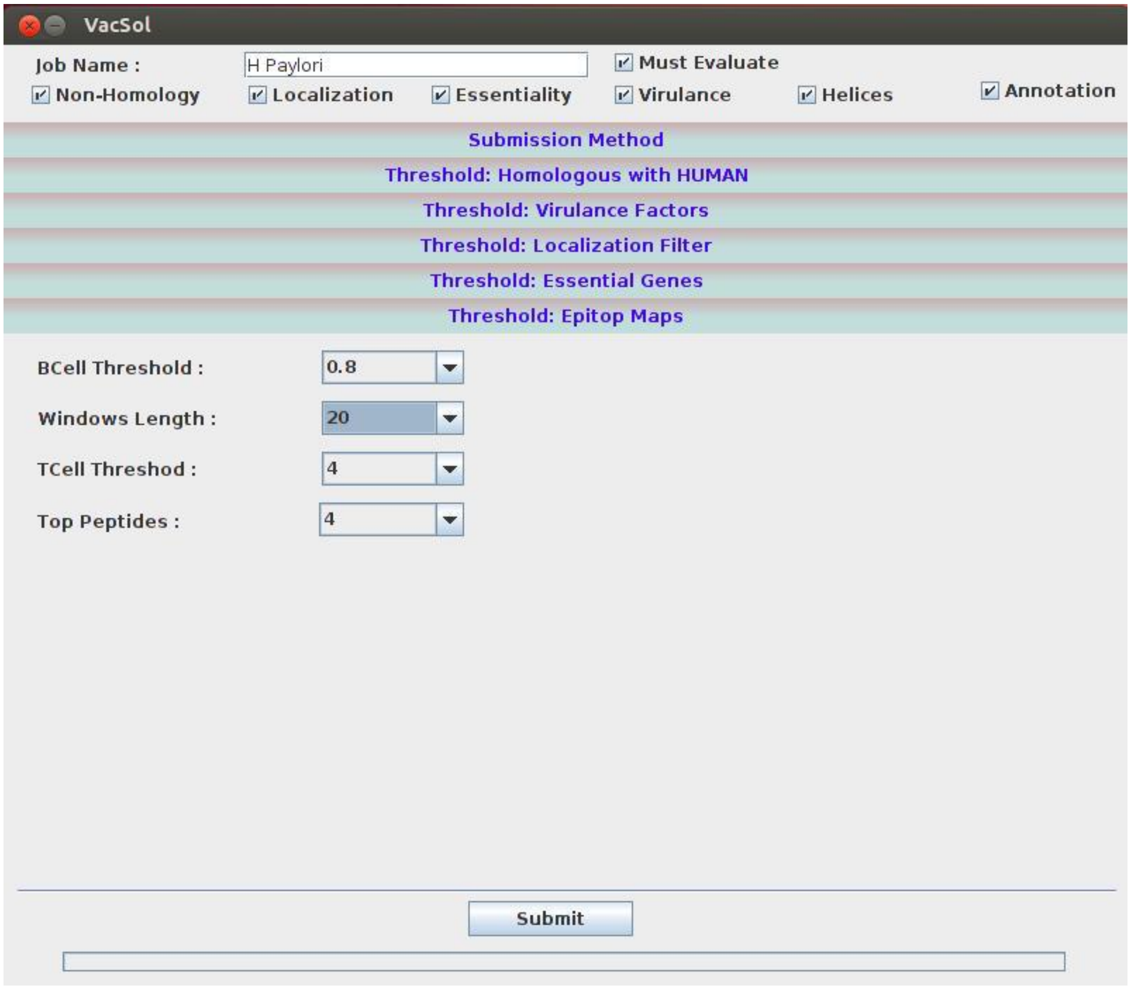

Figure 10: Representation of Threshold Set for Epitope Maps. It displays the threshold values for epitope mapping.

1.3 STANDALONE Mode

Standalone mode is an alternative of GUI mode. In this mode user can provide input values in the form of an XML template. All fields are same and equivalent to GUI mode, but the difference is that in GUI mode user selects values from graphical user interface. While in STANDALONE mode, user will fill values in XML template and set the path of XML template under path variable TEMPLATE.

```xml
<VacSol>
  <JobName>MyTestJob</JobName>
  <MustEvaluate>true</MustEvaluate>
  <isPerformNonHomology>true</isPerformNonHomology>
  <isPerformEssentiality>true</isPerformEssentiality>
  <isPerformVirulance>true</isPerformVirulance>
  <isPerformEpitopMapping>true</isPerformEpitopMapping>
  <isPerformTransmembranceHelices>true</isPerformTransmembranceHelices>
  <SubmissionMethod>
    <Type>SEQUENCE</Type>
    <FilePath>/home/rizwan/File.fasta</FilePath>
    <Sequence>ABCDEFGHIJK</Sequence>
  </SubmissionMethod>
  <Thresholds>
    <Homology>
      <Threshold>
        <BitScore>100</BitScore>
        <eValue>1.0E-5</eValue>
        <PercentageIdentity>35.0</PercentageIdentity>
      </Threshold>
    </Homology>
    <VirulanceFactors>
      <DataBaseType>ALL</DataBaseType>
      <Threshold>
        <BitScore>100</BitScore>
        <eValue>1.0E-5</eValue>
        <PercentageIdentity>35.0</PercentageIdentity>
      </Threshold>
    </VirulanceFactors>
    <Localizations>
      <Localization>Extracellular</Localization>
      <Localization>CytoplasmicMembrane</Localization>
      <OrganismType>Bacteria</OrganismType>
      <GramStain>n</GramStain>
    </Localizations>
    <Essentiality>
      <Organism>DEG</Organism>
      <Organism>DEG1001</Organism>
      <Organism>DEG1013</Organism>
      <Threshold>
        <BitScore>100</BitScore>
        <eValue>1.0E-5</eValue>
        <PercentageIdentity>35.0</PercentageIdentity>
      </Threshold>
    </Essentiality>
    <EpitopMap>
      <BCELL>
        <Thresold>0.5</Thresold>
        <WindowLength>20</WindowLength>
      </BCELL>
      <AntigenThreshold>0.5</AntigenThreshold>
      <TCELL>
        <Thresold>5</Thresold>
        <TopPeptides>5</TopPeptides>
      </TCELL>
    </EpitopMap>
  </Thresholds>
</VacSol>
```


Figure 10: Input Template for Standalone Mode. This figure describes the XML input template for using VacSole as a standalone mode.
