# Supplementary material for: VacSol: a high throughput in silico pipeline to predict potential therapeutic targets in prokaryotic pathogens using subtractive reverse vaccinology
Source: BMC Bioinformatics. 2017 Feb 13;18:106. doi: 10.1186/s12859-017-1540-0 (PMC5307925; doi:10.1186/s12859-017-1540-0)
Supplement: Additional file 2: — Test data results. Description: Detailed results of test data (H. pylori) generated by VacSol. (PDF 41330 kb) [file 12859_2017_1540_MOESM2_ESM.pdf]

VacSol

NUST
Defining futures

Summary Report						
**Seq. Id**	**Non-Homologous**	**Localization**	**Essential**	**Virulent**	**Helices < 2**	**Annotated**
1	✔	✘	✔	✘	✔	✔
	0	Cytoplasmic	16	0	OUT: 0	
2	✘	✘	✘	✘	✔	✔
	1	Cytoplasmic	0	0	OUT: 0	
3	✔	✔	✔	✔	✔	?
	0	OuterMembrane	1	3	OUT: 0	
4	✔	✔	✘	✔	✔	✘
	0	OuterMembrane	0	15	OUT: 0	
5	✔	✘	✔	✘	✘	✔
	0	CytoplasmicMembrane	1	0	IN: 8	
6	✔	✘	✘	✘	✘	✘
	0	CytoplasmicMembrane	0	0	IN: 2	
7	✘	✘	✔	✔	✘	?
	2	CytoplasmicMembrane	3	1	IN: 6	
8	✔	✘	✘	✔	✔	✔

	0	Cytoplasmic	0	11	OUT: 0	
9	✓	✗	✓	✓	✓	✓
	0	Cytoplasmic	1	6	OUT: 0	
10	✓	✓	✗	✓	✓	✓
	0	OuterMembrane	0	48	IN: 1	
11	✓	✗	✗	✓	✓	✓
	0	Unknown	0	4	OUT: 0	
12	✗	✗	✓	✗	✓	✓
	4	Cytoplasmic	3	0	OUT: 0	
13	✓	✗	✗	✗	✓	?
	0	Unknown	0	0	OUT: 0	
14	✓	✗	✗	✗	✓	?
	0	Cytoplasmic	0	0	OUT: 0	
15	✓	✗	✓	✗	✓	✓
	0	Cytoplasmic	23	0	OUT: 0	
17	✓	✗	✗	✗	✓	?
	0	Cytoplasmic	0	0	OUT: 0	
16	✓	✗	✗	✗	✓	?
	0	Cytoplasmic	0	0	OUT: 0	
19	✓	✗	✓	✗	✓	✓

	0	Cytoplasmic	1	0	IN: 0	
18	✓	✗	✗	✓	✓	✗
	0	Cytoplasmic	0	3	IN: 1	
21	✓	✗	✗	✗	✓	✗
	0	Unknown	0	0	IN: 1	
20	✓	✗	✗	✗	✗	?
	0	Cytoplasmic	0	0	IN: 3	
23	✓	✗	✗	✗	✓	✗
	0	CytoplasmicMembrane	0	0	OUT: 1	
22	✓	✗	✗	✓	✗	✓
	0	CytoplasmicMembrane	0	3	OUT: 13	
25	✗	✗	✓	✗	✓	✓
	10	Cytoplasmic	4	0	OUT: 0	
24	✓	✗	✓	✗	✓	?
	0	Unknown	1	0	OUT: 0	
27	✓	✗	✗	✗	✗	✗
	0	Cytoplasmic	0	0	OUT: 2	
26	✓	✗	✓	✗	✓	✓
	0	Unknown	4	0	OUT: 0	

29	✔	✘	✘	✘	✔	✘
	0	Unknown	0	0	OUT: 0	
28	✔	✘	✘	✘	✔	✔
	0	Unknown	0	0	IN: 1	
31	✔	✘	✘	✘	✔	✘
	0	Cytoplasmic	0	0	IN: 1	
30	✔	✘	✔	✘	✔	✘
	0	Unknown	1	0	IN: 1	
34	✔	✘	✘	✘	✔	✔
	0	Unknown	0	0	IN: 0	
35	✔	✘	✔	✘	✘	?
	0	Unknown	1	0	OUT: 2	
32	✔	✘	✔	✘	✔	✘
	0	Unknown	1	0	OUT: 0	
33	✔	✘	✘	✘	✔	?
	0	Cytoplasmic	0	0	IN: 1	
38	✘	✘	✔	✘	✘	✔
	9	Periplasmic	1	0	OUT: 2	
39	✔	✘	✘	✘	✘	✘
	0	CytoplasmicMembrane	0	0	IN: 4	

36	✓	✗	✓	✗	✓	✓
	0	Unknown	1	0	OUT: 0	
37	✓	✗	✓	✗	✗	✓
	0	CytoplasmicMembrane	1	0	OUT: 5	
42	✓	✗	✗	✗	✓	✓
	0	Unknown	0	0	OUT: 0	
43	✓	✗	✓	✗	✓	✓
	0	Unknown	1	0	IN: 1	
40	✓	✗	✗	✗	✓	✗
	0	Unknown	0	0	OUT: 1	
41	✓	✗	✗	✗	✓	✓
	0	Cytoplasmic	0	0	OUT: 0	
46	✓	✗	✗	✓	✓	?
	0	Cytoplasmic	0	3	IN: 1	
47	✓	✗	✗	✗	✓	✓
	0	Unknown	0	0	OUT: 1	
44	✓	✓	✗	✓	✓	?
	0	OuterMembrane	0	4	OUT: 0	
45	✓	✗	✓	✗	✗	✓
	0	CytoplasmicMem	1	0	OUT: 15	

		brane				
51	✓	✗	✓	✗	✓	✓
	0	Cytoplasmic	17	0	IN: 0	
50	✓	✗	✗	✗	✗	✓
	0	Cytoplasmic	0	0	OUT: 2	
49	✓	✗	✗	✗	✓	✓
	0	CytoplasmicMembrane	0	0	IN: 1	
48	✓	✗	✓	✗	✓	✓
	0	Unknown	1	0	OUT: 0	
55	✓	✗	✗	✗	✗	✗
	0	Unknown	0	0	IN: 3	
54	✓	✗	✓	✓	✗	✓
	0	CytoplasmicMembrane	1	1	OUT: 13	
53	✓	✓	✗	✗	✓	?
	0	OuterMembrane	0	0	OUT: 0	
52	✓	✗	✗	✓	✗	✓
	0	Cytoplasmic	0	3	OUT: 2	
59	✓	✗	✗	✗	✓	✓
	0	Cytoplasmic	0	0	OUT: 0	

58	✓	✗	✓	✗	✓	?
	0	Unknown	1	0	OUT: 0	
57	✓	✗	✓	✗	✓	✓
	0	Cytoplasmic	3	0	OUT: 1	
56	✓	✗	✗	✗	✓	✗
	0	Cytoplasmic	0	0	OUT: 0	
63	✓	✗	✓	✗	✓	?
	0	Cytoplasmic	1	0	OUT: 0	
62	✓	✗	✓	✗	✓	?
	0	Cytoplasmic	1	0	IN: 1	
61	✗	✗	✓	✓	✓	✓
	4	Periplasmic	1	4	OUT: 0	
60	✗	✗	✓	✗	✓	✓
	16	Cytoplasmic	3	0	OUT: 0	
68	✓	✗	✗	✗	✓	?
	0	Cytoplasmic	0	0	OUT: 0	
69	✓	✗	✗	✗	✓	?
	0	Cytoplasmic	0	0	OUT: 0	
70	✓	✗	✗	✗	✓	✗
	0	Cytoplasmic	0	0	OUT: 0	

71	✔	✘	✘	✔	✔	✔
	0	Cytoplasmic	0	3	OUT: 1	
64	✔	✘	✔	✘	✔	✔
	0	Cytoplasmic	1	0	OUT: 0	
65	✔	✘	✔	✘	✘	?
	0	CytoplasmicMembrane	5	0	OUT: 4	
66	✔	✘	✔	✘	✔	✔
	0	Cytoplasmic	2	0	IN: 0	
67	✔	✘	✘	✘	✔	✘
	0	Cytoplasmic	0	0	IN: 1	
76	✔	✘	✔	✘	✔	?
	0	Unknown	1	0	OUT: 0	
77	✔	✘	✘	✘	✔	✘
	0	CytoplasmicMembrane	0	0	OUT: 1	
78	✔	✘	✘	✘	✔	?
	0	Unknown	0	0	OUT: 0	
79	✔	✘	✘	✘	✔	?
	0	CytoplasmicMembrane	0	0	IN: 1	
	✔	✘	✔	✔	✔	✔

72	0	Cytoplasmic	3	3	OUT: 0	
73	✓	✗	✗	✗	✓	✗
	0	Cytoplasmic	0	0	OUT: 0	
74	✓	✓	✗	✓	✗	✗
	0	Extracellular	0	15	OUT: 2	
75	✓	✓	✗	✓	✓	?
	0	Extracellular	0	7	OUT: 0	
85	✓	✓	✗	✓	✓	✓
	0	OuterMembrane	0	4	IN: 1	
84	✓	✗	✓	✓	✓	✓
	0	Cytoplasmic	1	2	OUT: 0	
87	✓	✗	✗	✗	✓	✗
	0	Cytoplasmic	0	0	OUT: 0	
86	✓	✓	✗	✗	✓	✓
	0	Extracellular	0	0	OUT: 0	
81	✓	✗	✗	✓	✗	✓
	0	CytoplasmicMembrane	0	3	OUT: 11	
80	✓	✗	✗	✗	✓	?
	0	Unknown	0	0	OUT: 0	
83	✓	✗	✗	✗	✓	✓

	0	Cytoplasmic	0	0	OUT: 0	
82	✓	✗	✗	✗	✓	✓
	0	Cytoplasmic	0	0	OUT: 1	
93	✓	✗	✗	✓	✓	✗
	0	Cytoplasmic	0	3	OUT: 0	
92	✓	✗	✗	✗	✓	✓
	0	Periplasmic	0	0	IN: 1	
95	✓	✗	✗	✗	✓	✓
	0	Unknown	0	0	OUT: 0	
94	✓	✗	✗	✓	✓	✓
	0	Unknown	0	1	IN: 1	
89	✓	✗	✓	✗	✗	✗
	0	Unknown	1	0	OUT: 4	
88	✓	✗	✗	✗	✓	✗
	0	Cytoplasmic	0	0	OUT: 0	
91	✓	✗	✗	✓	✓	?
	0	Cytoplasmic	0	4	IN: 0	
90	✓	✗	✓	✗	✓	✓
	0	Cytoplasmic	1	0	IN: 0	
102	✓	✗	✓	✗	✓	✓

	0	Cytoplasmic	2	0	IN: 1	
103	✔	✘	✘	✘	✔	?
	0	Unknown	0	0	IN: 1	
100	✔	✘	✘	✔	✔	✘
	0	Unknown	0	8	IN: 1	
101	✔	✘	✔	✘	✔	✔
	0	Cytoplasmic	6	0	OUT: 0	
98	✔	✘	✘	✘	✔	✘
	0	Cytoplasmic	0	0	OUT: 0	
99	✘	✘	✔	✔	✔	✔
	1	Cytoplasmic	29	3	OUT: 0	
96	✔	✘	✘	✘	✔	✘
	0	Cytoplasmic	0	0	OUT: 0	
97	✔	✘	✔	✘	✔	✔
	0	Cytoplasmic	11	0	OUT: 0	
110	✔	✘	✘	✘	✘	✔
	0	Unknown	0	0	OUT: 2	
111	✔	✘	✘	✘	✘	✔
	0	Unknown	0	0	OUT: 2	
108	✔	✘	✘	✘	✔	?

	0	Unknown	0	0	OUT: 0	
109	✘	✘	✔	✘	✘	✔
	6	Cytoplasmic	16	0	OUT: 2	
106	✔	✘	✔	✘	✘	✘
	0	Unknown	1	0	IN: 5	
107	✘	✘	✔	✔	✔	✔
	3	Cytoplasmic	2	3	OUT: 0	
104	✔	✘	✘	✘	✘	?
	0	Cytoplasmic	0	0	OUT: 2	
105	✔	✘	✔	✘	✔	✔
	0	Cytoplasmic	8	0	OUT: 0	
119	✔	✘	✘	✘	✔	?
	0	Unknown	0	0	IN: 1	
118	✔	✘	✘	✘	✔	?
	0	Unknown	0	0	OUT: 0	
117	✔	✘	✔	✘	✔	✔
	0	CytoplasmicMembrane	1	0	OUT: 0	
116	✔	✘	✔	✘	✔	?
	0	Cytoplasmic	1	0	OUT: 0	
115	✔	✘	✘	✘	✔	?

	0	Cytoplasmic	0	0	OUT: 1	
114	✔	✘	✘	✘	✔	?
	0	Cytoplasmic	0	0	OUT: 0	
113	✔	✘	✔	✔	✔	✔
	0	CytoplasmicMembrane	1	1	OUT: 1	
112	✔	✔	✘	✘	✔	✔
	0	Extracellular	0	0	IN: 1	
127	✔	✘	✘	✘	✔	✔
	0	Cytoplasmic	0	0	IN: 1	
126	✘	✘	✔	✔	✔	✔
	5	Cytoplasmic	24	3	OUT: 0	
125	✔	✘	✔	✔	✘	✔
	0	CytoplasmicMembrane	12	6	IN: 5	
124	✔	✘	✔	✘	✔	✔
	0	Cytoplasmic	1	0	OUT: 0	
123	✔	✘	✔	✘	✔	✔
	0	Cytoplasmic	16	0	IN: 1	
122	✔	✘	✘	✘	✘	✘
	0	Cytoplasmic	0	0	OUT: 3	

121	✓	✗	✗	✗	✗	?
	0	CytoplasmicMembrane	0	0	IN: 9	
120	✓	✗	✗	✗	✓	✗
	0	CytoplasmicMembrane	0	0	IN: 1	
137	✓	✗	✗	✓	✓	?
	0	Unknown	0	2	OUT: 0	
136	✓	✗	✗	✓	✓	?
	0	CytoplasmicMembrane	0	2	OUT: 1	
139	✓	✗	✓	✗	✓	✓
	0	Cytoplasmic	2	0	OUT: 0	
138	✓	✗	✓	✗	✓	✗
	0	Cytoplasmic	1	0	OUT: 0	
141	✓	✗	✗	✓	✗	?
	0	CytoplasmicMembrane	0	4	OUT: 2	
140	✓	✗	✗	✗	✓	✗
	0	Unknown	0	0	OUT: 0	
143	✓	✗	✗	✗	✗	?
	0	CytoplasmicMem	0	0	IN: 6	

		brane				
142	✓	✗	✗	✗	✓	?
	0	Cytoplasmic	0	0	OUT: 0	
129	✓	✗	✗	✗	✓	✗
	0	Unknown	0	0	OUT: 0	
128	✓	✗	✗	✗	✓	✓
	0	Cytoplasmic	0	0	IN: 0	
131	✓	✗	✗	✗	✓	?
	0	CytoplasmicMembrane	0	0	OUT: 0	
130	✓	✗	✗	✗	✓	✗
	0	Cytoplasmic	0	0	OUT: 0	
133	✓	✓	✗	✓	✓	✗
	0	OuterMembrane	0	15	OUT: 0	
132	✓	✗	✗	✗	✓	?
	0	Unknown	0	0	OUT: 1	
135	✓	✗	✗	✗	✓	✗
	0	Unknown	0	0	IN: 1	
134	✓	✗	✓	✗	✗	✓
	0	CytoplasmicMembrane	2	0	IN: 7	

152	✓	✗	✓	✗	✓	✓
	0	Unknown	1	0	OUT: 0	
153	✓	✗	✗	✗	✗	?
	0	CytoplasmicMembrane	0	0	IN: 12	
154	✗	✗	✓	✗	✓	✓
	9	Cytoplasmic	7	0	OUT: 0	
155	✓	✗	✓	✗	✓	✓
	0	Cytoplasmic	1	0	OUT: 0	
156	✓	✗	✗	✗	✗	✓
	0	Unknown	0	0	IN: 3	
157	✓	✗	✗	✗	✓	✗
	0	Unknown	0	0	IN: 1	
158	✓	✗	✗	✗	✗	✗
	0	Unknown	0	0	OUT: 4	
159	✓	✗	✗	✗	✓	✗
	0	Unknown	0	0	IN: 0	
144	✓	✗	✓	✗	✓	✓
	0	Cytoplasmic	1	0	OUT: 0	
145	✓	✗	✗	✗	✓	?
	0	Unknown	0	0	IN: 1	

146	✓	✗	✗	✗	✓	?
	0	Cytoplasmic	0	0	OUT: 0	
147	✓	✗	✓	✗	✓	?
	0	Cytoplasmic	2	0	OUT: 0	
148	✓	✗	✗	✓	✓	?
	0	Cytoplasmic	0	4	OUT: 0	
149	✓	✗	✗	✗	✓	✓
	0	Cytoplasmic	0	0	OUT: 0	
150	✓	✗	✗	✗	✓	✓
	0	Unknown	0	0	OUT: 0	
151	✓	✗	✗	✗	✓	✗
	0	Cytoplasmic	0	0	OUT: 0	
171	✓	✗	✗	✗	✓	✓
	0	Cytoplasmic	0	0	OUT: 0	
170	✓	✗	✗	✗	✓	✗
	0	Unknown	0	0	OUT: 0	
169	✓	✗	✗	✗	✓	✓
	0	Cytoplasmic	0	0	OUT: 0	
168	✗	✗	✓	✗	✗	✓
	9	Cytoplasmic	3	0	IN: 2	

175						
	0	Cytoplasmic	5	0	OUT: 0	
174						
	0	Unknown	0	0	OUT: 0	
173						
	0	CytoplasmicMembrane	1	0	OUT: 0	
172						
	0	CytoplasmicMembrane	0	0	OUT: 5	
163						
	0	Cytoplasmic	0	0	OUT: 0	
162						
	0	Cytoplasmic	0	0	OUT: 0	
161						
	0	CytoplasmicMembrane	0	66	IN: 2	
160						
	0	Cytoplasmic	1	0	IN: 1	
167						
	0	OuterMembrane	0	3	OUT: 0	

166	0	Unknown	0	0	IN: 1	
165	✓	✗	✓	✗	✓	✗
	0	Unknown	1	0	IN: 1	
164	✗	✗	✗	✓	✓	✓
	1	Cytoplasmic	0	50	OUT: 1	
186	✓	✗	✓	✗	✓	✓
	0	Cytoplasmic	3	0	OUT: 0	
187	✓	✗	✗	✗	✓	?
	0	Cytoplasmic	0	0	OUT: 0	
184	✓	✗	✓	✗	✓	✗
	0	Cytoplasmic	1	0	OUT: 0	
185	✓	✗	✓	✗	✓	?
	0	Cytoplasmic	1	0	OUT: 0	
190	✓	✗	✓	✓	✗	✓
	0	CytoplasmicMembrane	2	1	OUT: 3	
191	✓	✗	✗	✗	✓	✗
	0	Unknown	0	0	OUT: 0	
188	✓	✗	✓	✗	✓	✓
	0	Cytoplasmic	1	0	IN: 0	
189	✓	✗	✓	✗	✓	✗

	0	Unknown	1	0	IN: 1	
178	✓	✗	✗	✗	✓	✓
	0	Cytoplasmic	0	0	OUT: 0	
179	✓	✗	✗	✗	✓	✓
	0	Cytoplasmic	0	0	OUT: 1	
176	✓	✗	✓	✗	✓	✓
	0	CytoplasmicMembrane	2	0	OUT: 0	
177	✓	✗	✗	✗	✓	✗
	0	CytoplasmicMembrane	0	0	IN: 1	
182	✓	✗	✓	✗	✓	✓
	0	Cytoplasmic	4	0	OUT: 0	
183	✓	✗	✗	✗	✓	✗
	0	Unknown	0	0	IN: 1	
180	✓	✗	✗	✓	✗	✓
	0	CytoplasmicMembrane	0	3	IN: 12	
181	✓	✗	✗	✗	✓	✗
	0	Cytoplasmic	0	0	OUT: 0	
205	✓	✗	✗	✗	✓	✓

	0	Cytoplasmic	0	0	OUT: 0	
204	✓	✗	✓	✗	✓	✓
	0	Cytoplasmic	1	0	OUT: 0	
207	✓	✗	✗	✗	✓	✓
	0	Cytoplasmic	0	0	IN: 1	
206	✓	✗	✓	✗	✗	?
	0	CytoplasmicMembrane	6	0	OUT: 14	
201	✗	✗	✓	✗	✗	✓
	1	CytoplasmicMembrane	1	0	IN: 13	
200	✓	✗	✗	✗	✓	✓
	0	CytoplasmicMembrane	0	0	IN: 1	
203	✓	✗	✓	✗	✗	?
	0	CytoplasmicMembrane	1	0	IN: 13	
202	✓	✓	✗	✗	✓	?
	0	Extracellular	0	0	OUT: 0	
197	✓	✗	✗	✗	✓	✓
	0	Cytoplasmic	0	0	IN: 1	
196	✓	✗	✓	✗	✓	✓

	0	Cytoplasmic	1	0	OUT: 0	
199	✔	✘	✘	✘	✔	✔
	0	Cytoplasmic	0	0	OUT: 0	
198	✔	✘	✔	✘	✔	✔
	0	Cytoplasmic	1	0	OUT: 0	
193	✔	✘	✘	✘	✔	✘
	0	Unknown	0	0	OUT: 0	
192	✔	✘	✔	✘	✔	✔
	0	Cytoplasmic	43	0	OUT: 0	
195	✔	✘	✘	✘	✘	✔
	0	CytoplasmicMembrane	0	0	IN: 6	
194	✔	✘	✔	✘	✔	✔
	0	Cytoplasmic	1	0	OUT: 0	
220	✔	✘	✘	✘	✔	✘
	0	Cytoplasmic	0	0	OUT: 0	
221	✔	✘	✔	✘	✘	✘
	0	CytoplasmicMembrane	1	0	IN: 4	
222	✔	✘	✘	✘	✘	?
	0	CytoplasmicMem	0	0	IN: 5	

		brane				
223	✔	✘	✔	✘	✔	✔
	0	Unknown	2	0	OUT: 0	
216	✔	✘	✔	✘	✔	✔
	0	Cytoplasmic	49	0	OUT: 0	
217	✔	✘	✔	✘	✔	✔
	0	Cytoplasmic	7	0	OUT: 1	
218	✔	✘	✔	✘	✔	?
	0	Cytoplasmic	1	0	OUT: 0	
219	✔	✘	✔	✘	✔	✔
	0	Cytoplasmic	21	0	OUT: 0	
212	✔	✘	✘	✘	✘	?
	0	CytoplasmicMembrane	0	0	IN: 2	
213	✔	✘	✔	✔	✔	✔
	0	Cytoplasmic	28	1	OUT: 1	
214	✔	✘	✔	✔	✔	✔
	0	CytoplasmicMembrane	13	3	OUT: 0	
215	✔	✘	✔	✘	✔	✔
	0	Cytoplasmic	3	0	OUT: 0	

208	✓	✗	✓	✓	✗	✓
	0	CytoplasmicMembrane	24	43	IN: 10	
209	✓	✗	✓	✗	✓	?
	0	Cytoplasmic	1	0	OUT: 0	
210	✓	✗	✗	✗	✓	✓
	0	CytoplasmicMembrane	0	0	OUT: 1	
211	✓	✗	✗	✗	✓	✗
	0	Unknown	0	0	OUT: 0	
239	✓	✗	✗	✗	✓	✓
	0	Cytoplasmic	0	0	OUT: 0	
238	✗	✗	✓	✗	✗	✓
	12	Cytoplasmic	6	0	OUT: 2	
237	✓	✗	✗	✗	✓	✗
	0	Unknown	0	0	OUT: 0	
236	✓	✗	✗	✗	✓	✗
	0	Unknown	0	0	IN: 1	
235	✓	✗	✗	✓	✓	✓
	0	Periplasmic	0	18	IN: 1	
234	✓	✗	✗	✗	✓	?

	0	Unknown	0	0	OUT: 0	
233	✓	✗	✓	✗	✓	?
	0	Cytoplasmic	1	0	OUT: 0	
232	✓	✗	✗	✗	✓	✗
	0	Unknown	0	0	OUT: 0	
231	✓	✗	✓	✓	✗	✓
	0	CytoplasmicMembrane	28	5	OUT: 2	
230	✓	✗	✗	✗	✗	✓
	0	Unknown	0	0	OUT: 2	
229	✓	✗	✗	✗	✗	✗
	0	Unknown	0	0	IN: 3	
228	✓	✗	✗	✗	✓	✗
	0	Unknown	0	0	OUT: 0	
227	✓	✗	✓	✗	✓	✗
	0	Unknown	1	0	OUT: 1	
226	✗	✗	✓	✗	✓	✓
	14	Cytoplasmic	23	0	OUT: 0	
225	✓	✗	✗	✗	✓	✗
	0	Unknown	0	0	OUT: 0	
224	✓	✗	✗	✓	✗	?

	0	CytoplasmicMembrane	0	3	OUT: 2	
254	✓	✓	✗	✗	✓	?
	0	OuterMembrane	0	0	OUT: 0	
255	✓	✗	✓	✗	✓	✓
	0	Cytoplasmic	1	0	OUT: 0	
252	✓	✗	✗	✗	✓	✗
	0	Unknown	0	0	OUT: 0	
253	✓	✗	✗	✗	✓	✓
	0	Cytoplasmic	0	0	OUT: 0	
250	✓	✗	✗	✗	✗	✓
	0	Unknown	0	0	OUT: 3	
251	✓	✗	✗	✗	✓	✗
	0	Cytoplasmic	0	0	OUT: 0	
248	✓	✗	✓	✗	✓	✗
	0	Unknown	1	0	OUT: 1	
249	✓	✗	✗	✓	✗	?
	0	Unknown	0	2	IN: 3	
246	✗	✗	✓	✗	✓	✓
	27	Cytoplasmic	6	0	OUT: 1	
247	✓	✗	✗	✗	✓	?

	0	Cytoplasmic	0	0	OUT: 0	
244	✔	✘	✘	✘	✔	✘
	0	Unknown	0	0	OUT: 0	
245	✔	✘	✘	✘	✔	✘
	0	Unknown	0	0	OUT: 0	
242	✔	✘	✘	✘	✔	✔
	0	Cytoplasmic	0	0	OUT: 0	
243	✔	✘	✘	✘	✔	?
	0	Cytoplasmic	0	0	OUT: 0	
240	✔	✘	✔	✘	✘	✘
	0	CytoplasmicMem brane	1	0	OUT: 4	
241	✔	✘	✘	✘	✔	✔
	0	Unknown	0	0	OUT: 0	
275	✔	✘	✘	✘	✔	?
	0	Unknown	0	0	IN: 1	
274	✔	✘	✔	✘	✔	✔
	0	Unknown	1	0	OUT: 0	
273	✔	✘	✘	✘	✔	?
	0	Unknown	0	0	OUT: 0	
272	✔	✘	✔	✘	✔	✔

	0	Cytoplasmic	15	0	OUT: 0	
279	✓	✗	✓	✗	✓	✓
	0	Cytoplasmic	1	0	OUT: 0	
278	✓	✗	✗	✗	✓	✗
	0	Unknown	0	0	OUT: 1	
277	✓	✗	✗	✗	✓	✗
	0	Unknown	0	0	OUT: 0	
276	✓	✗	✓	✗	✓	✓
	0	Unknown	7	0	OUT: 0	
283	✗	✗	✓	✗	✓	✓
	8	Cytoplasmic	7	0	OUT: 0	
282	✓	✗	✓	✗	✗	✓
	0	Cytoplasmic	15	0	OUT: 2	
281	✓	✗	✗	✗	✗	?
	0	CytoplasmicMembrane	0	0	OUT: 7	
280	✓	✗	✗	✗	✓	?
	0	Unknown	0	0	OUT: 0	
287	✓	✗	✗	✓	✗	✓
	0	CytoplasmicMembrane	0	3	IN: 12	

286	✓	✗	✓	✗	✓	?
	0	Unknown	1	0	OUT: 0	
285	✓	✓	✓	✓	✓	✓
	0	Extracellular	1	69	OUT: 0	
284	✓	✗	✓	✗	✓	✓
	0	Unknown	1	0	OUT: 1	
258	✓	✗	✓	✗	✓	?
	0	CytoplasmicMembrane	1	0	IN: 1	
259	✗	✗	✗	✗	✓	✓
	34	Cytoplasmic	0	0	OUT: 0	
256	✓	✗	✗	✗	✓	✓
	0	Cytoplasmic	0	0	OUT: 0	
257	✓	✗	✗	✗	✓	✗
	0	Unknown	0	0	OUT: 0	
262	✓	✗	✗	✗	✗	✓
	0	CytoplasmicMembrane	0	0	OUT: 12	
263	✓	✗	✗	✗	✓	?
	0	Cytoplasmic	0	0	IN: 0	
260	✓	✗	✗	✗	✓	✓

	0	Cytoplasmic	0	0	OUT: 0	
261	✓	✗	✓	✓	✓	✓
	0	Cytoplasmic	6	8	IN: 1	
266	✓	✗	✗	✓	✓	✓
	0	Cytoplasmic	0	3	IN: 1	
267	✓	✗	✓	✗	✓	✓
	0	Cytoplasmic	1	0	OUT: 0	
264	✓	✗	✗	✗	✗	✗
	0	CytoplasmicMembrane	0	0	IN: 3	
265	✓	✓	✗	✓	✓	?
	0	OuterMembrane	0	15	OUT: 0	
270	✓	✗	✗	✗	✓	✓
	0	Cytoplasmic	0	0	OUT: 0	
271	✓	✗	✓	✓	✓	✓
	0	Cytoplasmic	12	3	OUT: 0	
268	✗	✗	✓	✗	✓	✓
	13	Cytoplasmic	21	0	OUT: 0	
269	✓	✗	✓	✓	✓	✓
	0	Cytoplasmic	13	1	OUT: 0	
305	✓	✗	✗	✗	✗	?

	0	CytoplasmicMembrane	0	0	IN: 4	
304	✔	✘	✘	✔	✔	✔
	0	Cytoplasmic	0	4	IN: 0	
307	✔	✘	✘	✘	✔	✔
	0	Periplasmic	0	0	IN: 1	
306	✔	✘	✔	✘	✔	✔
	0	Cytoplasmic	1	0	OUT: 0	
309	✔	✘	✔	✘	✔	✔
	0	CytoplasmicMembrane	3	0	OUT: 0	
308	✔	✘	✔	✘	✔	?
	0	Unknown	1	0	OUT: 0	
311	✔	✘	✘	✘	✔	✔
	0	Cytoplasmic	0	0	OUT: 0	
310	✘	✘	✔	✘	✔	✔
	5	Cytoplasmic	7	0	OUT: 0	
313	✘	✘	✔	✘	✔	✔
	10	Cytoplasmic	22	0	OUT: 0	
312	✔	✘	✔	✘	✘	✔
	0	Cytoplasmic	21	0	OUT: 3	

315	✔	✘	✘	✘	✔	✔
	0	Cytoplasmic	0	0	OUT: 0	
314	✔	✘	✘	✘	✔	✔
	0	Cytoplasmic	0	0	OUT: 0	
317	✔	✘	✔	✘	✔	?
	0	Unknown	1	0	IN: 1	
316	✔	✘	✘	✘	✘	✔
	0	CytoplasmicMembrane	0	0	IN: 4	
319	✔	✘	✘	✔	✔	✔
	0	Cytoplasmic	0	6	OUT: 0	
318	✔	✘	✔	✘	✔	✘
	0	Cytoplasmic	1	0	OUT: 0	
288	✔	✘	✘	✘	✔	✔
	0	Cytoplasmic	0	0	OUT: 0	
289	✔	✘	✔	✘	✘	✔
	0	CytoplasmicMembrane	3	0	IN: 10	
290	✔	✘	✘	✘	✔	✔
	0	Cytoplasmic	0	0	OUT: 0	
291	✔	✘	✔	✘	✔	✘

	0	Unknown	1	0	OUT: 1	
292						
	0	Unknown	0	3	OUT: 3	
293						
	0	Cytoplasmic	0	0	OUT: 0	
294						
	0	Cytoplasmic	0	0	OUT: 0	
295						
	0	CytoplasmicMembrane	1	0	OUT: 11	
296						
	0	Unknown	0	0	OUT: 2	
297						
	0	Cytoplasmic	0	0	OUT: 0	
298						
	0	Cytoplasmic	0	18	OUT: 0	
299						
	0	CytoplasmicMembrane	0	0	IN: 7	
300						
	0	Cytoplasmic	1	0	IN: 1	

301	✓	✗	✗	✗	✓	✓
	0	Unknown	0	0	OUT: 0	
302	✓	✗	✓	✗	✓	✓
	0	Cytoplasmic	1	0	OUT: 0	
303	✓	✗	✓	✗	✓	✓
	0	Cytoplasmic	26	0	IN: 0	
343	✓	✗	✗	✗	✓	?
	0	Unknown	0	0	OUT: 0	
342	✓	✗	✗	✗	✓	✓
	0	Unknown	0	0	OUT: 0	
341	✓	✗	✓	✗	✓	✓
	0	Cytoplasmic	1	0	OUT: 0	
340	✓	✗	✓	✗	✓	✓
	0	Cytoplasmic	3	0	OUT: 0	
339	✓	✗	✓	✗	✓	✓
	0	Cytoplasmic	25	0	OUT: 0	
338	✓	✗	✗	✗	✓	✓
	0	CytoplasmicMem brane	0	0	OUT: 0	
337	✓	✗	✗	✗	✓	✗
	0	CytoplasmicMem	0	0	IN: 1	

		brane				
336	✓	✗	✗	✗	✓	✓
	0	Cytoplasmic	0	0	OUT: 0	
351	✓	✗	✓	✗	✓	✓
	0	Cytoplasmic	1	0	OUT: 0	
350	✓	✗	✗	✗	✓	✗
	0	Unknown	0	0	OUT: 0	
349	✓	✗	✓	✗	✗	✓
	0	CytoplasmicMembrane	1	0	IN: 12	
348	✓	✗	✗	✗	✓	✗
	0	Unknown	0	0	OUT: 1	
347	✓	✗	✗	✗	✓	✓
	0	Periplasmic	0	0	IN: 1	
346	✓	✗	✓	✓	✓	✓
	0	Cytoplasmic	6	155	IN: 0	
345	✓	✗	✗	✗	✗	?
	0	CytoplasmicMembrane	0	0	IN: 4	
344	✓	✗	✓	✗	✓	✓
	0	Cytoplasmic	17	0	OUT: 0	

326	✓	✗	✗	✗	✗	✗
	0	Unknown	0	0	IN: 5	
327	✓	✗	✓	✗	✓	✓
	0	Cytoplasmic	27	0	OUT: 0	
324	✓	✗	✗	✗	✓	✓
	0	Cytoplasmic	0	0	OUT: 0	
325	✓	✗	✓	✗	✓	?
	0	Cytoplasmic	1	0	OUT: 0	
322	✓	✗	✗	✗	✓	✓
	0	Cytoplasmic	0	0	OUT: 1	
323	✓	✗	✗	✗	✓	✗
	0	Unknown	0	0	IN: 1	
320	✓	✗	✗	✓	✓	✓
	0	Cytoplasmic	0	16	OUT: 0	
321	✓	✗	✗	✗	✗	?
	0	CytoplasmicMembrane	0	0	OUT: 13	
334	✓	✗	✗	✗	✓	✓
	0	Unknown	0	0	OUT: 0	
335	✓	✗	✓	✓	✓	✓
	0	Cytoplasmic	13	3	OUT: 0	

332	✔	✘	✔	✔	✔	✔
	0	Cytoplasmic	16	1	IN: 0	
333	✘	✘	✔	✘	✔	✔
	10	Cytoplasmic	18	0	OUT: 0	
330	✔	✘	✘	✘	✔	✔
	0	Unknown	0	0	OUT: 0	
331	✔	✔	✘	✘	✔	✘
	0	Extracellular	0	0	OUT: 0	
328	✔	✘	✘	✘	✔	✔
	0	Cytoplasmic	0	0	OUT: 0	
329	✔	✘	✘	✘	✔	✔
	0	Cytoplasmic	0	0	OUT: 0	
373	✔	✘	✔	✘	✔	✔
	0	Cytoplasmic	3	0	IN: 0	
372	✔	✘	✘	✘	✔	✘
	0	Unknown	0	0	OUT: 0	
375	✔	✘	✔	✘	✘	✔
	0	Cytoplasmic	27	0	IN: 2	
374	✔	✘	✔	✘	✔	✔
	0	Cytoplasmic	9	0	OUT: 0	

369	✔	✘	✔	✔	✔	✔
	0	Cytoplasmic	1	1	IN: 1	
368	✔	✘	✘	✘	✔	✔
	0	Cytoplasmic	0	0	OUT: 0	
371	✔	✘	✘	✘	✔	?
	0	Unknown	0	0	OUT: 0	
370	✔	✘	✔	✘	✔	?
	0	Cytoplasmic	1	0	OUT: 0	
381	✔	✘	✔	✔	✔	✔
	0	Unknown	1	6	IN: 0	
380	✔	✘	✘	✘	✔	✔
	0	CytoplasmicMembrane	0	0	OUT: 0	
383	✔	✘	✔	✔	✘	✔
	0	CytoplasmicMembrane	1	2	OUT: 7	
382	✘	✘	✔	✘	✔	✔
	5	Cytoplasmic	4	0	OUT: 0	
377	✔	✘	✘	✘	✔	✘
	0	Unknown	0	0	IN: 1	
376	✔	✘	✔	✘	✔	✔

	0	Cytoplasmic	1	0	IN: 0	
379	✔	✘	✔	✘	✘	✔
	0	CytoplasmicMembrane	1	0	IN: 5	
378	✔	✘	✔	✔	✔	✔
	0	Cytoplasmic	2	102	OUT: 0	
356	✔	✘	✘	✔	✔	✔
	0	Cytoplasmic	0	9	OUT: 0	
357	✔	✘	✔	✘	✔	✔
	0	Cytoplasmic	1	0	OUT: 0	
358	✔	✘	✘	✔	✔	?
	0	Unknown	0	15	OUT: 0	
359	✔	✘	✘	✘	✔	?
	0	Cytoplasmic	0	0	OUT: 0	
352	✔	✘	✔	✘	✔	✘
	0	CytoplasmicMembrane	1	0	OUT: 1	
353	✔	✘	✔	✔	✔	✔
	0	CytoplasmicMembrane	2	1	OUT: 0	
354	✔	✘	✘	✘	✘	✘

	0	Unknown	0	0	IN: 5	
355	✓	✗	✓	✗	✓	✓
	0	Cytoplasmic	8	0	OUT: 0	
364	✓	✗	✓	✗	✓	✓
	0	Cytoplasmic	1	0	IN: 1	
365	✓	✗	✓	✗	✗	?
	0	Cytoplasmic	1	0	OUT: 2	
366	✓	✗	✗	✗	✓	✗
	0	Unknown	0	0	OUT: 0	
367	✓	✗	✓	✗	✓	✓
	0	Cytoplasmic	17	0	OUT: 0	
360	✓	✗	✗	✗	✓	✓
	0	Cytoplasmic	0	0	OUT: 0	
361	✓	✗	✗	✗	✓	?
	0	Unknown	0	0	OUT: 0	
362	✗	✗	✓	✗	✓	✓
	11	Cytoplasmic	34	0	OUT: 0	
363	✓	✗	✗	✗	✓	?
	0	Cytoplasmic	0	0	IN: 1	
410	✗	✗	✓	✗	✗	✓

	171	CytoplasmicMembrane	22	0	IN: 3	
411	✓	✗	✗	✗	✓	✓
	0	Cytoplasmic	0	0	IN: 1	
408	✓	✗	✗	✗	✓	?
	0	Unknown	0	0	IN: 1	
409	✓	✗	✗	✗	✓	?
	0	Cytoplasmic	0	0	OUT: 0	
414	✓	✗	✗	✗	✓	?
	0	Cytoplasmic	0	0	IN: 1	
415	✓	✗	✗	✓	✗	?
	0	CytoplasmicMembrane	0	5	IN: 2	
412	✗	✗	✓	✗	✓	✓
	23	Cytoplasmic	25	0	OUT: 0	
413	✓	✗	✗	✗	✓	✓
	0	Cytoplasmic	0	0	OUT: 0	
402	✓	✗	✗	✗	✓	✗
	0	Unknown	0	0	OUT: 0	
403	✓	✗	✗	✗	✗	✓
	0	CytoplasmicMem	0	0	OUT: 7	

		brane				
400	✓	✗	✓	✓	✓	✓
	0	Cytoplasmic	12	4	OUT: 0	
401	✓	✗	✗	✗	✓	✓
	0	Cytoplasmic	0	0	OUT: 0	
406	✓	✗	✗	✗	✓	?
	0	CytoplasmicMem brane	0	0	OUT: 0	
407	✓	✗	✗	✗	✓	✗
	0	Unknown	0	0	OUT: 0	
404	✓	✗	✗	✗	✗	✓
	0	Unknown	0	0	OUT: 2	
405	✓	✗	✓	✗	✓	?
	0	Unknown	1	0	OUT: 0	
395	✓	✗	✗	✗	✗	✓
	0	Periplasmic	0	0	OUT: 2	
394	✓	✗	✗	✗	✓	?
	0	Cytoplasmic	0	0	OUT: 0	
393	✓	✗	✗	✓	✓	✓
	0	Unknown	0	1	OUT: 0	
392	✓	✗	✗	✗	✗	?

	0	Cytoplasmic	0	0	OUT: 2	
399	✔	✘	✘	✘	✔	?
	0	Cytoplasmic	0	0	IN: 1	
398	✔	✘	✘	✘	✔	?
	0	Cytoplasmic	0	0	OUT: 0	
397	✔	✘	✘	✘	✔	?
	0	Unknown	0	0	OUT: 0	
396	✔	✔	✔	✘	✔	?
	0	OuterMembrane	1	0	IN: 1	
387	✔	✘	✘	✘	✔	?
	0	Cytoplasmic	0	0	OUT: 0	
386	✔	✘	✔	✘	✔	✔
	0	Cytoplasmic	1	0	OUT: 0	
385	✔	✘	✘	✘	✘	?
	0	CytoplasmicMembrane	0	0	OUT: 13	
384	✘	✘	✔	✘	✔	✔
	11	Cytoplasmic	1	0	OUT: 0	
391	✔	✘	✔	✔	✘	✔
	0	CytoplasmicMembrane	2	1	IN: 5	

390	✔	✖	✖	✖	✔	?
	0	Cytoplasmic	0	0	OUT: 0	
389	✔	✖	✖	✖	✖	?
	0	CytoplasmicMembrane	0	0	IN: 2	
388	✔	✖	✔	✖	✖	✔
	0	Cytoplasmic	1	0	OUT: 2	
440	✔	✖	✖	✔	✔	✔
	0	Unknown	0	3	IN: 1	
441	✖	✖	✔	✖	✖	✔
	14	CytoplasmicMembrane	1	0	IN: 13	
442	✔	✖	✖	✖	✔	?
	0	Cytoplasmic	0	0	OUT: 0	
443	✔	✖	✔	✔	✔	✔
	0	Cytoplasmic	28	81	OUT: 0	
444	✖	✖	✔	✖	✔	✔
	1	Cytoplasmic	26	0	OUT: 0	
445	✔	✖	✖	✖	✖	✔
	0	CytoplasmicMembrane	0	0	OUT: 11	

446	0	Cytoplasmic	0	0	IN: 1	
447	✓	✗	✓	✗	✓	✓
	0	Cytoplasmic	6	0	OUT: 0	
432	✓	✗	✗	✗	✓	✗
	0	Unknown	0	0	OUT: 1	
433	✗	✗	✓	✗	✓	✓
	4	Cytoplasmic	2	0	OUT: 0	
434	✓	✗	✗	✗	✓	?
	0	Unknown	0	0	OUT: 0	
435	✓	✗	✗	✗	✓	✗
	0	Unknown	0	0	IN: 1	
436	✓	✗	✗	✗	✓	✗
	0	Cytoplasmic	0	0	OUT: 0	
437	✗	✗	✓	✗	✗	✓
	250	CytoplasmicMembrane	7	0	OUT: 18	
438	✓	✗	✓	✗	✓	✓
	0	Cytoplasmic	5	0	OUT: 0	
439	✓	✗	✗	✗	✓	?
	0	Unknown	0	0	OUT: 0	
425	✓	✗	✓	✓	✓	✓

	0	Cytoplasmic	17	24	OUT: 0	
424	✔	✘	✘	✘	✔	?
	0	Cytoplasmic	0	0	OUT: 0	
427	✔	✘	✘	✘	✔	?
	0	Cytoplasmic	0	0	IN: 0	
426	✔	✔	✘	✔	✔	✔
	0	Extracellular	0	4	OUT: 0	
429	✔	✘	✘	✘	✔	✘
	0	Unknown	0	0	OUT: 0	
428	✔	✘	✔	✘	✔	?
	0	Unknown	1	0	OUT: 0	
431	✔	✘	✔	✘	✔	✔
	0	Cytoplasmic	2	0	OUT: 0	
430	✔	✘	✘	✘	✔	✘
	0	Cytoplasmic	0	0	OUT: 0	
417	✔	✘	✔	✘	✔	?
	0	Cytoplasmic	1	0	OUT: 0	
416	✔	✘	✘	✘	✔	✘
	0	Cytoplasmic	0	0	OUT: 0	
419	✔	✔	✔	✘	✔	?

	0	OuterMembrane	1	0	OUT: 0	
418	✔	✘	✔	✘	✔	?
	0	Unknown	1	0	OUT: 0	
421	✔	✘	✔	✘	✔	?
	0	Unknown	1	0	OUT: 0	
420	✔	✘	✘	✘	✔	?
	0	Cytoplasmic	0	0	IN: 1	
423	✔	✘	✘	✘	✔	✔
	0	Cytoplasmic	0	0	OUT: 0	
422	✔	✘	✔	✘	✔	✔
	0	Cytoplasmic	4	0	OUT: 0	
478	✔	✘	✘	✘	✘	✘
	0	CytoplasmicMem brane	0	0	IN: 2	
479	✔	✘	✘	✘	✔	✔
	0	Cytoplasmic	0	0	OUT: 0	
476	✔	✘	✘	✘	✔	✔
	0	Cytoplasmic	0	0	OUT: 0	
477	✔	✘	✘	✘	✔	✔
	0	Cytoplasmic	0	0	IN: 1	
474	✔	✘	✔	✘	✔	?

	0	Cytoplasmic	2	0	OUT: 0	
475	✓	✗	✗	✗	✓	?
	0	CytoplasmicMembrane	0	0	IN: 1	
472	✓	✗	✗	✗	✗	✓
	0	CytoplasmicMembrane	0	0	OUT: 14	
473	✓	✗	✗	✓	✓	✓
	0	Cytoplasmic	0	3	OUT: 0	
470	✓	✗	✓	✗	✗	✓
	0	Unknown	1	0	IN: 3	
471	✓	✗	✗	✗	✗	✗
	0	Cytoplasmic	0	0	OUT: 2	
468	✓	✗	✗	✗	✓	✓
	0	Cytoplasmic	0	0	OUT: 1	
469	✓	✗	✓	✗	✓	?
	0	Cytoplasmic	1	0	OUT: 1	
466	✓	✓	✗	✗	✓	?
	0	OuterMembrane	0	0	OUT: 0	
467	✓	✗	✗	✗	✗	?
	0	Unknown	0	0	IN: 3	

464	✔	✘	✘	✘	✘	✔
	0	CytoplasmicMembrane	0	0	IN: 6	
465	✔	✘	✘	✔	✘	✘
	0	CytoplasmicMembrane	0	3	IN: 3	
463	✔	✘	✔	✘	✔	✔
	0	Cytoplasmic	2	0	OUT: 0	
462	✔	✘	✘	✘	✔	✔
	0	Unknown	0	0	OUT: 0	
461	✔	✘	✔	✘	✔	?
	0	CytoplasmicMembrane	1	0	IN: 1	
460	✔	✘	✘	✘	✘	?
	0	Unknown	0	0	IN: 3	
459	✔	✘	✔	✘	✘	✔
	0	CytoplasmicMembrane	20	0	OUT: 10	
458	✔	✘	✔	✘	✔	✔
	0	Cytoplasmic	24	0	IN: 0	
457	✔	✘	✘	✘	✔	✘
	0	Unknown	0	0	IN: 1	

456	✓	✗	✗	✗	✓	✓
	0	Unknown	0	0	OUT: 0	
455	✗	✗	✓	✓	✓	✓
	16	Cytoplasmic	23	2	IN: 1	
454	✗	✗	✓	✗	✓	✓
	30	CytoplasmicMembrane	4	0	OUT: 0	
453	✗	✗	✓	✓	✓	✓
	126	Cytoplasmic	11	1	OUT: 0	
452	✗	✗	✓	✗	✓	✓
	7	Cytoplasmic	3	0	IN: 1	
451	✓	✗	✗	✗	✓	✓
	0	Cytoplasmic	0	0	OUT: 0	
450	✓	✗	✗	✗	✓	✓
	0	Cytoplasmic	0	0	OUT: 0	
449	✗	✗	✓	✗	✓	✓
	6	Cytoplasmic	24	0	OUT: 0	
448	✓	✗	✗	✗	✓	✓
	0	Unknown	0	0	OUT: 0	
508	✓	✗	✓	✓	✓	✓
	0	CytoplasmicMem	14	9	OUT: 0	

		brane				
509	✓	✗	✓	✗	✓	?
	0	Cytoplasmic	1	0	IN: 1	
510	✓	✗	✗	✗	✗	?
	0	CytoplasmicMem brane	0	0	IN: 7	
511	✓	✗	✗	✗	✓	?
	0	Unknown	0	0	OUT: 0	
504	✓	✗	✓	✓	✓	✓
	0	Cytoplasmic	1	1	OUT: 0	
505	✗	✗	✓	✓	✗	✓
	69	CytoplasmicMem brane	10	33	IN: 5	
506	✓	✗	✗	✗	✓	✓
	0	Cytoplasmic	0	0	OUT: 1	
507	✓	✗	✗	✓	✓	?
	0	Cytoplasmic	0	4	IN: 1	
500	✓	✗	✗	✗	✓	✓
	0	Unknown	0	0	OUT: 0	
501	✓	✗	✗	✗	✓	✓
	0	Unknown	0	0	IN: 1	

502	✓	✗	✓	✗	✗	✓
	0	CytoplasmicMembrane	1	0	IN: 6	
503	✓	✗	✗	✗	✓	✓
	0	Cytoplasmic	0	0	OUT: 0	
496	✓	✗	✓	✓	✓	✓
	0	Cytoplasmic	5	3	OUT: 0	
497	✓	✗	✗	✗	✓	✓
	0	Unknown	0	0	OUT: 0	
498	✓	✗	✓	✓	✓	✓
	0	Cytoplasmic	1	1	OUT: 0	
499	✓	✗	✓	✓	✓	✓
	0	CytoplasmicMembrane	22	13	OUT: 0	
493	✓	✗	✓	✗	✓	✗
	0	Unknown	1	0	IN: 1	
492	✓	✗	✓	✗	✓	✓
	0	Cytoplasmic	1	0	IN: 0	
495	✗	✗	✓	✗	✓	✓
	2	Cytoplasmic	4	0	OUT: 0	
494	✓	✗	✗	✗	✓	✓

	0	Unknown	0	0	IN: 1	
489	✔	✘	✔	✔	✔	✔
	0	Cytoplasmic	6	6	OUT: 0	
488	✔	✘	✔	✘	✔	✔
	0	Cytoplasmic	1	0	OUT: 0	
491	✔	✘	✘	✘	✔	✔
	0	Cytoplasmic	0	0	IN: 0	
490	✔	✘	✔	✘	✔	✔
	0	Cytoplasmic	2	0	OUT: 0	
485	✔	✘	✘	✘	✔	✘
	0	Unknown	0	0	OUT: 0	
484	✔	✘	✘	✘	✔	✘
	0	Unknown	0	0	IN: 0	
487	✔	✘	✔	✘	✘	✔
	0	Cytoplasmic	12	0	OUT: 2	
486	✔	✘	✘	✔	✔	✔
	0	Cytoplasmic	0	4	OUT: 0	
481	✔	✘	✘	✔	✔	✔
	0	Unknown	0	1	OUT: 0	
480	✔	✘	✔	✘	✔	✘

	0	Unknown	1	0	OUT: 0	
483	✓	✗	✓	✗	✗	✓
	0	Cytoplasmic	19	0	OUT: 3	
482	✓	✗	✗	✗	✗	?
	0	CytoplasmicMembrane	0	0	OUT: 7	
550	✓	✗	✗	✗	✓	✓
	0	Cytoplasmic	0	0	OUT: 0	
551	✓	✗	✗	✗	✓	✗
	0	Unknown	0	0	OUT: 0	
548	✓	✗	✓	✗	✗	✓
	0	CytoplasmicMembrane	1	0	IN: 10	
549	✓	✗	✓	✗	✓	✓
	0	Cytoplasmic	25	0	IN: 0	
546	✓	✗	✗	✗	✓	✗
	0	Unknown	0	0	OUT: 0	
547	✓	✗	✓	✓	✗	✓
	0	CytoplasmicMembrane	4	21	IN: 6	
544	✓	✗	✗	✗	✗	?

	0	CytoplasmicMembrane	0	0	IN: 5	
545	✓	✗	✗	✗	✓	✗
	0	Unknown	0	0	OUT: 1	
558	✓	✗	✓	✗	✓	✓
	0	Cytoplasmic	4	0	OUT: 0	
559	✓	✗	✗	✗	✓	✗
	0	Cytoplasmic	0	0	OUT: 0	
556	✓	✗	✗	✗	✗	✓
	0	CytoplasmicMembrane	0	0	OUT: 2	
557	✓	✗	✗	✗	✗	✓
	0	Unknown	0	0	OUT: 3	
554	✗	✗	✓	✗	✗	✓
	250	CytoplasmicMembrane	5	0	OUT: 8	
555	✗	✗	✓	✗	✓	✓
	13	Cytoplasmic	2	0	OUT: 0	
552	✓	✗	✗	✗	✓	?
	0	Cytoplasmic	0	0	OUT: 0	
553	✓	✗	✓	✓	✓	✓

	0	Unknown	1	1	OUT: 0	
567	✓	✗	✗	✗	✓	?
	0	Unknown	0	0	OUT: 1	
566	✓	✗	✓	✗	✗	✓
	0	CytoplasmicMembrane	1	0	IN: 12	
565	✓	✗	✓	✗	✓	✓
	0	Cytoplasmic	2	0	OUT: 0	
564	✓	✗	✗	✗	✓	✗
	0	Cytoplasmic	0	0	OUT: 0	
563	✓	✗	✗	✗	✓	?
	0	Unknown	0	0	IN: 1	
562	✓	✗	✓	✓	✗	✓
	0	CytoplasmicMembrane	1	16	OUT: 11	
561	✓	✗	✓	✗	✓	?
	0	Cytoplasmic	1	0	OUT: 0	
560	✓	✗	✓	✗	✓	✗
	0	Unknown	1	0	OUT: 0	
575	✓	✗	✗	✗	✓	✓
	0	Cytoplasmic	0	0	OUT: 1	

574	✓	✗	✓	✗	✗	✓
	0	CytoplasmicMembrane	1	0	IN: 14	
573	✓	✓	✗	✓	✓	✗
	0	OuterMembrane	0	15	OUT: 0	
572	✓	✗	✓	✗	✗	✓
	0	CytoplasmicMembrane	12	0	IN: 12	
571	✓	✗	✓	✗	✓	✓
	0	Cytoplasmic	2	0	OUT: 0	
570	✓	✗	✗	✗	✓	✗
	0	Unknown	0	0	IN: 1	
569	✓	✗	✗	✗	✗	✗
	0	CytoplasmicMembrane	0	0	OUT: 4	
568	✓	✗	✗	✗	✓	?
	0	Unknown	0	0	OUT: 0	
516	✓	✗	✗	✗	✗	✓
	0	Periplasmic	0	0	IN: 2	
517	✓	✗	✗	✗	✓	✓
	0	Cytoplasmic	0	0	OUT: 0	
	✓	✗	✓	✗	✓	✓

518	0	Cytoplasmic	26	0	OUT: 0	
519	✓	✗	✗	✗	✓	?
	0	Cytoplasmic	0	0	OUT: 0	
512	✓	✗	✗	✗	✓	✗
	0	Cytoplasmic	0	0	OUT: 0	
513	✓	✗	✓	✗	✓	✓
	0	Cytoplasmic	1	0	IN: 0	
514	✗	✗	✓	✓	✓	✓
	9	Periplasmic	4	8	OUT: 0	
515	✓	✗	✗	✗	✓	✓
	0	Cytoplasmic	0	0	OUT: 0	
524	✓	✗	✗	✗	✓	✗
	0	Unknown	0	0	OUT: 0	
525	✓	✗	✓	✗	✓	✓
	0	Cytoplasmic	1	0	OUT: 0	
526	✓	✗	✗	✓	✓	✓
	0	Cytoplasmic	0	2	OUT: 0	
527	✓	✗	✓	✓	✓	✓
	0	CytoplasmicMembrane	17	55	OUT: 0	
520	✓	✗	✓	✗	✗	✓

	0	CytoplasmicMembrane	1	0	IN: 5	
521	✔	✘	✘	✘	✘	?
	0	CytoplasmicMembrane	0	0	IN: 6	
522	✔	✘	✘	✘	✔	✘
	0	Cytoplasmic	0	0	OUT: 0	
523	✘	✘	✔	✘	✘	✔
	6	CytoplasmicMembrane	2	0	OUT: 12	
533	✔	✘	✔	✔	✔	✔
	0	Cytoplasmic	1	14	OUT: 0	
532	✔	✘	✘	✘	✔	✘
	0	CytoplasmicMembrane	0	0	IN: 1	
535	✔	✔	✘	✘	✘	✘
	0	OuterMembrane	0	0	IN: 3	
534	✔	✔	✔	✔	✔	✔
	0	Extracellular	2	3	IN: 1	
529	✔	✘	✘	✘	✔	✘
	0	Cytoplasmic	0	0	OUT: 0	
528	✔	✘	✘	✘	✘	?

	0	Unknown	0	0	IN: 2	
531	✓	✗	✗	✗	✓	✓
	0	Cytoplasmic	0	0	OUT: 0	
530	✓	✗	✗	✗	✓	✓
	0	Unknown	0	0	OUT: 0	
541	✗	✗	✓	✗	✗	✓
	5	Cytoplasmic	8	0	OUT: 2	
540	✓	✗	✗	✗	✗	✓
	0	Cytoplasmic	0	0	OUT: 2	
543	✓	✗	✓	✗	✓	✓
	0	Cytoplasmic	12	0	OUT: 0	
542	✓	✗	✗	✓	✓	?
	0	Unknown	0	3	IN: 1	
537	✓	✗	✗	✗	✓	?
	0	Cytoplasmic	0	0	OUT: 0	
536	✓	✗	✗	✗	✗	?
	0	CytoplasmicMembrane	0	0	OUT: 3	
539	✗	✗	✓	✗	✗	✓
	250	CytoplasmicMembrane	1	0	OUT: 9	

538	✓	✗	✗	✗	✗	✗
	0	CytoplasmicMembrane	0	0	IN: 2	
610	✓	✗	✓	✗	✓	?
	0	Cytoplasmic	1	0	IN: 0	
611	✓	✗	✓	✓	✓	✓
	0	Cytoplasmic	14	2	IN: 0	
608	✓	✗	✓	✗	✓	✗
	0	Unknown	1	0	IN: 1	
609	✓	✗	✗	✓	✓	✓
	0	Cytoplasmic	0	1	OUT: 0	
614	✓	✗	✗	✗	✓	✓
	0	Unknown	0	0	IN: 1	
615	✓	✗	✓	✗	✓	✓
	0	CytoplasmicMembrane	1	0	OUT: 0	
612	✓	✗	✗	✗	✓	?
	0	Unknown	0	0	IN: 0	
613	✓	✗	✗	✗	✓	?
	0	Cytoplasmic	0	0	OUT: 0	
618	✓	✗	✓	✓	✓	✓

	0	Cytoplasmic	7	4	OUT: 0	
619	✓	✗	✗	✗	✓	?
	0	Unknown	0	0	IN: 1	
616	✓	✗	✗	✗	✗	?
	0	CytoplasmicMembrane	0	0	IN: 12	
617	✓	✗	✓	✗	✓	?
	0	Cytoplasmic	1	0	OUT: 0	
622	✓	✗	✗	✓	✓	?
	0	Cytoplasmic	0	2	OUT: 0	
623	✓	✗	✓	✗	✓	✓
	0	CytoplasmicMembrane	14	0	OUT: 0	
620	✓	✗	✗	✗	✓	✓
	0	Cytoplasmic	0	0	OUT: 0	
621	✓	✗	✓	✗	✗	✓
	0	CytoplasmicMembrane	1	0	IN: 5	
627	✓	✗	✓	✗	✗	✓
	0	CytoplasmicMembrane	1	0	IN: 5	
626	✓	✗	✓	✗	✓	✓

	0	CytoplasmicMembrane	2	0	OUT: 0	
625	✓	✗	✗	✗	✓	✗
	0	Unknown	0	0	IN: 1	
624	✗	✗	✓	✗	✓	✓
	7	Cytoplasmic	12	0	OUT: 0	
631	✓	✗	✓	✗	✓	✓
	0	Cytoplasmic	1	0	OUT: 0	
630	✓	✗	✗	✗	✓	✓
	0	Cytoplasmic	0	0	OUT: 0	
629	✓	✗	✗	✗	✗	✓
	0	CytoplasmicMembrane	0	0	IN: 2	
628	✓	✗	✗	✗	✓	✗
	0	Cytoplasmic	0	0	OUT: 0	
635	✓	✓	✓	✗	✓	✓
	0	OuterMembrane	1	0	OUT: 0	
634	✓	✗	✗	✗	✓	✗
	0	Unknown	0	0	IN: 0	
633	✓	✗	✗	✗	✗	✓
	0	CytoplasmicMem	0	0	OUT: 3	

		brane				
632	✓	✗	✗	✗	✓	?
	0	Cytoplasmic	0	0	OUT: 0	
639	✓	✗	✗	✗	✓	?
	0	Cytoplasmic	0	0	OUT: 0	
638	✓	✗	✗	✗	✓	✗
	0	Unknown	0	0	OUT: 0	
637	✓	✗	✓	✗	✓	✓
	0	Cytoplasmic	1	0	OUT: 0	
636	✓	✗	✗	✗	✓	?
	0	Unknown	0	0	OUT: 1	
576	✓	✗	✗	✗	✓	✓
	0	Cytoplasmic	0	0	OUT: 0	
577	✓	✗	✓	✗	✓	✓
	0	Cytoplasmic	1	0	OUT: 0	
578	✓	✗	✗	✓	✓	✗
	0	Unknown	0	3	IN: 1	
579	✓	✗	✗	✗	✓	✗
	0	Unknown	0	0	OUT: 0	
580	✓	✗	✗	✗	✗	✓

	0	CytoplasmicMembrane	0	0	IN: 10	
581	✔	✘	✘	✘	✔	✘
	0	Cytoplasmic	0	0	OUT: 0	
582	✔	✔	✘	✔	✔	✘
	0	Extracellular	0	14	IN: 1	
583	✔	✘	✔	✘	✔	✔
	0	Cytoplasmic	1	0	OUT: 0	
584	✔	✘	✘	✔	✔	?
	0	Periplasmic	0	3	OUT: 0	
585	✔	✘	✘	✘	✔	✘
	0	Cytoplasmic	0	0	OUT: 0	
586	✔	✘	✔	✘	✔	?
	0	Unknown	2	0	OUT: 1	
587	✔	✘	✘	✘	✔	?
	0	Cytoplasmic	0	0	IN: 0	
588	✔	✘	✘	✘	✔	✘
	0	Cytoplasmic	0	0	OUT: 0	
589	✘	✘	✔	✔	✔	✔
	10	Cytoplasmic	23	1	OUT: 0	
590	✔	✘	✘	✘	✘	✔

	0	CytoplasmicMembrane	0	0	OUT: 4	
591	✓	✗	✓	✗	✓	✓
	0	Unknown	1	0	OUT: 0	
593	✓	✗	✓	✗	✓	✗
	0	Cytoplasmic	1	0	OUT: 0	
592	✓	✗	✓	✗	✓	✓
	0	Cytoplasmic	24	0	OUT: 0	
595	✓	✗	✓	✗	✓	?
	0	Unknown	1	0	IN: 1	
594	✓	✗	✗	✗	✓	✓
	0	Unknown	0	0	IN: 0	
597	✓	✗	✓	✗	✓	✓
	0	Cytoplasmic	1	0	OUT: 0	
596	✓	✗	✗	✗	✓	✓
	0	Unknown	0	0	IN: 0	
599	✓	✗	✓	✓	✓	✓
	0	Unknown	96	8	OUT: 0	
598	✗	✗	✓	✓	✓	✓
	37	Cytoplasmic	1	3	OUT: 1	
601	✓	✗	✗	✗	✓	✓

	0	Unknown	0	0	OUT: 0	
600	✔	✘	✘	✘	✔	?
	0	Unknown	0	0	OUT: 0	
603	✔	✘	✔	✘	✔	✔
	0	Cytoplasmic	1	0	OUT: 0	
602	✔	✔	✘	✔	✔	✔
	0	Extracellular	0	2	IN: 1	
605	✔	✘	✔	✘	✔	✔
	0	Cytoplasmic	1	0	IN: 0	
604	✔	✘	✘	✘	✔	✘
	0	Unknown	0	0	IN: 1	
607	✔	✘	✔	✘	✔	✔
	0	Cytoplasmic	1	0	IN: 0	
606	✔	✘	✔	✘	✔	✔
	0	Unknown	1	0	IN: 0	
687	✔	✘	✘	✘	✔	?
	0	Cytoplasmic	0	0	IN: 1	
686	✔	✘	✔	✘	✘	?
	0	CytoplasmicMembrane	1	0	IN: 2	
685	✘	✘	✔	✘	✔	✔

	7	Cytoplasmic	26	0	OUT: 0	
684	✓	✗	✗	✗	✓	✓
	0	Unknown	0	0	OUT: 0	
683	✓	✗	✗	✗	✓	✓
	0	Cytoplasmic	0	0	OUT: 0	
682	✓	✗	✗	✗	✓	?
	0	Unknown	0	0	IN: 1	
681	✓	✗	✗	✗	✓	?
	0	Cytoplasmic	0	0	OUT: 0	
680	✓	✗	✗	✗	✓	✓
	0	Cytoplasmic	0	0	OUT: 0	
679	✓	✗	✗	✗	✓	✗
	0	Cytoplasmic	0	0	OUT: 0	
678	✓	✗	✓	✗	✓	✓
	0	Cytoplasmic	12	0	IN: 0	
677	✓	✗	✓	✗	✗	✓
	0	Cytoplasmic	4	0	OUT: 2	
676	✓	✗	✗	✗	✓	?
	0	Cytoplasmic	0	0	IN: 1	
675	✓	✗	✗	✗	✓	✓

	0	Cytoplasmic	0	0	OUT: 1	
674	✔	✘	✔	✘	✔	✔
	0	Cytoplasmic	4	0	OUT: 0	
673	✔	✘	✘	✔	✔	?
	0	Cytoplasmic	0	3	OUT: 0	
672	✔	✘	✘	✘	✘	?
	0	CytoplasmicMembrane	0	0	OUT: 6	
702	✔	✘	✘	✔	✔	✘
	0	Unknown	0	3	OUT: 1	
703	✔	✘	✘	✘	✘	✔
	0	CytoplasmicMembrane	0	0	IN: 11	
700	✔	✘	✘	✔	✘	✘
	0	CytoplasmicMembrane	0	3	IN: 2	
701	✔	✘	✔	✘	✔	✘
	0	Cytoplasmic	1	0	OUT: 0	
698	✔	✘	✘	✘	✔	✘
	0	Unknown	0	0	OUT: 0	
699	✔	✘	✘	✘	✔	✔

	0	Cytoplasmic	0	0	OUT: 0	
696	✗	✗	✓	✗	✓	✓
	2	Cytoplasmic	5	0	IN: 1	
697	✓	✗	✗	✓	✗	✓
	0	CytoplasmicMembrane	0	18	IN: 7	
694	✓	✓	✗	✓	✓	✓
	0	Extracellular	0	14	OUT: 0	
695	✓	✗	✓	✗	✓	✓
	0	Cytoplasmic	2	0	OUT: 0	
692	✓	✗	✓	✓	✓	✓
	0	Cytoplasmic	3	11	IN: 0	
693	✓	✗	✗	✗	✓	✗
	0	Unknown	0	0	OUT: 0	
690	✓	✓	✗	✗	✓	✓
	0	OuterMembrane	0	0	OUT: 0	
691	✓	✗	✗	✗	✓	?
	0	Cytoplasmic	0	0	OUT: 0	
688	✓	✗	✓	✓	✓	✓
	0	CytoplasmicMembrane	44	48	OUT: 0	

689	✓	✗	✗	✗	✓	✗
	0	Unknown	0	0	OUT: 0	
653	✓	✗	✓	✗	✓	✓
	0	Cytoplasmic	1	0	OUT: 0	
652	✗	✗	✓	✗	✓	✓
	8	Cytoplasmic	18	0	OUT: 0	
655	✓	✗	✗	✗	✓	✗
	0	Unknown	0	0	OUT: 0	
654	✓	✗	✓	✗	✓	✓
	0	Cytoplasmic	2	0	IN: 0	
649	✓	✗	✗	✓	✓	✓
	0	CytoplasmicMembrane	0	1	IN: 0	
648	✓	✗	✗	✗	✓	✗
	0	Cytoplasmic	0	0	IN: 1	
651	✗	✗	✓	✓	✓	✓
	18	Cytoplasmic	23	3	IN: 0	
650	✓	✓	✗	✓	✓	✓
	0	OuterMembrane	0	1	OUT: 0	
645	✓	✗	✓	✗	✓	?
	0	Cytoplasmic	1	0	OUT: 1	

644	✓	✗	✗	✗	✓	✗
	0	Cytoplasmic	0	0	IN: 0	
647	✓	✗	✗	✗	✓	✓
	0	Unknown	0	0	IN: 1	
646	✓	✗	✗	✗	✓	✗
	0	Cytoplasmic	0	0	OUT: 0	
641	✓	✗	✗	✗	✓	✓
	0	CytoplasmicMembrane	0	0	OUT: 0	
640	✓	✗	✓	✗	✓	✓
	0	Cytoplasmic	18	0	OUT: 0	
643	✓	✗	✓	✗	✓	✓
	0	Cytoplasmic	2	0	OUT: 0	
642	✓	✗	✗	✗	✗	✗
	0	CytoplasmicMembrane	0	0	IN: 4	
668	✓	✗	✓	✗	✓	✓
	0	Cytoplasmic	3	0	OUT: 0	
669	✗	✗	✓	✗	✓	✓
	18	Cytoplasmic	24	0	OUT: 0	
670	✓	✗	✗	✗	✓	✓

	0	Cytoplasmic	0	0	OUT: 0	
671						
	0	Cytoplasmic	0	0	IN: 1	
664						
	0	Cytoplasmic	0	0	OUT: 0	
665						
	0	Unknown	0	0	OUT: 1	
666						
	0	Cytoplasmic	1	0	OUT: 0	
667						
	0	Unknown	0	0	IN: 1	
660						
	0	Unknown	1	0	IN: 1	
661						
	0	Cytoplasmic	0	0	OUT: 0	
662						
	0	Unknown	0	0	IN: 3	
663						
	0	Cytoplasmic	0	0	OUT: 0	
656						

	0	CytoplasmicMembrane	0	0	IN: 11	
657						
	15	Cytoplasmic	17	0	OUT: 0	
658						
	0	Unknown	0	0	OUT: 0	
659						
	0	Cytoplasmic	0	0	OUT: 0	
747						
	0	Cytoplasmic	6	0	OUT: 0	
746						
	0	Cytoplasmic	0	3	OUT: 0	
745						
	0	Cytoplasmic	1	0	OUT: 0	
744						
	0	Cytoplasmic	0	0	OUT: 0	
751						
	0	OuterMembrane	0	15	IN: 1	
750						
	0	Unknown	0	0	IN: 1	
749						

	0	Cytoplasmic	16	0	OUT: 0	
748	✓	✗	✓	✗	✓	✓
	0	Unknown	1	0	IN: 0	
739	✓	✗	✓	✗	✓	✓
	0	Cytoplasmic	7	0	OUT: 0	
738	✓	✗	✓	✗	✓	✗
	0	Unknown	1	0	IN: 0	
737	✓	✗	✓	✗	✓	?
	0	Unknown	1	0	OUT: 0	
736	✓	✗	✗	✓	✓	?
	0	Unknown	0	3	IN: 1	
743	✓	✗	✓	✗	✓	✓
	0	Cytoplasmic	18	0	OUT: 0	
742	✓	✗	✗	✗	✓	✗
	0	Unknown	0	0	IN: 0	
741	✓	✗	✗	✓	✓	✗
	0	Unknown	0	10	OUT: 0	
740	✓	✗	✗	✓	✓	✗
	0	Cytoplasmic	0	3	OUT: 0	
762	✓	✗	✗	✗	✓	✓

	0	Cytoplasmic	0	0	OUT: 0	
763	✓	✗	✓	✗	✓	✓
	0	Cytoplasmic	1	0	OUT: 0	
760	✓	✗	✗	✗	✓	?
	0	Unknown	0	0	OUT: 0	
766	✓	✗	✓	✗	✓	✗
	0	Unknown	1	0	OUT: 0	
767	✓	✗	✗	✗	✗	✓
	0	Cytoplasmic	0	0	OUT: 2	
764	✗	✗	✓	✗	✓	✓
	54	Cytoplasmic	13	0	OUT: 0	
765	✓	✗	✗	✓	✗	✓
	0	CytoplasmicMembrane	0	1	OUT: 9	
754	✓	✗	✗	✓	✓	✓
	0	CytoplasmicMembrane	0	6	OUT: 0	
755	✓	✗	✗	✓	✗	✓
	0	CytoplasmicMembrane	0	18	IN: 4	
752	✓	✗	✓	✗	✓	✓

	0	Cytoplasmic	12	0	IN: 0	
753	✓	✗	✗	✗	✓	?
	0	Cytoplasmic	0	0	OUT: 0	
758	✓	✗	✗	✗	✓	✓
	0	Cytoplasmic	0	0	OUT: 0	
759	✓	✗	✗	✗	✓	?
	0	Unknown	0	0	OUT: 0	
756	✗	✗	✓	✗	✓	✓
	32	Cytoplasmic	19	0	IN: 1	
757	✓	✗	✓	✗	✗	✓
	0	CytoplasmicMembrane	2	0	OUT: 7	
713	✓	✗	✗	✗	✓	?
	0	Unknown	0	0	IN: 1	
712	✓	✗	✗	✓	✓	✓
	0	Cytoplasmic	0	2	OUT: 0	
715	✓	✗	✗	✗	✓	✓
	0	Cytoplasmic	0	0	OUT: 0	
714	✗	✗	✗	✗	✗	✓
	11	CytoplasmicMembrane	0	0	IN: 15	

717	✓	✗	✗	✗	✓	✓
	0	Unknown	0	0	OUT: 0	
716	✓	✗	✓	✗	✓	✓
	0	Cytoplasmic	1	0	OUT: 0	
719	✓	✗	✗	✗	✓	?
	0	Unknown	0	0	OUT: 0	
718	✓	✗	✓	✓	✗	✓
	0	CytoplasmicMembrane	13	4	IN: 3	
705	✓	✗	✗	✗	✓	✓
	0	Cytoplasmic	0	0	OUT: 0	
704	✗	✗	✓	✗	✓	✓
	2	Cytoplasmic	25	0	OUT: 0	
707	✓	✗	✗	✗	✓	?
	0	Cytoplasmic	0	0	OUT: 0	
706	✓	✗	✓	✗	✗	?
	0	CytoplasmicMembrane	2	0	OUT: 3	
709	✓	✗	✓	✗	✓	?
	0	Unknown	1	0	OUT: 0	
708	✓	✗	✓	✗	✓	✓

	0	Cytoplasmic	1	0	OUT: 0	
711	✔	✘	✘	✘	✔	✘
	0	Unknown	0	0	OUT: 0	
710	✔	✘	✔	✘	✔	✘
	0	Unknown	1	0	OUT: 0	
728	✔	✘	✔	✘	✔	✔
	0	Cytoplasmic	17	0	OUT: 0	
729	✔	✘	✔	✘	✔	?
	0	Cytoplasmic	1	0	OUT: 0	
730	✔	✘	✔	✘	✔	✔
	0	Cytoplasmic	17	0	OUT: 0	
731	✔	✘	✘	✘	✔	✔
	0	Cytoplasmic	0	0	OUT: 0	
732	✔	✘	✘	✘	✔	✔
	0	Unknown	0	0	IN: 0	
733	✔	✘	✘	✘	✔	✘
	0	Unknown	0	0	OUT: 0	
734	✔	✘	✔	✘	✔	✘
	0	Unknown	1	0	OUT: 0	
735	✔	✘	✘	✘	✔	✘

	0	Unknown	0	0	OUT: 0	
720	✔	✘	✘	✘	✔	?
	0	Cytoplasmic	0	0	OUT: 0	
721	✔	✘	✘	✘	✘	?
	0	CytoplasmicMembrane	0	0	IN: 6	
722	✔	✘	✘	✘	✔	✘
	0	Cytoplasmic	0	0	OUT: 0	
723	✔	✔	✘	✔	✔	✔
	0	Extracellular	0	2	IN: 1	
724	✘	✘	✔	✘	✔	✔
	24	Cytoplasmic	34	0	IN: 0	
725	✔	✔	✘	✔	✔	✘
	0	OuterMembrane	0	15	OUT: 0	
726	✔	✘	✘	✔	✔	?
	0	Cytoplasmic	0	1	OUT: 0	
727	✔	✘	✘	✔	✘	✔
	0	CytoplasmicMembrane	0	1	OUT: 2	
821	✘	✘	✔	✘	✔	✔
	1	Cytoplasmic	7	0	OUT: 0	

820	✔	✖	✔	✖	✔	✔
	0	CytoplasmicMembrane	2	0	OUT: 1	
823	✔	✖	✖	✖	✔	✖
	0	Cytoplasmic	0	0	IN: 1	
822	✔	✖	✖	✖	✖	✔
	0	CytoplasmicMembrane	0	0	OUT: 6	
817	✔	✖	✖	✖	✔	✖
	0	Cytoplasmic	0	0	OUT: 0	
816	✔	✖	✖	✖	✔	?
	0	Cytoplasmic	0	0	OUT: 0	
819	✔	✖	✔	✖	✔	✔
	0	Cytoplasmic	2	0	OUT: 1	
818	✔	✖	✖	✖	✔	✔
	0	Cytoplasmic	0	0	OUT: 0	
829	✔	✖	✖	✖	✔	?
	0	Cytoplasmic	0	0	OUT: 0	
828	✔	✖	✔	✖	✔	✖
	0	Cytoplasmic	1	0	OUT: 0	
831	✔	✖	✖	✖	✔	✔

	0	Cytoplasmic	0	0	OUT: 0	
830	✓	✗	✗	✗	✗	✗
	0	Unknown	0	0	OUT: 2	
825	✓	✓	✓	✓	✓	?
	0	OuterMembrane	1	3	IN: 1	
824	✓	✗	✗	✗	✗	✓
	0	CytoplasmicMem<br>brane	0	0	IN: 7	
827	✓	✗	✓	✗	✓	?
	0	Unknown	1	0	IN: 1	
826	✗	✗	✓	✗	✓	?
	5	Cytoplasmic	1	0	OUT: 0	
805	✓	✓	✗	✗	✓	✗
	0	OuterMembrane	0	0	IN: 1	
806	✗	✗	✓	✗	✓	✓
	1	Cytoplasmic	5	0	OUT: 0	
807	✗	✗	✓	✗	✓	✓
	3	Cytoplasmic	7	0	OUT: 0	
800	✓	✗	✗	✗	✓	?
	0	Cytoplasmic	0	0	OUT: 0	
801	✓	✗	✗	✗	✓	✓

	0	Unknown	0	0	OUT: 0	
802	✔	✘	✘	✘	✔	?
	0	Cytoplasmic	0	0	OUT: 0	
803	✔	✘	✔	✘	✔	✔
	0	Cytoplasmic	12	0	OUT: 0	
812	✔	✘	✘	✘	✘	?
	0	Unknown	0	0	IN: 3	
813	✔	✘	✘	✘	✘	✘
	0	CytoplasmicMembrane	0	0	IN: 5	
814	✔	✘	✔	✘	✘	✘
	0	CytoplasmicMembrane	1	0	IN: 3	
815	✔	✘	✔	✘	✔	✔
	0	Unknown	1	0	OUT: 0	
808	✘	✘	✔	✘	✔	✔
	2	Cytoplasmic	1	0	OUT: 0	
809	✔	✘	✘	✘	✔	✔
	0	Cytoplasmic	0	0	OUT: 0	
810	✔	✘	✔	✘	✔	✔
	0	Cytoplasmic	1	0	OUT: 1	

811	✓	✗	✗	✓	✗	✗
	0	CytoplasmicMembrane	0	1	OUT: 7	
791	✓	✗	✓	✓	✓	✓
	0	Cytoplasmic	2	29	IN: 0	
790	✓	✗	✗	✗	✓	?
	0	Cytoplasmic	0	0	OUT: 0	
789	✓	✗	✗	✗	✗	?
	0	Cytoplasmic	0	0	OUT: 2	
788	✓	✗	✓	✗	✓	✓
	0	Cytoplasmic	1	0	IN: 1	
787	✓	✗	✗	✗	✓	?
	0	Unknown	0	0	OUT: 0	
786	✓	✗	✗	✗	✓	✓
	0	Cytoplasmic	0	0	OUT: 0	
785	✓	✗	✓	✗	✓	✓
	0	Cytoplasmic	1	0	OUT: 0	
784	✓	✗	✗	✗	✓	✓
	0	Cytoplasmic	0	0	OUT: 0	
799	✓	✗	✗	✗	✓	?
	0	Unknown	0	0	OUT: 0	

798	✓	✗	✗	✓	✓	?
	0	Unknown	0	1	IN: 1	
797	✓	✗	✓	✗	✗	✓
	0	CytoplasmicMembrane	3	0	OUT: 4	
796	✓	✗	✗	✗	✓	✗
	0	Unknown	0	0	OUT: 0	
795	✓	✗	✗	✗	✓	✗
	0	Cytoplasmic	0	0	OUT: 0	
794	✓	✗	✓	✗	✓	✓
	0	Cytoplasmic	1	0	OUT: 0	
793	✗	✗	✓	✗	✓	✓
	1	Cytoplasmic	4	0	OUT: 0	
792	✓	✗	✓	✓	✓	✓
	0	Cytoplasmic	27	2	OUT: 0	
774	✗	✗	✓	✓	✓	✓
	4	Cytoplasmic	11	3	OUT: 1	
775	✓	✗	✓	✓	✓	✓
	0	Cytoplasmic	2	1	OUT: 0	
772	✓	✗	✓	✗	✗	✗
	0	CytoplasmicMem	1	0	IN: 4	

		brane				
773	✘	✘	✔	✘	✔	✔
	11	Cytoplasmic	27	0	OUT: 1	
770	✔	✘	✘	✘	✔	✘
	0	Unknown	0	0	IN: 1	
771	✔	✘	✘	✘	✘	✘
	0	Unknown	0	0	OUT: 2	
768	✔	✘	✘	✘	✘	?
	0	CytoplasmicMembrane	0	0	OUT: 4	
769	✔	✘	✘	✘	✔	✔
	0	Unknown	0	0	OUT: 1	
782	✔	✘	✘	✘	✔	?
	0	Cytoplasmic	0	0	OUT: 0	
783	✔	✘	✔	✘	✔	✔
	0	Cytoplasmic	1	0	OUT: 0	
780	✔	✘	✔	✘	✔	✔
	0	Unknown	2	0	OUT: 0	
781	✔	✘	✔	✘	✔	✔
	0	Unknown	1	0	IN: 1	
778	✔	✘	✔	✘	✔	✔

	0	Cytoplasmic	7	0	OUT: 0	
779	✓	✗	✓	✓	✓	✓
	0	CytoplasmicMembrane	22	24	OUT: 0	
776	✓	✗	✗	✗	✓	?
	0	Cytoplasmic	0	0	OUT: 0	
777	✓	✗	✓	✗	✗	✗
	0	CytoplasmicMembrane	1	0	IN: 4	
881	✓	✗	✓	✗	✓	✓
	0	Unknown	1	0	IN: 0	
880	✓	✗	✓	✗	✓	✓
	0	Cytoplasmic	3	0	OUT: 0	
883	✓	✗	✓	✗	✗	✓
	0	CytoplasmicMembrane	1	0	IN: 5	
882	✓	✗	✓	✗	✓	✗
	0	CytoplasmicMembrane	1	0	IN: 1	
885	✗	✗	✗	✗	✓	✓
	1	Cytoplasmic	0	0	OUT: 0	
884	✓	✗	✗	✗	✗	✗

	0	Unknown	0	0	OUT: 4	
887	✔	✖	✖	✔	✔	✔
	0	Cytoplasmic	0	9	OUT: 0	
886	✔	✖	✖	✖	✔	?
	0	Cytoplasmic	0	0	OUT: 0	
889	✔	✖	✔	✔	✔	✔
	0	CytoplasmicMembrane	15	5	OUT: 0	
888	✔	✖	✖	✖	✖	?
	0	CytoplasmicMembrane	0	0	IN: 5	
891	✔	✖	✔	✖	✔	✔
	0	Cytoplasmic	15	0	OUT: 0	
890	✔	✖	✔	✖	✔	✔
	0	Unknown	1	0	OUT: 0	
893	✔	✖	✔	✔	✔	✔
	0	Cytoplasmic	4	1	OUT: 0	
892	✔	✖	✔	✔	✔	✔
	0	Cytoplasmic	16	39	OUT: 1	
895	✔	✖	✔	✔	✔	✔
	0	Unknown	1	3	OUT: 0	

894	✓	✗	✗	✗	✓	✗
	0	Cytoplasmic	0	0	IN: 1	
864	✗	✗	✓	✗	✓	✓
	1	Cytoplasmic	3	0	OUT: 0	
865	✓	✗	✓	✗	✓	✓
	0	Cytoplasmic	7	0	OUT: 0	
866	✓	✗	✗	✗	✓	✓
	0	Cytoplasmic	0	0	OUT: 0	
867	✓	✗	✗	✗	✗	✗
	0	CytoplasmicMembrane	0	0	IN: 3	
868	✓	✗	✓	✓	✗	✓
	0	CytoplasmicMembrane	2	2	IN: 6	
869	✓	✗	✗	✓	✗	✗
	0	Unknown	0	3	OUT: 2	
870	✓	✗	✗	✗	✓	?
	0	Cytoplasmic	0	0	IN: 0	
871	✓	✗	✓	✗	✓	✓
	0	Cytoplasmic	2	0	OUT: 0	
872	✓	✗	✓	✗	✓	✓

	0	Unknown	1	0	IN: 0	
873	✓	✗	✓	✗	✓	✓
	0	Cytoplasmic	1	0	OUT: 0	
874	✓	✓	✗	✗	✓	✗
	0	OuterMembrane	0	0	OUT: 0	
875	✓	✗	✗	✓	✓	✓
	0	Cytoplasmic	0	4	OUT: 0	
876	✓	✗	✗	✓	✗	✗
	0	CytoplasmicMembrane	0	3	IN: 12	
877	✓	✗	✗	✓	✗	?
	0	Cytoplasmic	0	4	IN: 5	
878	✓	✗	✗	✗	✓	?
	0	Cytoplasmic	0	0	OUT: 0	
879	✓	✗	✗	✗	✓	✓
	0	Unknown	0	0	IN: 1	
851	✓	✗	✗	✓	✓	?
	0	Cytoplasmic	0	3	OUT: 0	
850	✓	✗	✓	✗	✓	✓
	0	Cytoplasmic	5	0	OUT: 0	
849	✓	✗	✓	✗	✓	✓

	0	Cytoplasmic	4	0	IN: 0	
848	✓	✗	✗	✓	✓	✓
	0	Cytoplasmic	0	12	OUT: 0	
855	✓	✗	✓	✗	✓	?
	0	Cytoplasmic	1	0	OUT: 0	
854	✓	✗	✗	✗	✗	✓
	0	CytoplasmicMembrane	0	0	IN: 12	
853	✓	✗	✗	✗	✓	✗
	0	CytoplasmicMembrane	0	0	IN: 1	
852	✓	✗	✗	✗	✓	?
	0	Cytoplasmic	0	0	OUT: 0	
859	✓	✗	✓	✓	✓	✓
	0	Cytoplasmic	1	1	IN: 1	
858	✗	✗	✓	✗	✓	✓
	11	Cytoplasmic	1	0	IN: 1	
857	✓	✗	✗	✗	✓	✗
	0	Unknown	0	0	IN: 0	
856	✓	✗	✗	✗	✓	?
	0	Cytoplasmic	0	0	OUT: 1	

863	✓	✗	✓	✗	✓	✓
	0	Cytoplasmic	1	0	OUT: 0	
862	✓	✗	✗	✗	✓	✗
	0	Cytoplasmic	0	0	IN: 0	
861	✓	✗	✓	✗	✓	✓
	0	Cytoplasmic	11	0	OUT: 0	
860	✓	✗	✓	✗	✓	✓
	0	Cytoplasmic	1	0	OUT: 0	
834	✓	✗	✓	✗	✓	✓
	0	Cytoplasmic	10	0	OUT: 0	
835	✓	✗	✗	✓	✓	✓
	0	Cytoplasmic	0	2	OUT: 0	
832	✓	✗	✗	✗	✓	✗
	0	Unknown	0	0	IN: 1	
833	✓	✗	✗	✗	✓	✓
	0	Unknown	0	0	OUT: 0	
838	✗	✗	✓	✗	✗	✓
	4	Cytoplasmic	13	0	IN: 3	
839	✓	✗	✓	✓	✓	✓
	0	Cytoplasmic	22	2	OUT: 0	

836	✔	✘	✘	✘	✔	✔
	0	Cytoplasmic	0	0	OUT: 0	
837	✔	✔	✔	✔	✔	✔
	0	Extracellular	1	71	OUT: 0	
842	✔	✘	✔	✘	✔	✔
	0	Cytoplasmic	2	0	OUT: 0	
843	✔	✘	✘	✘	✔	✔
	0	CytoplasmicMembrane	0	0	OUT: 1	
840	✔	✘	✘	✘	✘	?
	0	Unknown	0	0	OUT: 2	
841	✔	✘	✔	✘	✔	✔
	0	Cytoplasmic	8	0	OUT: 0	
846	✔	✘	✔	✘	✔	✔
	0	Cytoplasmic	1	0	OUT: 0	
847	✔	✘	✔	✘	✔	✔
	0	Cytoplasmic	1	0	OUT: 1	
844	✔	✘	✘	✔	✘	?
	0	CytoplasmicMembrane	0	2	IN: 12	
845	✔	✔	✘	✔	✘	✘

	0	OuterMembrane	0	4	OUT: 2	
956	✗	✗	✓	✓	✓	✓
	3	Periplasmic	10	1	OUT: 0	
957	✓	✗	✗	✗	✓	✗
	0	Cytoplasmic	0	0	OUT: 0	
958	✓	✗	✗	✗	✓	?
	0	Cytoplasmic	0	0	OUT: 0	
959	✓	✗	✗	✗	✓	✓
	0	Unknown	0	0	OUT: 0	
952	✓	✗	✗	✗	✓	✗
	0	Unknown	0	0	IN: 0	
953	✓	✗	✓	✗	✓	✓
	0	Cytoplasmic	1	0	OUT: 0	
954	✓	✗	✗	✗	✓	✓
	0	Unknown	0	0	OUT: 1	
955	✓	✗	✗	✗	✓	✓
	0	Cytoplasmic	0	0	OUT: 0	
948	✓	✗	✗	✗	✓	?
	0	Cytoplasmic	0	0	IN: 0	
949	✓	✗	✓	✗	✓	✓

	0	Unknown	1	0	OUT: 1	
950	✔	✘	✘	✘	✔	✘
	0	Unknown	0	0	OUT: 0	
951	✔	✘	✘	✘	✔	✔
	0	Cytoplasmic	0	0	OUT: 0	
944	✔	✘	✔	✘	✘	✘
	0	Unknown	1	0	IN: 3	
945	✔	✘	✘	✘	✔	?
	0	Unknown	0	0	OUT: 0	
946	✔	✘	✔	✘	✔	?
	0	Cytoplasmic	1	0	OUT: 0	
947	✘	✘	✔	✘	✔	✔
	4	CytoplasmicMembrane	4	0	OUT: 1	
941	✔	✘	✘	✘	✘	?
	0	CytoplasmicMembrane	0	0	IN: 12	
940	✔	✘	✘	✘	✘	✔
	0	CytoplasmicMembrane	0	0	IN: 11	
943	✔	✘	✔	✘	✔	?

	0	CytoplasmicMem brane	1	0	OUT: 0	
942	✔	✘	✔	✘	✔	✔
	0	Cytoplasmic	1	0	OUT: 0	
937	✔	✘	✔	✘	✔	✔
	0	Cytoplasmic	1	0	OUT: 0	
936	✔	✘	✘	✘	✔	?
	0	Cytoplasmic	0	0	OUT: 0	
939	✔	✘	✔	✘	✔	✔
	0	Cytoplasmic	21	0	OUT: 0	
938	✔	✔	✘	✘	✔	?
	0	Extracellular	0	0	IN: 1	
933	✔	✘	✘	✘	✔	✔
	0	Cytoplasmic	0	0	IN: 0	
932	✔	✘	✘	✘	✘	✘
	0	CytoplasmicMem brane	0	0	OUT: 6	
935	✔	✘	✔	✔	✔	✔
	0	Cytoplasmic	27	9	IN: 0	
934	✔	✘	✘	✔	✘	✔
	0	CytoplasmicMem	0	1	OUT: 5	

		brane				
929	✓	✗	✓	✗	✓	✓
	0	Cytoplasmic	28	0	OUT: 0	
928	✓	✗	✗	✗	✓	?
	0	Unknown	0	0	OUT: 0	
931	✓	✗	✓	✗	✓	✗
	0	Cytoplasmic	1	0	OUT: 1	
930	✓	✗	✗	✗	✓	✓
	0	Unknown	0	0	OUT: 0	
926	✓	✗	✓	✓	✓	✓
	0	Cytoplasmic	2	2	OUT: 0	
927	✓	✗	✗	✗	✗	?
	0	Unknown	0	0	OUT: 2	
924	✓	✓	✗	✗	✓	✗
	0	OuterMembrane	0	0	IN: 1	
925	✓	✗	✓	✗	✗	✓
	0	CytoplasmicMem brane	1	0	OUT: 7	
922	✓	✗	✓	✗	✓	✓
	0	Cytoplasmic	7	0	IN: 1	
923	✓	✗	✓	✓	✓	✓

	0	Cytoplasmic	4	1	OUT: 0	
920	✓	✗	✓	✓	✓	✓
	0	Cytoplasmic	5	2	OUT: 0	
921	✗	✗	✓	✓	✓	✓
	1	Cytoplasmic	22	2	OUT: 0	
918	✓	✗	✗	✗	✗	✓
	0	CytoplasmicMembrane	0	0	IN: 12	
919	✓	✗	✓	✓	✓	✓
	0	CytoplasmicMembrane	1	1	IN: 1	
916	✓	✗	✓	✗	✓	✓
	0	Cytoplasmic	5	0	OUT: 0	
917	✓	✗	✓	✗	✓	✓
	0	Cytoplasmic	1	0	OUT: 0	
914	✓	✗	✗	✓	✓	✓
	0	Cytoplasmic	0	9	OUT: 0	
915	✓	✗	✓	✗	✗	✓
	0	Cytoplasmic	1	0	IN: 2	
912	✓	✗	✗	✗	✗	✓
	0	CytoplasmicMem	0	0	OUT: 11	

		brane				
913	✔	✖	✖	✖	✔	✔
	0	Unknown	0	0	OUT: 1	
911	✔	✔	✖	✖	✔	✖
	0	OuterMembrane	0	0	OUT: 0	
910	✔	✖	✖	✖	✖	?
	0	Periplasmic	0	0	IN: 3	
909	✔	✖	✔	✖	✔	✔
	0	Cytoplasmic	11	0	IN: 0	
908	✔	✖	✖	✖	✔	?
	0	Cytoplasmic	0	0	OUT: 1	
907	✔	✔	✔	✔	✔	?
	0	OuterMembrane	1	1	IN: 1	
906	✔	✖	✖	✖	✖	?
	0	CytoplasmicMembrane	0	0	OUT: 4	
905	✔	✖	✖	✖	✔	✖
	0	Unknown	0	0	IN: 1	
904	✔	✖	✔	✖	✔	?
	0	CytoplasmicMembrane	1	0	OUT: 0	

903	✓	✗	✓	✗	✓	✓
	0	Cytoplasmic	1	0	OUT: 0	
902	✓	✗	✗	✗	✓	✗
	0	Unknown	0	0	IN: 1	
901	✓	✗	✓	✗	✓	✓
	0	CytoplasmicMembrane	42	0	IN: 0	
900	✓	✗	✓	✗	✓	✓
	0	Cytoplasmic	5	0	OUT: 0	
899	✓	✗	✗	✗	✗	?
	0	CytoplasmicMembrane	0	0	IN: 12	
898	✓	✗	✗	✓	✗	?
	0	CytoplasmicMembrane	0	5	OUT: 4	
897	✓	✗	✓	✗	✗	✓
	0	CytoplasmicMembrane	2	0	IN: 18	
896	✓	✗	✓	✗	✓	✓
	0	Cytoplasmic	17	0	IN: 0	
1016	✓	✗	✗	✗	✗	?
	0	CytoplasmicMem	0	0	IN: 8	

		brane				
1017	✗	✗	✓	✗	✗	✓
	5	Cytoplasmic	13	0	OUT: 3	
1018	✓	✗	✓	✓	✓	✓
	0	Cytoplasmic	11	2	OUT: 0	
1019	✓	✗	✗	✗	✓	✗
	0	Cytoplasmic	0	0	OUT: 0	
1020	✓	✗	✗	✗	✓	✗
	0	Unknown	0	0	OUT: 0	
1021	✓	✗	✗	✗	✓	?
	0	Cytoplasmic	0	0	OUT: 0	
1022	✓	✗	✓	✓	✓	?
	0	Cytoplasmic	1	5	IN: 1	
1023	✓	✗	✗	✗	✓	✗
	0	Cytoplasmic	0	0	IN: 1	
1008	✓	✓	✗	✗	✓	?
	0	OuterMembrane	0	0	OUT: 0	
1009	✗	✗	✓	✗	✓	✓
	15	CytoplasmicMem brane	6	0	OUT: 0	
1010	✗	✗	✓	✓	✓	✓

	6	Cytoplasmic	1	10	OUT: 0	
1011	✓	✗	✗	✗	✓	?
	0	Cytoplasmic	0	0	OUT: 0	
1012	✓	✗	✓	✗	✓	?
	0	Cytoplasmic	4	0	OUT: 0	
1013	✓	✗	✗	✗	✗	?
	0	CytoplasmicMembrane	0	0	IN: 8	
1014	✓	✗	✗	✗	✓	?
	0	Unknown	0	0	OUT: 0	
1015	✓	✗	✗	✗	✗	?
	0	CytoplasmicMembrane	0	0	IN: 5	
1001	✓	✗	✗	✗	✗	?
	0	Cytoplasmic	0	0	IN: 2	
1000	✓	✓	✗	✓	✓	✗
	0	OuterMembrane	0	15	OUT: 0	
1003	✓	✗	✗	✗	✓	✓
	0	Cytoplasmic	0	0	OUT: 0	
1002	✗	✗	✓	✗	✓	✓
	14	Cytoplasmic	23	0	OUT: 0	

1005	✓	✗	✗	✗	✓	?
	0	Unknown	0	0	OUT: 1	
1004	✓	✗	✓	✓	✗	✓
	0	Cytoplasmic	11	1	IN: 3	
1007	✓	✗	✗	✗	✓	✓
	0	Cytoplasmic	0	0	OUT: 0	
1006	✓	✗	✓	✗	✓	✓
	0	Cytoplasmic	4	0	OUT: 0	
993	✓	✗	✗	✗	✓	✓
	0	Unknown	0	0	OUT: 0	
992	✓	✗	✓	✗	✓	✗
	0	Cytoplasmic	1	0	OUT: 0	
995	✓	✓	✓	✓	✓	?
	0	OuterMembrane	1	1	IN: 1	
994	✓	✗	✗	✗	✓	✓
	0	Unknown	0	0	OUT: 0	
997	✓	✗	✗	✗	✗	✗
	0	CytoplasmicMembrane	0	0	IN: 4	
996	✓	✗	✗	✗	✓	?
	0	Cytoplasmic	0	0	OUT: 0	

999	✓	✗	✓	✗	✗	✓
	0	CytoplasmicMembrane	1	0	OUT: 5	
998	✓	✗	✗	✗	✓	?
	0	Unknown	0	0	OUT: 0	
986	✓	✗	✗	✗	✓	✓
	0	Cytoplasmic	0	0	OUT: 0	
987	✗	✗	✓	✓	✓	✓
	13	CytoplasmicMembrane	5	3	OUT: 0	
984	✗	✗	✓	✗	✓	✓
	23	Cytoplasmic	2	0	OUT: 0	
985	✓	✗	✓	✗	✓	?
	0	CytoplasmicMembrane	1	0	OUT: 1	
990	✓	✗	✗	✗	✓	✗
	0	Cytoplasmic	0	0	OUT: 0	
991	✓	✗	✗	✓	✗	?
	0	Unknown	0	3	OUT: 2	
988	✓	✗	✗	✗	✓	✓
	0	Cytoplasmic	0	0	OUT: 0	
	✓	✗	✓	✓	✓	✓

989	0	Cytoplasmic	4	2	IN: 1	
978						
	0	Unknown	0	9	IN: 1	
979						
	0	Cytoplasmic	0	0	OUT: 2	
976						
	0	OuterMembrane	0	14	OUT: 0	
977						
	0	Cytoplasmic	1	0	OUT: 0	
982						
	0	Extracellular	1	2	OUT: 0	
983						
	0	Cytoplasmic	0	0	OUT: 0	
980						
	0	CytoplasmicMembrane	4	0	OUT: 1	
981						
	0	Unknown	0	0	OUT: 0	
971						
	0	OuterMembrane	0	2	OUT: 0	
970						

	0	Unknown	0	0	IN: 1	
969	✓	✗	✗	✓	✓	✓
	0	Periplasmic	0	7	OUT: 0	
968	✓	✗	✗	✗	✗	?
	0	CytoplasmicMembrane	0	0	IN: 2	
975	✓	✗	✓	✗	✗	✓
	0	Cytoplasmic	2	0	OUT: 2	
974	✓	✓	✗	✓	✓	✓
	0	Extracellular	0	14	OUT: 0	
973	✓	✗	✓	✗	✓	✓
	0	Cytoplasmic	2	0	OUT: 1	
972	✓	✗	✗	✗	✓	?
	0	Cytoplasmic	0	0	OUT: 0	
963	✓	✗	✗	✓	✓	✓
	0	CytoplasmicMembrane	0	3	IN: 1	
962	✓	✗	✓	✗	✓	✓
	0	Cytoplasmic	2	0	OUT: 0	
961	✓	✗	✗	✓	✗	✓
	0	CytoplasmicMem	0	4	OUT: 3	

		brane				
960	✓	✗	✗	✗	✗	?
	0	CytoplasmicMem brane	0	0	OUT: 5	
967	✓	✗	✗	✗	✗	✓
	0	CytoplasmicMem brane	0	0	IN: 6	
966	✓	✗	✓	✓	✓	✓
	0	CytoplasmicMem brane	28	35	OUT: 0	
965	✓	✗	✓	✗	✓	✓
	0	Cytoplasmic	1	0	IN: 0	
964	✓	✗	✗	✓	✓	✗
	0	Unknown	0	15	OUT: 0	
1100	✓	✗	✗	✗	✓	?
	0	CytoplasmicMem brane	0	0	OUT: 0	
1101	✗	✗	✗	✗	✓	✓
	30	Cytoplasmic	0	0	OUT: 0	
1102	✓	✗	✓	✗	✓	✗
	0	Unknown	1	0	OUT: 0	
1103	✓	✗	✗	✗	✓	✗

	0	Cytoplasmic	0	0	OUT: 0	
1096	✔	✘	✔	✘	✘	✔
	0	Unknown	1	0	OUT: 2	
1097	✔	✘	✘	✘	✔	✔
	0	Cytoplasmic	0	0	OUT: 0	
1098	✔	✘	✘	✘	✔	✔
	0	Unknown	0	0	OUT: 1	
1099	✔	✘	✘	✘	✔	✔
	0	Cytoplasmic	0	0	OUT: 0	
1092	✔	✔	✘	✘	✔	?
	0	Extracellular	0	0	OUT: 0	
1093	✔	✘	✘	✘	✘	✘
	0	CytoplasmicMem brane	0	0	IN: 3	
1094	✔	✘	✘	✘	✔	✔
	0	Periplasmic	0	0	OUT: 1	
1095	✔	✘	✘	✘	✘	✘
	0	Unknown	0	0	OUT: 3	
1088	✔	✘	✘	✘	✔	✔
	0	CytoplasmicMem brane	0	0	OUT: 0	

1089	✓	✗	✗	✗	✗	✗
	0	CytoplasmicMembrane	0	0	IN: 5	
1090	✓	✗	✗	✗	✓	?
	0	Unknown	0	0	IN: 0	
1091	✓	✗	✗	✗	✓	?
	0	Unknown	0	0	OUT: 0	
1117	✓	✗	✓	✗	✓	✓
	0	Cytoplasmic	14	0	OUT: 0	
1116	✓	✗	✓	✓	✓	✓
	0	Cytoplasmic	1	2	OUT: 1	
1119	✓	✗	✓	✗	✓	✓
	0	Cytoplasmic	4	0	OUT: 0	
1118	✓	✗	✗	✗	✓	✗
	0	Unknown	0	0	OUT: 0	
1113	✓	✗	✗	✗	✓	✗
	0	Unknown	0	0	OUT: 0	
1112	✓	✗	✓	✗	✓	✓
	0	CytoplasmicMembrane	2	0	IN: 1	
1115	✓	✗	✓	✗	✗	✓

	0	Cytoplasmic	1	0	IN: 6	
1114	✔	✘	✘	✘	✘	?
	0	Cytoplasmic	0	0	OUT: 2	
1109	✔	✘	✘	✘	✔	?
	0	Cytoplasmic	0	0	IN: 1	
1108	✔	✘	✔	✘	✔	?
	0	Cytoplasmic	1	0	OUT: 0	
1111	✔	✘	✘	✘	✘	✔
	0	CytoplasmicMembrane	0	0	OUT: 6	
1110	✔	✘	✘	✘	✘	✘
	0	CytoplasmicMembrane	0	0	IN: 3	
1105	✔	✘	✘	✘	✔	?
	0	Cytoplasmic	0	0	OUT: 0	
1104	✔	✘	✔	✘	✔	✔
	0	Cytoplasmic	2	0	OUT: 0	
1107	✘	✘	✔	✘	✔	✔
	43	Cytoplasmic	16	0	OUT: 0	
1106	✔	✘	✔	✘	✔	?
	0	Cytoplasmic	1	0	OUT: 0	

1134	✓	✗	✗	✗	✓	✓
	0	Cytoplasmic	0	0	OUT: 0	
1135	✓	✗	✓	✓	✓	✓
	0	Cytoplasmic	2	2	OUT: 0	
1132	✓	✗	✗	✗	✓	?
	0	Cytoplasmic	0	0	OUT: 0	
1133	✓	✗	✗	✗	✗	✗
	0	CytoplasmicMembrane	0	0	IN: 2	
1130	✓	✗	✓	✗	✗	✓
	0	CytoplasmicMembrane	1	0	IN: 4	
1131	✓	✗	✗	✗	✓	✓
	0	Unknown	0	0	OUT: 0	
1128	✓	✗	✓	✗	✓	✓
	0	Cytoplasmic	2	0	OUT: 0	
1129	✓	✗	✗	✗	✓	✓
	0	CytoplasmicMembrane	0	0	IN: 1	
1126	✓	✗	✗	✗	✓	✗
	0	Unknown	0	0	IN: 0	
	✓	✗	✗	✗	✗	✓

1127	0	CytoplasmicMembrane	0	0	OUT: 5	
1124	✓	✗	✗	✗	✗	✗
	0	CytoplasmicMembrane	0	0	OUT: 3	
1125	✓	✗	✗	✗	✓	✗
	0	Cytoplasmic	0	0	OUT: 0	
1122	✓	✗	✗	✗	✓	✗
	0	Unknown	0	0	OUT: 0	
1123	✓	✗	✓	✗	✓	✗
	0	Cytoplasmic	1	0	IN: 0	
1120	✓	✗	✓	✗	✓	✓
	0	Unknown	1	0	IN: 0	
1121	✓	✗	✗	✗	✓	✓
	0	Cytoplasmic	0	0	OUT: 0	
1151	✓	✗	✗	✗	✓	?
	0	Cytoplasmic	0	0	OUT: 0	
1150	✓	✗	✓	✗	✓	✓
	0	Cytoplasmic	1	0	OUT: 0	
1149	✓	✗	✓	✗	✗	✓
	0	Cytoplasmic	1	0	OUT: 3	

1148	✓	✗	✗	✗	✗	✓
	0	CytoplasmicMembrane	0	0	IN: 12	
1147	✓	✗	✗	✗	✓	?
	0	Cytoplasmic	0	0	OUT: 0	
1146	✓	✗	✓	✗	✓	✓
	0	CytoplasmicMembrane	11	0	OUT: 0	
1145	✓	✗	✓	✗	✓	?
	0	Periplasmic	1	0	IN: 1	
1144	✓	✗	✓	✗	✓	?
	0	Cytoplasmic	1	0	IN: 1	
1143	✓	✗	✓	✗	✓	✓
	0	Cytoplasmic	1	0	IN: 0	
1142	✓	✗	✓	✗	✓	✓
	0	Cytoplasmic	3	0	IN: 1	
1141	✓	✗	✗	✓	✓	✗
	0	Unknown	0	3	OUT: 0	
1140	✓	✗	✗	✗	✓	?
	0	Unknown	0	0	OUT: 0	
1139	✓	✗	✗	✗	✓	✗

	0	Cytoplasmic	0	0	OUT: 0	
1138	✓	✗	✓	✗	✗	✓
	0	Unknown	1	0	OUT: 2	
1137	✓	✗	✓	✗	✓	✓
	0	Cytoplasmic	4	0	OUT: 0	
1136	✗	✗	✓	✗	✓	✓
	1	Cytoplasmic	1	0	OUT: 1	
1032	✓	✗	✗	✗	✓	✗
	0	Cytoplasmic	0	0	OUT: 0	
1033	✓	✗	✗	✗	✓	?
	0	CytoplasmicMembrane	0	0	IN: 1	
1034	✓	✗	✗	✗	✓	?
	0	Cytoplasmic	0	0	OUT: 1	
1035	✓	✗	✓	✓	✓	?
	0	Cytoplasmic	1	2	IN: 1	
1036	✓	✗	✗	✗	✓	✗
	0	Cytoplasmic	0	0	OUT: 0	
1037	✓	✗	✗	✗	✓	✓
	0	Cytoplasmic	0	0	OUT: 0	
1038	✓	✗	✓	✗	✓	✓

	0	Cytoplasmic	25	0	OUT: 1	
1039	✔	✘	✘	✘	✔	✘
	0	Unknown	0	0	OUT: 0	
1024	✔	✘	✘	✔	✔	✔
	0	Cytoplasmic	0	2	OUT: 0	
1025	✔	✘	✘	✘	✔	✘
	0	Cytoplasmic	0	0	OUT: 0	
1026	✔	✘	✘	✘	✔	?
	0	Unknown	0	0	OUT: 0	
1027	✔	✘	✘	✘	✘	?
	0	CytoplasmicMembrane	0	0	OUT: 2	
1028	✔	✘	✘	✘	✔	✔
	0	CytoplasmicMembrane	0	0	IN: 1	
1029	✔	✘	✔	✘	✔	✔
	0	Cytoplasmic	1	0	OUT: 0	
1030	✔	✘	✘	✘	✘	✔
	0	CytoplasmicMembrane	0	0	OUT: 2	
1031	✔	✘	✔	✘	✔	✔

	0	Cytoplasmic	10	0	OUT: 1	
1049	✓	✗	✓	✗	✓	✓
	0	Cytoplasmic	1	0	OUT: 0	
1048	✓	✗	✓	✗	✗	✗
	0	CytoplasmicMembrane	1	0	IN: 4	
1051	✓	✗	✓	✗	✓	?
	0	Cytoplasmic	1	0	IN: 0	
1050	✓	✗	✓	✓	✓	✓
	0	Cytoplasmic	1	1	OUT: 0	
1053	✓	✗	✗	✓	✓	?
	0	CytoplasmicMembrane	0	3	OUT: 0	
1052	✓	✗	✗	✓	✗	✓
	0	CytoplasmicMembrane	0	4	OUT: 2	
1055	✓	✗	✗	✗	✓	✓
	0	Cytoplasmic	0	0	OUT: 0	
1054	✓	✗	✗	✗	✓	?
	0	Cytoplasmic	0	0	OUT: 0	
1041	✓	✗	✓	✗	✓	✓

	0	CytoplasmicMembrane	10	0	OUT: 0	
1040	✓	✗	✓	✗	✓	✓
	0	Cytoplasmic	1	0	OUT: 0	
1043	✓	✗	✗	✗	✓	✗
	0	Unknown	0	0	IN: 1	
1042	✓	✗	✗	✗	✓	✗
	0	Unknown	0	0	OUT: 0	
1045	✓	✗	✗	✗	✓	?
	0	Cytoplasmic	0	0	IN: 1	
1044	✓	✗	✗	✗	✓	✗
	0	Unknown	0	0	OUT: 1	
1047	✓	✗	✗	✗	✗	✓
	0	CytoplasmicMembrane	0	0	OUT: 3	
1046	✓	✗	✓	✓	✓	✓
	0	Cytoplasmic	6	23	OUT: 0	
1066	✓	✗	✗	✗	✓	✗
	0	Unknown	0	0	OUT: 0	
1067	✓	✗	✓	✗	✓	✓
	0	Cytoplasmic	14	0	OUT: 0	

1064	✓	✗	✗	✗	✓	✓
	0	Cytoplasmic	0	0	OUT: 0	
1065	✓	✗	✗	✓	✓	?
	0	Cytoplasmic	0	4	OUT: 0	
1070	✓	✗	✗	✗	✓	✓
	0	Cytoplasmic	0	0	IN: 1	
1071	✓	✗	✓	✗	✓	✓
	0	CytoplasmicMembrane	1	0	OUT: 0	
1068	✓	✗	✓	✗	✓	?
	0	Unknown	1	0	OUT: 0	
1069	✓	✗	✗	✓	✓	?
	0	Cytoplasmic	0	2	IN: 1	
1058	✓	✗	✓	✓	✓	✓
	0	Cytoplasmic	5	3	IN: 0	
1059	✓	✗	✓	✗	✓	✓
	0	Cytoplasmic	13	0	IN: 1	
1056	✓	✗	✗	✓	✓	✓
	0	Cytoplasmic	0	1	OUT: 0	
1057	✓	✓	✗	✗	✓	?
	0	OuterMembrane	0	0	OUT: 0	

1062	✓	✗	✓	✗	✓	✓
	0	Unknown	6	0	OUT: 0	
1063	✓	✗	✓	✗	✓	✓
	0	Cytoplasmic	1	0	OUT: 0	
1060	✓	✗	✗	✗	✗	✓
	0	Unknown	0	0	IN: 3	
1061	✓	✗	✓	✗	✓	✓
	0	Cytoplasmic	25	0	OUT: 0	
1083	✓	✗	✗	✗	✓	✓
	0	Cytoplasmic	0	0	IN: 0	
1082	✓	✗	✓	✗	✓	✗
	0	Cytoplasmic	1	0	IN: 1	
1081	✗	✗	✓	✗	✓	✓
	2	Cytoplasmic	17	0	OUT: 0	
1080	✓	✗	✓	✗	✓	✓
	0	Cytoplasmic	1	0	OUT: 0	
1087	✗	✗	✓	✓	✓	✓
	14	Cytoplasmic	13	14	IN: 0	
1086	✓	✗	✗	✗	✓	?
	0	Unknown	0	0	OUT: 0	

1085	✔	✖	✖	✖	✖	?
	0	CytoplasmicMembrane	0	0	OUT: 6	
1084	✔	✖	✖	✖	✔	✔
	0	Unknown	0	0	IN: 1	
1075	✔	✖	✔	✖	✔	✔
	0	Cytoplasmic	7	0	OUT: 0	
1074	✖	✖	✔	✔	✔	✔
	14	Cytoplasmic	17	2	OUT: 0	
1073	✔	✖	✖	✖	✔	✖
	0	Unknown	0	0	IN: 0	
1072	✔	✖	✖	✖	✔	✔
	0	Cytoplasmic	0	0	OUT: 0	
1079	✔	✖	✖	✖	✔	✔
	0	Cytoplasmic	0	0	OUT: 0	
1078	✔	✖	✖	✖	✔	?
	0	Periplasmic	0	0	IN: 1	
1077	✖	✖	✔	✖	✖	✔
	209	CytoplasmicMembrane	21	0	IN: 2	
1076	✖	✖	✔	✔	✖	✔

	8	CytoplasmicMembrane	21	1	IN: 4	
1221						
	0	CytoplasmicMembrane	0	0	IN: 6	
1220						
	6	CytoplasmicMembrane	6	3	OUT: 0	
1223						
	0	CytoplasmicMembrane	0	0	OUT: 4	
1222						
	0	Cytoplasmic	14	0	OUT: 0	
1217						
	0	Cytoplasmic	0	0	OUT: 0	
1216						
	0	Extracellular	10	0	IN: 1	
1219						
	0	Cytoplasmic	0	0	OUT: 0	
1218						
	0	Cytoplasmic	0	0	IN: 1	
1229						

	0	CytoplasmicMembrane	0	0	OUT: 0	
1228	✓	✗	✗	✗	✓	✓
	0	Cytoplasmic	0	0	OUT: 0	
1231	✓	✗	✗	✗	✓	✓
	0	CytoplasmicMembrane	0	0	OUT: 0	
1230	✓	✗	✗	✗	✗	✓
	0	Cytoplasmic	0	0	OUT: 2	
1225	✗	✗	✓	✗	✓	✓
	12	Cytoplasmic	11	0	OUT: 0	
1224	✓	✗	✗	✗	✓	✓
	0	Cytoplasmic	0	0	OUT: 0	
1227	✓	✗	✗	✓	✗	✓
	0	Unknown	0	3	IN: 2	
1226	✓	✗	✗	✗	✓	?
	0	Unknown	0	0	OUT: 0	
1236	✓	✗	✗	✗	✓	?
	0	Unknown	0	0	OUT: 0	
1237	✓	✗	✗	✗	✓	✓
	0	CytoplasmicMem	0	0	OUT: 0	

		brane				
1238	✓	✗	✗	✗	✓	?
	0	Unknown	0	0	OUT: 0	
1239	✓	✗	✓	✗	✓	✗
	0	Cytoplasmic	1	0	OUT: 0	
1232	✓	✗	✓	✗	✗	✓
	0	CytoplasmicMem brane	1	0	IN: 10	
1233	✗	✗	✓	✗	✓	✓
	11	Cytoplasmic	28	0	OUT: 0	
1234	✓	✗	✓	✗	✓	✓
	0	Cytoplasmic	2	0	OUT: 0	
1235	✓	✗	✗	✗	✓	?
	0	Cytoplasmic	0	0	OUT: 0	
1244	✓	✗	✗	✗	✓	?
	0	Cytoplasmic	0	0	OUT: 0	
1245	✓	✗	✓	✗	✗	?
	0	CytoplasmicMem brane	1	0	IN: 6	
1246	✓	✗	✓	✗	✓	✓
	0	Cytoplasmic	2	0	OUT: 0	

1247	✓	✗	✗	✗	✓	?
	0	Cytoplasmic	0	0	OUT: 0	
1240	✓	✗	✗	✓	✗	✗
	0	Unknown	0	15	OUT: 2	
1241	✓	✗	✗	✗	✓	✗
	0	Unknown	0	0	OUT: 0	
1242	✓	✗	✓	✗	✗	✓
	0	Cytoplasmic	8	0	OUT: 2	
1243	✓	✗	✗	✗	✓	✓
	0	Unknown	0	0	OUT: 0	
1255	✓	✗	✓	✗	✓	✓
	0	Cytoplasmic	3	0	OUT: 0	
1254	✓	✗	✓	✗	✓	✓
	0	Cytoplasmic	3	0	IN: 0	
1253	✓	✗	✗	✗	✓	✓
	0	Cytoplasmic	0	0	IN: 0	
1252	✓	✗	✗	✗	✓	✗
	0	Unknown	0	0	IN: 1	
1251	✓	✗	✓	✗	✓	✓
	0	Cytoplasmic	20	0	IN: 0	

1250	✓	✗	✗	✓	✗	?
	0	CytoplasmicMembrane	0	5	OUT: 5	
1249	✓	✗	✓	✗	✓	✓
	0	Cytoplasmic	12	0	IN: 0	
1248	✓	✗	✗	✗	✓	?
	0	Unknown	0	0	OUT: 0	
1263	✓	✗	✗	✗	✗	?
	0	CytoplasmicMembrane	0	0	IN: 8	
1262	✓	✗	✗	✗	✓	✓
	0	Cytoplasmic	0	0	OUT: 0	
1261	✓	✗	✗	✗	✓	?
	0	Cytoplasmic	0	0	IN: 1	
1260	✗	✗	✓	✗	✗	✓
	10	Cytoplasmic	1	0	OUT: 3	
1259	✓	✗	✓	✗	✓	✓
	0	Cytoplasmic	8	0	OUT: 0	
1258	✓	✗	✓	✗	✗	✓
	0	Cytoplasmic	1	0	OUT: 2	
1257	✓	✗	✓	✗	✓	✓

	0	Cytoplasmic	25	0	OUT: 0	
1256	✓	✗	✗	✗	✓	✓
	0	Unknown	0	0	IN: 1	
1270	✓	✗	✗	✗	✗	?
	0	Unknown	0	0	OUT: 2	
1271	✓	✗	✗	✗	✓	?
	0	Cytoplasmic	0	0	OUT: 0	
1268	✗	✗	✓	✗	✓	✓
	5	Cytoplasmic	20	0	OUT: 1	
1269	✓	✗	✓	✓	✗	✓
	0	CytoplasmicMembrane	9	13	OUT: 5	
1266	✓	✗	✓	✗	✓	✓
	0	Cytoplasmic	3	0	OUT: 0	
1267	✓	✗	✓	✗	✓	✓
	0	Cytoplasmic	7	0	OUT: 0	
1264	✓	✗	✗	✗	✓	?
	0	Cytoplasmic	0	0	OUT: 0	
1265	✓	✗	✓	✗	✓	✗
	0	Cytoplasmic	1	0	OUT: 0	
1278	✓	✗	✗	✗	✓	✓

	0	Unknown	0	0	OUT: 1	
1279	✓	✗	✗	✗	✗	✓
	0	CytoplasmicMembrane	0	0	OUT: 2	
1276	✓	✗	✗	✗	✓	?
	0	CytoplasmicMembrane	0	0	OUT: 0	
1277	✓	✗	✗	✗	✓	✓
	0	Cytoplasmic	0	0	OUT: 0	
1274	✓	✗	✓	✓	✓	✓
	0	Cytoplasmic	7	2	OUT: 1	
1275	✓	✗	✗	✗	✓	?
	0	Cytoplasmic	0	0	OUT: 0	
1272	✓	✗	✗	✗	✓	✗
	0	CytoplasmicMembrane	0	0	OUT: 0	
1273	✓	✗	✓	✗	✓	✓
	0	Unknown	5	0	OUT: 1	
1153	✓	✗	✓	✗	✓	✓
	0	Cytoplasmic	2	0	OUT: 0	
1152	✗	✗	✓	✗	✓	✓

	25	Cytoplasmic	28	0	OUT: 0	
1155	✓	✗	✓	✗	✓	?
	0	Cytoplasmic	1	0	OUT: 0	
1154	✓	✗	✓	✗	✓	✓
	0	Unknown	1	0	OUT: 0	
1157	✓	✗	✗	✗	✓	✓
	0	Cytoplasmic	0	0	OUT: 0	
1156	✓	✗	✗	✗	✗	✓
	0	CytoplasmicMembrane	0	0	OUT: 6	
1159	✓	✗	✗	✓	✓	?
	0	Cytoplasmic	0	3	IN: 1	
1158	✓	✗	✓	✗	✓	✓
	0	Cytoplasmic	1	0	OUT: 0	
1161	✓	✗	✓	✗	✓	✓
	0	Cytoplasmic	17	0	OUT: 0	
1160	✓	✗	✓	✓	✗	✓
	0	CytoplasmicMembrane	2	1	IN: 9	
1163	✓	✗	✗	✗	✓	?
	0	Cytoplasmic	0	0	OUT: 0	

1162	✔	✖	✔	✔	✔	✔
	0	Cytoplasmic	2	2	OUT: 0	
1165	✔	✖	✔	✖	✔	?
	0	Unknown	1	0	OUT: 0	
1164	✔	✖	✖	✔	✖	✔
	0	CytoplasmicMembrane	0	5	IN: 3	
1167	✖	✖	✔	✖	✔	✔
	10	Cytoplasmic	3	0	OUT: 0	
1166	✔	✖	✔	✖	✔	?
	0	Cytoplasmic	1	0	IN: 0	
1168	✔	✖	✖	✖	✖	?
	0	CytoplasmicMembrane	0	0	OUT: 6	
1169	✖	✖	✔	✔	✔	✔
	2	Cytoplasmic	18	17	OUT: 0	
1170	✔	✖	✖	✖	✔	?
	0	Cytoplasmic	0	0	IN: 1	
1171	✖	✖	✔	✖	✔	✔
	7	Cytoplasmic	3	0	OUT: 0	
1172	✔	✖	✔	✖	✖	✔

	0	Cytoplasmic	19	0	OUT: 2	
1173	✓	✗	✓	✓	✗	✓
	0	CytoplasmicMem brane	1	1	OUT: 2	
1174	✓	✗	✗	✗	✓	✗
	0	Unknown	0	0	IN: 1	
1175	✗	✓	✓	✗	✓	✓
	22	Extracellular	8	0	IN: 0	
1176	✗	✗	✓	✓	✓	✓
	23	Unknown	6	2	OUT: 0	
1177	✗	✗	✓	✗	✓	✓
	3	Cytoplasmic	7	0	OUT: 0	
1178	✓	✗	✓	✗	✓	✓
	0	Unknown	1	0	OUT: 0	
1179	✓	✗	✗	✗	✗	✗
	0	CytoplasmicMem brane	0	0	IN: 2	
1180	✓	✗	✗	✓	✗	✗
	0	Unknown	0	3	IN: 3	
1181	✓	✗	✗	✗	✓	✗
	0	Unknown	0	0	IN: 0	

1182	✔	✘	✘	✘	✔	✘
	0	Unknown	0	0	OUT: 0	
1183	✔	✘	✘	✔	✔	✔
	0	Cytoplasmic	0	1	OUT: 1	
1187	✔	✘	✔	✘	✘	✔
	0	Unknown	3	0	OUT: 2	
1186	✔	✘	✔	✘	✔	✔
	0	Cytoplasmic	22	0	OUT: 1	
1185	✔	✘	✔	✘	✔	✔
	0	Cytoplasmic	2	0	OUT: 0	
1184	✔	✔	✔	✔	✔	?
	0	OuterMembrane	1	1	OUT: 0	
1191	✔	✘	✘	✘	✔	✔
	0	Cytoplasmic	0	0	OUT: 0	
1190	✔	✘	✘	✘	✔	✔
	0	Unknown	0	0	OUT: 0	
1189	✔	✘	✔	✘	✔	✘
	0	Unknown	1	0	IN: 1	
1188	✔	✘	✔	✘	✔	✔
	0	Cytoplasmic	8	0	OUT: 1	

1195	✔	✘	✘	✘	✔	✔
	0	Cytoplasmic	0	0	OUT: 0	
1194	✔	✘	✔	✘	✔	✔
	0	Cytoplasmic	1	0	OUT: 0	
1193	✔	✘	✔	✘	✔	✔
	0	Cytoplasmic	17	0	OUT: 0	
1192	✔	✘	✘	✘	✔	✘
	0	Unknown	0	0	OUT: 0	
1199	✔	✘	✘	✘	✔	?
	0	Cytoplasmic	0	0	OUT: 0	
1198	✔	✘	✘	✘	✘	✘
	0	CytoplasmicMem brane	0	0	OUT: 4	
1197	✔	✘	✔	✘	✔	✔
	0	Cytoplasmic	8	0	OUT: 0	
1196	✔	✘	✔	✘	✔	✔
	0	Cytoplasmic	1	0	IN: 0	
1202	✔	✔	✘	✘	✔	?
	0	OuterMembrane	0	0	IN: 1	
1203	✔	✘	✔	✘	✘	✔
	0	CytoplasmicMem	2	0	OUT: 7	

		brane				
1200	✔	✘	✘	✘	✔	?
	0	Cytoplasmic	0	0	OUT: 0	
1201	✔	✘	✘	✘	✔	✔
	0	Cytoplasmic	0	0	IN: 1	
1206	✔	✘	✘	✔	✔	✔
	0	Cytoplasmic	0	1	IN: 0	
1207	✔	✘	✘	✘	✔	✔
	0	Cytoplasmic	0	0	OUT: 0	
1204	✔	✔	✔	✘	✔	✔
	0	Extracellular	1	0	OUT: 0	
1205	✔	✘	✘	✘	✔	?
	0	Unknown	0	0	IN: 1	
1210	✔	✘	✘	✘	✔	✘
	0	Unknown	0	0	OUT: 1	
1211	✔	✘	✘	✔	✘	?
	0	Cytoplasmic	0	3	IN: 3	
1208	✔	✔	✘	✔	✔	?
	0	OuterMembrane	0	4	OUT: 0	
1209	✔	✘	✔	✔	✘	✔

	0	CytoplasmicMembrane	1	1	OUT: 7	
1214	✓	✗	✓	✗	✗	?
	0	Cytoplasmic	2	0	IN: 3	
1215	✓	✗	✗	✗	✗	?
	0	CytoplasmicMembrane	0	0	OUT: 11	
1212	✓	✗	✗	✗	✓	✗
	0	Unknown	0	0	OUT: 0	
1213	✓	✗	✗	✗	✗	✗
	0	CytoplasmicMembrane	0	0	OUT: 2	
1375	✓	✗	✓	✗	✓	✗
	0	Unknown	1	0	OUT: 0	
1374	✓	✗	✓	✗	✓	✗
	0	Unknown	1	0	OUT: 0	
1373	✓	✗	✗	✗	✓	?
	0	Unknown	0	0	OUT: 0	
1372	✓	✗	✗	✗	✗	?
	0	CytoplasmicMembrane	0	0	IN: 6	
1371	✓	✗	✗	✗	✓	✓

	0	Cytoplasmic	0	0	OUT: 0	
1370	✔	✘	✔	✘	✔	✔
	0	Cytoplasmic	25	0	IN: 0	
1369	✔	✘	✘	✘	✔	✘
	0	Cytoplasmic	0	0	OUT: 0	
1368	✔	✘	✘	✘	✔	✔
	0	Unknown	0	0	OUT: 0	
1367	✔	✘	✘	✘	✔	✘
	0	Unknown	0	0	IN: 1	
1366	✔	✘	✘	✘	✔	✔
	0	Cytoplasmic	0	0	IN: 0	
1365	✔	✘	✘	✘	✔	?
	0	Cytoplasmic	0	0	OUT: 0	
1364	✔	✘	✔	✘	✔	✔
	0	Cytoplasmic	8	0	OUT: 1	
1363	✔	✘	✘	✘	✔	✔
	0	Cytoplasmic	0	0	OUT: 0	
1362	✔	✘	✘	✘	✔	✘
	0	Unknown	0	0	IN: 1	
1361	✔	✘	✘	✘	✘	?

	0	CytoplasmicMembrane	0	0	OUT: 7	
1360	✔	✘	✘	✘	✔	?
	0	Cytoplasmic	0	0	OUT: 0	
1358	✔	✘	✔	✔	✔	✔
	0	Cytoplasmic	12	4	OUT: 0	
1359	✔	✔	✔	✔	✔	?
	0	OuterMembrane	1	3	IN: 1	
1356	✔	✘	✔	✔	✔	✔
	0	Cytoplasmic	1	4	OUT: 0	
1357	✔	✘	✔	✘	✔	✔
	0	Cytoplasmic	11	0	OUT: 0	
1354	✔	✔	✔	✘	✔	?
	0	OuterMembrane	1	0	IN: 1	
1355	✔	✘	✘	✘	✔	✔
	0	Unknown	0	0	OUT: 0	
1352	✔	✘	✘	✘	✔	?
	0	Unknown	0	0	IN: 1	
1353	✘	✘	✘	✘	✔	✔
	13	Cytoplasmic	0	0	OUT: 0	
1350	✔	✘	✔	✘	✘	?

	0	CytoplasmicMembrane	1	0	OUT: 6	
1351	✓	✗	✓	✗	✗	✓
	0	Cytoplasmic	17	0	OUT: 2	
1348	✓	✗	✓	✗	✗	✓
	0	CytoplasmicMembrane	1	0	IN: 2	
1349	✓	✗	✓	✗	✓	✓
	0	Cytoplasmic	4	0	OUT: 0	
1346	✓	✗	✗	✗	✗	?
	0	CytoplasmicMembrane	0	0	IN: 5	
1347	✓	✗	✓	✗	✗	?
	0	CytoplasmicMembrane	1	0	IN: 4	
1344	✓	✗	✓	✗	✓	✓
	0	Unknown	1	0	OUT: 0	
1345	✓	✗	✗	✗	✓	?
	0	Unknown	0	0	OUT: 0	
1405	✓	✗	✗	✗	✗	?
	0	CytoplasmicMembrane	0	0	OUT: 8	

1404	0	Cytoplasmic	25	0	IN: 0	
1407	✓	✗	✗	✗	✓	?
	0	Cytoplasmic	0	0	OUT: 0	
1406	✗	✗	✗	✗	✓	✓
	8	Cytoplasmic	0	0	IN: 1	
1401	✓	✗	✗	✓	✓	?
	0	CytoplasmicMembrane	0	3	OUT: 0	
1400	✓	✗	✓	✗	✓	?
	0	Cytoplasmic	1	0	OUT: 0	
1403	✓	✗	✓	✗	✓	?
	0	Cytoplasmic	1	0	OUT: 0	
1402	✓	✗	✗	✗	✓	?
	0	Cytoplasmic	0	0	OUT: 0	
1397	✗	✗	✓	✗	✓	✓
	35	Cytoplasmic	1	0	OUT: 0	
1396	✓	✗	✓	✓	✓	✓
	0	CytoplasmicMembrane	25	9	OUT: 0	
1399	✓	✗	✓	✗	✓	✓
	0	Cytoplasmic	1	0	OUT: 0	

1398	✔	✘	✘	✘	✔	✘
	0	Unknown	0	0	OUT: 0	
1393	✔	✘	✘	✔	✔	?
	0	Unknown	0	15	OUT: 0	
1392	✔	✘	✘	✘	✔	✔
	0	Cytoplasmic	0	0	OUT: 0	
1395	✘	✘	✔	✔	✔	✔
	4	Cytoplasmic	6	1	OUT: 0	
1394	✔	✘	✔	✘	✔	?
	0	Cytoplasmic	1	0	OUT: 0	
1388	✔	✘	✘	✘	✔	?
	0	Cytoplasmic	0	0	OUT: 0	
1389	✔	✘	✔	✘	✔	✔
	0	Cytoplasmic	24	0	IN: 1	
1390	✔	✘	✔	✘	✔	✔
	0	Cytoplasmic	26	0	OUT: 0	
1391	✔	✘	✘	✘	✔	✔
	0	Cytoplasmic	0	0	OUT: 1	
1384	✔	✘	✔	✘	✔	✔
	0	Unknown	1	0	OUT: 0	

1385	✔	✘	✘	✘	✔	✘
	0	Cytoplasmic	0	0	IN: 0	
1386	✔	✔	✔	✘	✔	?
	0	OuterMembrane	1	0	OUT: 0	
1387	✔	✘	✔	✘	✔	✔
	0	Cytoplasmic	17	0	IN: 1	
1380	✔	✘	✘	✘	✘	?
	0	CytoplasmicMembrane	0	0	OUT: 2	
1381	✔	✘	✔	✘	✔	✔
	0	Cytoplasmic	11	0	OUT: 0	
1382	✔	✘	✘	✘	✘	?
	0	Unknown	0	0	OUT: 2	
1383	✔	✘	✘	✘	✘	✘
	0	CytoplasmicMembrane	0	0	IN: 4	
1376	✔	✘	✔	✘	✘	✔
	0	CytoplasmicMembrane	1	0	OUT: 11	
1377	✔	✘	✘	✘	✘	?
	0	CytoplasmicMembrane	0	0	OUT: 3	

1378	✓	✗	✗	✓	✗	✗
	0	Unknown	0	15	OUT: 2	
1379	✓	✗	✓	✓	✓	?
	0	CytoplasmicMembrane	1	6	IN: 1	
1307	✓	✗	✗	✗	✓	?
	0	Cytoplasmic	0	0	IN: 0	
1306	✓	✗	✗	✗	✓	✗
	0	Unknown	0	0	IN: 0	
1305	✓	✗	✗	✓	✓	✓
	0	Cytoplasmic	0	7	IN: 1	
1304	✓	✗	✗	✗	✗	✓
	0	Cytoplasmic	0	0	OUT: 2	
1311	✓	✗	✗	✗	✓	✓
	0	Cytoplasmic	0	0	OUT: 1	
1310	✓	✗	✗	✗	✓	✗
	0	Unknown	0	0	OUT: 0	
1309	✓	✗	✓	✗	✓	✓
	0	CytoplasmicMembrane	1	0	IN: 1	
1308	✓	✗	✗	✗	✓	✓

	0	Cytoplasmic	0	0	OUT: 0	
1299	✔	✘	✘	✔	✔	?
	0	CytoplasmicMembrane	0	7	OUT: 0	
1298	✔	✘	✘	✘	✔	?
	0	Cytoplasmic	0	0	OUT: 0	
1297	✔	✘	✔	✘	✔	✔
	0	Cytoplasmic	4	0	IN: 1	
1296	✔	✘	✔	✘	✔	✔
	0	Cytoplasmic	19	0	OUT: 1	
1303	✔	✘	✘	✘	✘	?
	0	Unknown	0	0	OUT: 2	
1302	✔	✘	✘	✘	✔	✘
	0	Unknown	0	0	OUT: 0	
1301	✔	✔	✔	✘	✘	✔
	0	OuterMembrane	1	0	OUT: 2	
1300	✔	✘	✔	✘	✔	✔
	0	Cytoplasmic	1	0	IN: 1	
1290	✔	✘	✘	✘	✘	✔
	0	Cytoplasmic	0	0	OUT: 2	
1291	✔	✘	✔	✘	✔	✔

	0	Cytoplasmic	3	0	IN: 1	
1288	✔	✘	✘	✘	✘	?
	0	CytoplasmicMembrane	0	0	OUT: 4	
1289	✔	✘	✘	✘	✔	✔
	0	Unknown	0	0	OUT: 0	
1294	✔	✘	✔	✘	✔	✔
	0	Unknown	1	0	OUT: 0	
1295	✘	✘	✔	✘	✔	✔
	17	Cytoplasmic	5	0	IN: 1	
1292	✔	✘	✘	✘	✘	✘
	0	Unknown	0	0	OUT: 2	
1293	✔	✘	✘	✘	✔	✔
	0	Unknown	0	0	IN: 1	
1282	✔	✘	✘	✔	✔	?
	0	Cytoplasmic	0	1	OUT: 0	
1283	✔	✘	✘	✔	✘	✔
	0	CytoplasmicMembrane	0	5	IN: 2	
1280	✔	✘	✔	✘	✔	✔
	0	Cytoplasmic	1	0	OUT: 0	

1281	✔	✘	✘	✘	✔	✔
	0	Cytoplasmic	0	0	OUT: 0	
1286	✔	✘	✘	✘	✘	✔
	0	Unknown	0	0	IN: 2	
1287	✔	✘	✘	✘	✔	✔
	0	Cytoplasmic	0	0	OUT: 0	
1284	✔	✘	✘	✘	✔	✔
	0	Cytoplasmic	0	0	OUT: 0	
1285	✔	✘	✘	✘	✔	?
	0	Cytoplasmic	0	0	OUT: 0	
1337	✔	✘	✔	✘	✔	✘
	0	Unknown	1	0	OUT: 0	
1336	✔	✘	✔	✘	✔	✔
	0	Cytoplasmic	6	0	OUT: 0	
1339	✔	✘	✘	✘	✘	?
	0	CytoplasmicMembrane	0	0	IN: 12	
1338	✔	✔	✘	✔	✔	✔
	0	Extracellular	0	4	OUT: 0	
1341	✔	✘	✘	✘	✘	✔
	0	CytoplasmicMem	0	0	OUT: 5	

		brane				
1340	✓	✗	✗	✓	✓	✓
	0	Cytoplasmic	0	2	OUT: 0	
1343	✓	✗	✗	✗	✓	✗
	0	CytoplasmicMembrane	0	0	IN: 1	
1342	✓	✗	✗	✗	✓	?
	0	Unknown	0	0	OUT: 0	
1329	✓	✗	✗	✗	✗	?
	0	CytoplasmicMembrane	0	0	OUT: 5	
1328	✓	✗	✗	✗	✓	?
	0	Unknown	0	0	OUT: 0	
1331	✓	✗	✓	✗	✓	✓
	0	Cytoplasmic	3	0	OUT: 0	
1330	✗	✗	✓	✓	✓	✓
	97	Cytoplasmic	25	6	IN: 0	
1333	✓	✗	✓	✗	✓	?
	0	Cytoplasmic	1	0	IN: 0	
1332	✓	✗	✗	✗	✓	✗
	0	Unknown	0	0	OUT: 0	

1335						
	0	Cytoplasmic	0	0	OUT: 0	
1334						
	0	Unknown	0	0	OUT: 0	
1320						
	0	CytoplasmicMem brane	0	0	OUT: 0	
1321						
	0	CytoplasmicMem brane	1	0	OUT: 4	
1322						
	0	Cytoplasmic	14	3	OUT: 0	
1323						
	0	Cytoplasmic	0	0	IN: 1	
1324						
	39	Cytoplasmic	25	1	OUT: 0	
1325						
	0	Cytoplasmic	3	0	OUT: 0	
1326						
	0	Unknown	0	0	IN: 1	
1327						

	0	Cytoplasmic	1	0	OUT: 0	
1312	✓	✗	✗	✗	✓	✗
	0	Cytoplasmic	0	0	OUT: 0	
1313	✓	✗	✗	✗	✓	✗
	0	Unknown	0	0	OUT: 0	
1314	✓	✗	✗	✗	✓	✓
	0	Cytoplasmic	0	0	OUT: 0	
1315	✓	✗	✓	✗	✓	✓
	0	Cytoplasmic	27	0	OUT: 1	
1316	✓	✗	✗	✗	✗	✗
	0	CytoplasmicMembrane	0	0	IN: 2	
1317	✓	✗	✓	✗	✓	✓
	0	Unknown	1	0	IN: 1	
1318	✓	✗	✗	✗	✓	✗
	0	Cytoplasmic	0	0	OUT: 0	
1319	✗	✗	✗	✓	✓	✓
	22	Cytoplasmic	0	5	OUT: 0	
1494	✓	✗	✓	✗	✓	✓
	0	Cytoplasmic	10	0	OUT: 0	
1495	✓	✗	✓	✗	✓	✓

	0	Cytoplasmic	2	0	OUT: 0	
1492	✓	✗	✓	✗	✓	✓
	0	Cytoplasmic	1	0	OUT: 0	
1493	✓	✗	✗	✗	✓	✓
	0	Cytoplasmic	0	0	OUT: 0	
1490	✓	✗	✗	✗	✓	?
	0	Cytoplasmic	0	0	OUT: 0	
1491	✗	✗	✓	✗	✓	✓
	10	Cytoplasmic	24	0	OUT: 0	
1488	✓	✗	✓	✗	✗	?
	0	Cytoplasmic	1	0	IN: 2	
1489	✓	✗	✗	✗	✓	?
	0	CytoplasmicMembrane	0	0	IN: 1	
1502	✓	✗	✓	✗	✓	✓
	0	Cytoplasmic	16	0	OUT: 1	
1503	✓	✗	✗	✗	✓	?
	0	Cytoplasmic	0	0	OUT: 0	
1500	✓	✗	✗	✗	✓	✗
	0	CytoplasmicMembrane	0	0	OUT: 1	

1501	✔	✘	✘	✘	✔	✘
	0	Unknown	0	0	IN: 0	
1498	✔	✘	✔	✘	✔	✔
	0	Cytoplasmic	2	0	IN: 0	
1499	✔	✘	✔	✘	✔	?
	0	Unknown	1	0	OUT: 0	
1496	✔	✘	✔	✘	✔	✔
	0	Cytoplasmic	3	0	OUT: 1	
1497	✘	✘	✔	✘	✔	✔
	8	Cytoplasmic	25	0	OUT: 0	
1479	✔	✘	✔	✘	✔	✔
	0	Cytoplasmic	5	0	OUT: 0	
1478	✔	✘	✔	✔	✔	✔
	0	CytoplasmicMem brane	1	1	OUT: 1	
1477	✔	✘	✘	✘	✔	?
	0	Cytoplasmic	0	0	OUT: 0	
1476	✔	✘	✘	✘	✔	✔
	0	Cytoplasmic	0	0	OUT: 1	
1475	✔	✘	✘	✘	✔	✘
	0	Cytoplasmic	0	0	OUT: 0	

1474	✓	✗	✗	✗	✓	✓
	0	Unknown	0	0	OUT: 0	
1473	✓	✗	✓	✓	✓	✓
	0	Unknown	1	3	OUT: 1	
1472	✓	✗	✗	✗	✓	✓
	0	Unknown	0	0	IN: 0	
1487	✓	✗	✗	✗	✓	✓
	0	Cytoplasmic	0	0	OUT: 0	
1486	✓	✗	✗	✗	✗	✓
	0	CytoplasmicMembrane	0	0	IN: 2	
1485	✓	✗	✗	✗	✗	?
	0	Cytoplasmic	0	0	OUT: 2	
1484	✓	✓	✗	✓	✓	?
	0	OuterMembrane	0	3	OUT: 0	
1483	✓	✗	✗	✗	✗	✗
	0	CytoplasmicMembrane	0	0	IN: 2	
1482	✓	✗	✓	✓	✓	✓
	0	CytoplasmicMembrane	3	19	IN: 1	
	✓	✗	✓	✗	✓	✓

1481	0	Cytoplasmic	2	0	OUT: 0	
1480	✔	✘	✔	✔	✘	✔
	0	CytoplasmicMembrane	2	2	OUT: 3	
1524	✔	✘	✘	✘	✔	?
	0	Unknown	0	0	OUT: 0	
1525	✔	✘	✔	✘	✘	✔
	0	Cytoplasmic	2	0	OUT: 2	
1526	✔	✘	✘	✘	✔	✔
	0	Cytoplasmic	0	0	OUT: 0	
1527	✔	✘	✘	✘	✔	?
	0	Cytoplasmic	0	0	OUT: 0	
1520	✔	✘	✘	✘	✔	?
	0	Unknown	0	0	OUT: 0	
1521	✔	✘	✔	✘	✔	✘
	0	Cytoplasmic	1	0	OUT: 0	
1522	✔	✘	✔	✘	✔	✔
	0	CytoplasmicMembrane	1	0	OUT: 1	
1523	✔	✘	✘	✘	✔	✘
	0	Unknown	0	0	OUT: 0	

1532	✓	✗	✗	✓	✓	?
	0	Unknown	0	3	OUT: 0	
1533	✓	✗	✗	✗	✓	?
	0	Unknown	0	0	OUT: 0	
1534	✓	✗	✓	✗	✓	?
	0	CytoplasmicMembrane	1	0	OUT: 1	
1535	✓	✓	✗	✓	✓	✓
	0	Extracellular	0	22	OUT: 0	
1528	✓	✗	✗	✗	✓	✓
	0	Cytoplasmic	0	0	OUT: 0	
1529	✓	✗	✗	✓	✓	✗
	0	Cytoplasmic	0	3	OUT: 0	
1530	✗	✗	✓	✗	✓	✓
	3	Cytoplasmic	17	0	OUT: 0	
1531	✓	✗	✗	✗	✗	✓
	0	Unknown	0	0	OUT: 2	
1509	✓	✗	✓	✗	✓	✓
	0	Unknown	1	0	IN: 0	
1508	✓	✗	✓	✗	✗	✓
	0	CytoplasmicMem	1	0	OUT: 8	

		brane				
1511	✔	✘	✘	✘	✘	?
	0	CytoplasmicMembrane	0	0	IN: 12	
1510	✔	✘	✔	✘	✘	?
	0	CytoplasmicMembrane	1	0	IN: 5	
1505	✔	✘	✘	✘	✔	✔
	0	Cytoplasmic	0	0	OUT: 0	
1504	✔	✘	✘	✘	✘	?
	0	CytoplasmicMembrane	0	0	OUT: 4	
1507	✔	✘	✘	✘	✔	✘
	0	Unknown	0	0	OUT: 0	
1506	✔	✘	✘	✘	✔	✔
	0	Unknown	0	0	IN: 0	
1517	✔	✘	✔	✘	✔	✔
	0	Unknown	1	0	OUT: 0	
1516	✔	✘	✘	✘	✔	✔
	0	Cytoplasmic	0	0	OUT: 0	
1519	✔	✘	✘	✘	✔	✘

	0	Unknown	0	0	OUT: 1	
1518	✔	✖	✔	✖	✔	?
	0	Cytoplasmic	1	0	OUT: 0	
1513	✖	✖	✔	✖	✔	✔
	4	Cytoplasmic	10	0	OUT: 0	
1512	✖	✖	✔	✖	✔	✔
	13	Cytoplasmic	14	0	IN: 0	
1515	✔	✖	✖	✖	✔	?
	0	Unknown	0	0	IN: 1	
1514	✖	✖	✔	✖	✔	✔
	4	Cytoplasmic	2	0	OUT: 0	
1426	✖	✖	✔	✖	✔	✔
	21	Cytoplasmic	17	0	OUT: 0	
1427	✔	✖	✔	✖	✔	✖
	0	Unknown	1	0	IN: 0	
1424	✔	✖	✖	✖	✔	✔
	0	Cytoplasmic	0	0	OUT: 0	
1425	✔	✖	✖	✖	✔	✔
	0	Cytoplasmic	0	0	OUT: 0	
1430	✔	✖	✖	✖	✔	✔

	0	Unknown	0	0	IN: 0	
1431	✔	✘	✘	✘	✔	?
	0	Cytoplasmic	0	0	OUT: 0	
1428	✔	✘	✘	✔	✔	✔
	0	Unknown	0	3	OUT: 1	
1429	✔	✘	✘	✘	✔	?
	0	Cytoplasmic	0	0	OUT: 0	
1434	✔	✘	✘	✔	✘	✔
	0	CytoplasmicMembrane	0	4	OUT: 6	
1435	✘	✘	✔	✔	✔	✔
	6	Cytoplasmic	4	1	OUT: 0	
1432	✔	✘	✘	✘	✔	✘
	0	Cytoplasmic	0	0	OUT: 0	
1433	✔	✘	✘	✘	✔	✔
	0	Unknown	0	0	IN: 1	
1438	✔	✘	✘	✘	✔	?
	0	Cytoplasmic	0	0	OUT: 0	
1439	✔	✘	✘	✘	✔	✔
	0	Cytoplasmic	0	0	OUT: 0	
1436	✔	✘	✔	✘	✘	✔

	0	CytoplasmicMembrane	1	0	OUT: 3	
1437	✔	✖	✖	✖	✔	?
	0	Unknown	0	0	OUT: 0	
1411	✔	✖	✖	✖	✖	?
	0	CytoplasmicMembrane	0	0	IN: 5	
1410	✔	✖	✖	✖	✔	?
	0	Unknown	0	0	IN: 1	
1409	✔	✖	✖	✖	✔	✔
	0	Cytoplasmic	0	0	OUT: 0	
1408	✔	✖	✔	✖	✔	✔
	0	Cytoplasmic	1	0	OUT: 0	
1415	✔	✖	✔	✖	✔	✔
	0	Cytoplasmic	1	0	OUT: 0	
1414	✔	✖	✖	✖	✖	✖
	0	CytoplasmicMembrane	0	0	IN: 3	
1413	✔	✖	✔	✔	✖	✔
	0	CytoplasmicMembrane	2	2	OUT: 3	
1412	✔	✖	✖	✖	✔	?

	0	Cytoplasmic	0	0	IN: 0	
1419	✓	✗	✓	✗	✓	✓
	0	Cytoplasmic	5	0	IN: 0	
1418	✓	✗	✓	✓	✗	✓
	0	CytoplasmicMembrane	1	3	IN: 3	
1417	✓	✗	✗	✗	✗	?
	0	Cytoplasmic	0	0	OUT: 2	
1416	✓	✗	✗	✗	✓	✗
	0	Unknown	0	0	OUT: 0	
1423	✓	✗	✗	✗	✓	✓
	0	Unknown	0	0	IN: 1	
1422	✗	✗	✓	✓	✓	✓
	2	Cytoplasmic	13	1	OUT: 0	
1421	✓	✗	✗	✗	✓	✗
	0	Cytoplasmic	0	0	OUT: 0	
1420	✓	✗	✗	✗	✓	✓
	0	Cytoplasmic	0	0	IN: 0	
1456	✓	✗	✗	✗	✗	?
	0	CytoplasmicMembrane	0	0	IN: 10	

1457	✔	✘	✔	✘	✔	✔
	0	Cytoplasmic	1	0	IN: 0	
1458	✔	✘	✔	✘	✔	?
	0	Cytoplasmic	1	0	OUT: 0	
1459	✔	✘	✘	✘	✔	✘
	0	Unknown	0	0	IN: 1	
1460	✔	✘	✘	✔	✔	✔
	0	Cytoplasmic	0	3	OUT: 0	
1461	✔	✘	✘	✘	✔	✔
	0	Unknown	0	0	OUT: 0	
1462	✔	✘	✔	✘	✔	✔
	0	Cytoplasmic	1	0	OUT: 0	
1463	✔	✘	✘	✘	✔	?
	0	Unknown	0	0	OUT: 0	
1464	✔	✘	✘	✔	✔	?
	0	Cytoplasmic	0	5	IN: 0	
1465	✔	✘	✔	✘	✘	✔
	0	CytoplasmicMembrane	1	0	IN: 8	
1466	✔	✘	✘	✘	✘	✘
	0	CytoplasmicMem	0	0	IN: 2	

		brane				
1467	✔	✔	✘	✔	✔	✘
	0	OuterMembrane	0	15	IN: 1	
1468	✔	✘	✘	✘	✘	✔
	0	Cytoplasmic	0	0	OUT: 2	
1469	✔	✘	✘	✘	✔	✔
	0	Cytoplasmic	0	0	OUT: 1	
1470	✔	✘	✔	✘	✔	✔
	0	Cytoplasmic	17	0	OUT: 0	
1471	✔	✘	✘	✘	✔	✘
	0	Unknown	0	0	OUT: 0	
1441	✔	✘	✔	✘	✘	✔
	0	CytoplasmicMem brane	1	0	IN: 9	
1440	✘	✘	✔	✘	✔	✔
	10	Cytoplasmic	9	0	OUT: 0	
1443	✔	✘	✘	✘	✔	?
	0	Cytoplasmic	0	0	OUT: 0	
1442	✔	✘	✔	✘	✘	?
	0	CytoplasmicMem brane	1	0	OUT: 6	

1445	✓	✗	✗	✗	✓	✗
	0	Unknown	0	0	OUT: 0	
1444	✓	✗	✓	✗	✗	✓
	0	Cytoplasmic	9	0	OUT: 3	
1447	✓	✗	✗	✗	✓	✗
	0	Unknown	0	0	IN: 1	
1446	✓	✗	✓	✗	✓	✓
	0	Cytoplasmic	1	0	OUT: 0	
1449	✓	✗	✓	✗	✓	✓
	0	Cytoplasmic	40	0	OUT: 0	
1448	✓	✗	✗	✗	✓	✗
	0	CytoplasmicMembrane	0	0	OUT: 0	
1451	✓	✗	✗	✗	✓	✗
	0	Unknown	0	0	IN: 0	
1450	✓	✗	✗	✗	✓	✓
	0	Unknown	0	0	OUT: 0	
1453	✓	✗	✓	✗	✓	✓
	0	Cytoplasmic	2	0	OUT: 0	
1452	✓	✗	✗	✗	✓	?
	0	Cytoplasmic	0	0	IN: 0	

1455	✓	✗	✗	✗	✓	✓
	0	Cytoplasmic	0	0	IN: 0	
1454	✓	✗	✗	✗	✓	✗
	0	Unknown	0	0	IN: 1	
1576	✓	✗	✗	✗	✓	✓
	0	Unknown	0	0	OUT: 1	
1574	✓	✗	✓	✗	✗	✓
	0	CytoplasmicMembrane	1	0	IN: 3	
1575	✓	✗	✓	✗	✓	✓
	0	Unknown	17	0	OUT: 1	
1572	✓	✗	✗	✗	✓	✓
	0	Cytoplasmic	0	0	OUT: 0	
1573	✗	✗	✓	✗	✓	✓
	32	Cytoplasmic	24	0	OUT: 0	
1570	✗	✗	✓	✓	✓	✓
	16	CytoplasmicMembrane	16	113	OUT: 0	
1571	✓	✗	✗	✗	✓	✓
	0	Cytoplasmic	0	0	OUT: 0	
1568	✓	✗	✗	✗	✓	✓

	0	Unknown	0	0	IN: 1	
1569	✓	✗	✗	✗	✓	?
	0	CytoplasmicMembrane	0	0	IN: 1	
1548	✓	✗	✓	✗	✓	✗
	0	Cytoplasmic	1	0	IN: 0	
1549	✓	✗	✗	✗	✓	✓
	0	Unknown	0	0	OUT: 0	
1550	✓	✗	✗	✗	✓	✗
	0	Unknown	0	0	IN: 1	
1551	✓	✗	✓	✗	✓	✓
	0	Unknown	8	0	OUT: 0	
1544	✓	✗	✗	✗	✓	?
	0	Unknown	0	0	OUT: 1	
1545	✓	✗	✓	✗	✓	✓
	0	Cytoplasmic	2	0	OUT: 0	
1546	✓	✗	✗	✗	✓	?
	0	Cytoplasmic	0	0	OUT: 0	
1547	✓	✗	✓	✗	✓	✓
	0	Cytoplasmic	1	0	IN: 1	
1540	✓	✗	✓	✗	✓	✓

	0	Cytoplasmic	1	0	OUT: 0	
1541	✓	✗	✗	✗	✓	✗
	0	Unknown	0	0	OUT: 0	
1542	✓	✗	✗	✗	✗	✓
	0	Cytoplasmic	0	0	IN: 2	
1543	✓	✗	✓	✗	✓	✗
	0	Unknown	1	0	OUT: 1	
1536	✓	✗	✓	✗	✓	✓
	0	Cytoplasmic	20	0	OUT: 0	
1537	✓	✗	✗	✗	✓	✓
	0	Cytoplasmic	0	0	OUT: 0	
1538	✓	✗	✓	✗	✓	✓
	0	Cytoplasmic	2	0	OUT: 0	
1539	✗	✗	✓	✗	✓	✓
	1	Cytoplasmic	2	0	OUT: 0	
1565	✓	✗	✗	✗	✓	✓
	0	Unknown	0	0	IN: 0	
1564	✓	✗	✗	✗	✓	✗
	0	Unknown	0	0	OUT: 0	
1567	✓	✗	✗	✗	✓	✓

	0	Cytoplasmic	0	0	OUT: 0	
1566	✓	✗	✗	✗	✓	✗
	0	Unknown	0	0	OUT: 0	
1561	✓	✗	✗	✗	✓	?
	0	Cytoplasmic	0	0	OUT: 0	
1560	✓	✗	✗	✗	✓	✓
	0	Cytoplasmic	0	0	OUT: 0	
1563	✓	✗	✓	✗	✗	✓
	0	CytoplasmicMembrane	1	0	IN: 3	
1562	✓	✗	✗	✓	✗	?
	0	CytoplasmicMembrane	0	1	OUT: 4	
1557	✓	✗	✓	✗	✓	?
	0	Unknown	1	0	OUT: 1	
1556	✓	✗	✓	✗	✓	✓
	0	Cytoplasmic	1	0	OUT: 0	
1559	✓	✗	✗	✗	✗	✗
	0	CytoplasmicMembrane	0	0	IN: 9	
1558	✓	✗	✗	✗	✓	?

	0	Unknown	0	0	OUT: 0	
1553	✓	✗	✗	✗	✓	✓
	0	Cytoplasmic	0	0	OUT: 0	
1552	✓	✗	✗	✓	✓	✓
	0	Cytoplasmic	0	8	OUT: 0	
1555	✓	✗	✓	✗	✗	✓
	0	CytoplasmicMembrane	1	0	OUT: 18	
1554	✓	✗	✗	✗	✓	✗
	0	Unknown	0	0	OUT: 0	
1576	1452	65	667	267	1254	1273
Prioritized Proteins: 10						

Epitope Analysis

Seq. Id	B Cell Epitope	T Cell Epitope	Location	Log Score	Score	MHC-I Allele Count	MHC-II Allele Count
3	WLDGVYDINENNGIKAYYQY	YDINENNGI	278	3.69	40.0	7	5
	PFVSPFQFILDARYNWRKTT	FVSPFQFIL	704	4.4	81.27	24	5
		FQFILDARY	708	4.61	100.0	18	5
		FILDARYNW	710	172.72	172.72	4	8
	VLTYFNYQRSFVPPQLDVLS	VLTYFNYQR	568	-1.83	0.02	21	4
		YQRSFVPPQ	574	0.88	2.4	3	31

		LTYFNYQRS	569	2.58	13.2	3	1
		YFNYQRSFV	571	119.6	119.6	12	10
	PSFIKSLGNNLLYNTYVRSG	FIKSLGNNL	223	0.74	2.1	40	6
		IKSLGNNLL	224	6.21	500.0	20	15
	DYSGQVNLGYSGITAPKSWQ	YSGQVNLGY	44	1.32	3.75	50	3
		YSGITAPKS	52	147.09	147.09	5	6
	SYGGAEYFTQHFDTVEAGAR	FDTVEAGAR	598	149.26	149.26	6	2
	EAGARYTYKDKFSFNADYFR	YTYKDKFSF	607	0.41	1.5	63	20
		YKDKFSFNA	609	4.46	86.8	35	4
	GSRTVISNKALTQQANQSIE	LTQQANQSI	81	1.93	6.92	40	1
	APKSWQDEEVKKYTGSRTVI	WQDEEVKKY	61	1.32	3.75	72	1
		VKKYTGSRT	66	4.61	100.0	9	3
	GGMINKHVGIQAQANWVRGQ	IQAQANWVR	249	-1.43	0.02	66	1
		INKHVGIQA	243	113.39	113.39	11	4
	FGDLDKVGGTFSFTYYGQLM	VGGTFSFTY	343	-0.47	0.5	84	4
		FSFTYYGQL	347	1.78	5.91	216	2
	NSPSNISNYWLDGVYDINEN	YWLDGVYDI	272	106.1	106.1	130	13
	PLTDLNGDVLKGTSYNKHFP	VLKGTSYNK	692	-0.22	0.03	56	1
	PPDKTGDPSFIKSLGNNLLY	FIKSLGNNL	223	0.74	2.1	150	6
		IKSLGNNLL	224	6.21	500.0	60	15
	WNIQVSQIFWENGRHRVTGS	WNIQVSQIF	785	0.69	2.0	128	20
		IFWENGRHR	792	1.1	3.0	16	2
